# Studies on the Complexation of Platinum(II) by Some 4-Nitroisothiazoles and the Cytotoxic Activity of the Resulting Complexes

**DOI:** 10.3390/molecules31010034

**Published:** 2025-12-22

**Authors:** Andrzej Regiec, Joanna Wietrzyk, Magdalena Milczarek, Andrzej Kochel, Henryk Mastalarz

**Affiliations:** 1 Department of Organic Chemistry and Drug Technology, Faculty of Pharmacy, Wrocław Medical University, 211A Borowska Street, 50-556 Wrocław, Poland; 2 Hirszfeld Institute of Immunology and Experimental Therapy, Polish Academy of Sciences, 12 Rudolf Weigl Street, 53-114 Wrocław, Poland; joanna.wietrzyk@hirszfeld.pl (J.W.); magdalena.milczarek@hirszfeld.pl (M.M.); 3 Faculty of Chemistry, The University of Wrocław, 14F Joliot-Curie Street, 50-383 Wrocław, Poland; andrzej.kochel@uwr.edu.pl

**Keywords:** 4-nitorisothiazoles-Pt(II) complexes, synthesis, spectral analysis, structural analysis, X-ray crystallography, thermochemistry, cytotoxic activity, normoxia, hypoxia, L-glutathione (GSH)

## Abstract

Five novel platinum(II) complexes **C1**–**C5** were synthesized in the reaction of the appropriate substituted 4-nitroisothiazoles with K_2_PtCl_4_ and characterized with elemental analysis, ESI MS spectrometry, NMR spectroscopy, and IR spectroscopy. Also, a new methyl 3-methyl-4-nitroisothiazole-5-carboxylate (**L2**) was obtained. The structures of *trans* complex **C4** and the new isothiazole derivative **L2** were additionally confirmed by X-ray diffraction (XRD) method. The cytotoxicity of the investigated complexes was examined in vitro on three human cancer cell lines (MCF-7 breast, ES-2 ovarian, and A549 lung adenocarcinomas) in both normoxic and hypoxic conditions. The tested complexes, except for the most polar *cis* **C5**, which appeared to be the least active, showed cytotoxic activity comparable to that of the reference cisplatin. *cis*-complex **C1**, *trans* **C2**, and *trans* **C3** showed slightly better cytotoxic activity than cisplatin against the MCF-7 cell line. The complexes had the weakest effect on the A549 cell line. No differences in the cytotoxic activity of the complexes were observed between normoxic and hypoxic conditions, except for the A549 cell line, where all the complexes, except for **C2**, were inactive in hypoxia. However, most complexes, including the reference cisplatin, were equally toxic to healthy BALB/3T3 cells and cancer cells. The *trans* complex **C2** (isomeric to *cis* **C1**) showed even greater toxicity to healthy cells than to MCF-7 and A549 cancer cells. Some complexes were tested for stability against glutathione (GSH) solution to gain additional information that may facilitate the explanation of the pharmacological activity of the tested compounds. Additionally, some theoretical calculations on the thermochemistry of the complexation process were performed using quantum density functional theory (DFT), which indicate that complexation should occur through the coordination of the platinum cation by the nitrogen rather than the sulfur atom of the isothiazole ring.

## 1. Introduction

Anticancer platinum compounds are typically square-planar Pt(II) complexes composed of two leaving ligands, typically chloride or carboxylate anions, and two non-leaving ligands, typically ammonia or primary amine molecules, coordinated to a central Pt(II) atom. These compounds owe their anticancer properties to their ability to form intra- and interstrand Pt-DNA adducts, which are formed by the exchange of departing ligands for N-atoms of purine bases within the DNA strand. Unfortunately, insufficient solubility, developing drug resistance, and numerous side effects often limit the clinical use of platinum(II) complexes. Therefore, numerous works have been conducted on structural modifications of these types of compounds to overcome these limitations, leading to the development of subsequent generations of platinum drugs. The first-generation platinum drug is cisplatin (*cis*-diamminodichloroplatinum), discovered by Michele Peyrone in 1844. After a century of its isolation, Barnett Rosenberg accidentally discovered in 1965 its ability to inhibit bacterial cell division [1]. Cisplatin entered clinical trials in 1971, and the FDA approved it as a drug in 1978 [2]. Cisplatin soon revolutionized human cancer chemotherapy. The approval of cisplatin may be considered as a milestone in the development of chemotherapy, as it improved the survival rates of many oncological patients and promoted the development of its analogs [3]. Introduction of Pt drugs has increased the survival rate and has resulted in substantial therapeutic outcomes for many cancer types, including testicular, lung, colon, breast, and cervical cancers [4]. Second-generation platinum drugs are cisplatin derivatives in which the ammine ligands have been replaced by mono- or bidendate amine ligands, which modify the steric and kinetic properties of the molecule, and the chloride ligands have been replaced by carboxylate ligands, which primarily modify reactivity towards DNA. Examples of second-generation platinum drugs include carboplatin, oxaliplatin, and nedaplatin [5,6,7]. Carboplatin is the first cisplatin analogue to have a cyclobutyldicarboxylate ligand as a leaving group instead of chloride ones. Compared to cisplatin, which provided better therapeutic results but was characterized by higher toxicity, carboplatin had a more favorable toxicity profile and fewer side effects [8]. Because patients tolerated carboplatin better, it can be used at significantly higher doses than cisplatin, often resulting in higher treatment efficacy [9]. Nedaplatin has the same coordinated ammino groups as cisplatin, but it has a different leaving group, in the form of a glycolic acid dianion chelating a Pt ion as a bidendate anionic ligand [7] Nedaplatin has been found to have better anticancer efficacy than carboplatin, as well as lower toxicity and fewer side effects than cisplatin [10]. Oxaliplatin is constructed differently, by replacing the two ammine ligands in cisplatin with a bidendate ligand, (1R,2R)-cyclohexane-1,2-diamine [6]. It was the first drug approved for the treatment of cisplatin-resistant tumors [11]. Oxaliplatin’s side effects are also less severe than those of cisplatin due to the oxalate anion as a leaving group, making it less toxic [12]. Third-generation platinum drugs are improved forms of older cisplatin analogues. The most promising of these are lobaplatin and heptaplatin. Lobaplatin is a platinum(II) complex in which the lactic acid anion is the leaving group and the non-leaving bidendate amine ligand is 1,2-bis(aminomethyl)cyclobutene [13]. Heptaplatin with malonate dianion as a leaving group and 2-isopropyl-1,3-dioxolane-4,5-dimethanamine as a bidendate non-leaving ligand is used in cancer therapy due to its high chemical stability, lower toxicity than cisplatin, and effectiveness against cisplatin-resistant cancer cells [13].

New platinum complexes with increased binding specificity to molecular targets in cancer cells or exhibiting novel mechanisms of anticancer action appear to be a viable strategy for overcoming the challenges of cancer treatment with platinum drugs. Many different types of new platinum complexes have been investigated, but most were designed by replacing the amine and/or chloride ligands of the leaving groups compared to cisplatin. All of them have a steric *cis* configuration, making them suitable for consideration as simple cisplatin analogs. Due to this structural similarity, these complexes also share a similar mechanism of anticancer action through interaction with DNA [14,15]. Such modifications are unlikely to lead to a breakthrough in the effectiveness of platinum-based cancer treatment. Consequently, relatively few such compounds have been successfully introduced so far into clinical practice [16].

This work is our latest attempt to obtain active anticancer platinum complexes that are not simple analogues of cisplatin. It should be considered as a continuation of our studies on the complexation of platinum by ligands from the nitro-1,2-azole group and testing the anticancer activity of the resulting complexes. Our previous work was focused on complexes of platinum with nitropyrazoles and nitroisoxazoles [17,18]. In these studies [17,18], we obtained and described a series of platinum(II) complexes with the general formula PtCl_2_L_2_, where L is a substituted nitropyrazole or nitroisoxazole ligand. Cytotoxicity measurement on a panel of three cancer cell lines revealed significant activity of *trans*-[PtCl_2_ (1-methyl-4-nitropyrazole)_2_] and *trans*-[PtCl_2_ (3,5-dimethyl-4-nitroisoxazole)_2_], which demonstrated the ability to half-inhibit cancer cell growth at micromolar concentrations, significantly exceeding cisplatin. The corresponding *cis*-congeners of these active compounds showed significantly lower activity. We also measured the reaction rates of the obtained compounds with glutathione to initially assess their susceptibility to inactivation in cancer cells, especially those with developed drug resistance, by forming adducts with endogenous thiols and high-molecular-mass platinophiles [19]. This work is a continuation of our studies of Pt(II) nitroazole complexes, and now, we have focused on the synthesis and evaluation of the pharmacological activity of derivatives with 4-nitroisothiazole ligands. This choice was based on the results of our previous studies, which indicated that the cytotoxicity of nitroazole complexes with Pt(II) requires the presence of a heteroatom–heteroatom bond in the ligand molecule. To date, there are no reports in the literature of studies investigating the anticancer properties of Pt(II) complexes with nitroisothiazoles as non-leaving ligands. As we announced in the previous work [18], the current one addresses the complexation of platinum by 4-nitroisothiazoles. Some nitroisothiazole platinum complexes were the subject of two patents from the 1980s [20] and 1992 [21], but they lack details on the results of biological tests for anticancer activity and also a description of the physicochemical, especially spectral, properties of these complexes. They also lack evidence to confirm the structure in terms of *cis*-*trans* isomerism. To date, structural studies of these complexes are also lacking, as it should be emphasized that platinum can also potentially complex through the free sulfur electron pair of the isothiazole ring system.

## 2. Results and Discussion

### 2.1. Theoretical Considerations on the Complexation of 4-Nitroisothiazoles by Platinum Ion Pt^2+^

**Figure 1:**
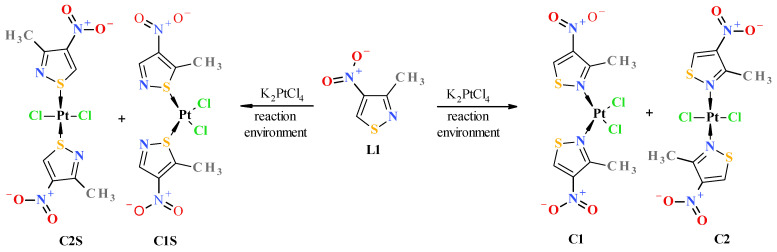
Possible reaction products of platinum(II) potassium chloroplatinate with 3-methyl-4-nitroisothiazole (**L1**) as a result of complexation of platinum (II) by nitrogen atom of the isothiazole ring (potential products **C1** and **C2** of complexation reaction 1) and competitive complexation by sulfur atom of the isothiazole ring (potential products **C1S** and **C2S** complexation reaction 2).

Since the isothiazole ring contains, apart from the nitrogen atom, also sulfur in its structure, which according to HSAB theory [22,23] is soft, it is possible for the platinum(II) ion (which according to this theory is moderately soft) to complex with sulfur, competitively with the much harder nitrogen (Figure 1). The platinum (II) ion has a particularly strong affinity for sulfur at the negative second oxidation state, as in thiols, for example. In contrast, sulfur in the isothiazole ring is formally at the zero oxidation state, which theoretically should reduce its affinity for the platinum ion. For theoretical considerations, platinum complexes with 3-methyl-4-nitroisothiazole (**L2**) were taken as an example, and Gaussian 16 software (Revision C.02) was used for the calculations [24].

To determine which complexes should form in the reaction of isothiazole complexation by platinum ions, Gibbs energies (ΔG_r_) were theoretically estimated for the reaction of ligand **L1** with tetrachloroplatinate towards the formation of platinum complexes through nitrogen–platinum bonding, i.e., N-Pt bonded *cis* complex **C1** and its *trans* isomer **C2** (here, ΔG_r1_ of complexation reaction 1), and additionally, towards the formation of platinum complexes formed through sulfur–platinum bonding, i.e., S-Pt bonded **C1S** and **C2S** (here, ΔG_r2_ of complexation reaction 2) (Figure 1). Since the compounds under consideration are mutually isomeric, the differences between the Gibbs energies of these complexation reactions (ΔG_r1_ for reaction 1 and ΔG_r2_ for reaction 2) are identical to the differences between the formation Gibbs energies of the respective complexes (i.e., ΔG_r1_ − ΔG_r2_ = Δ_f_G_c1_ − Δ_f_G_c1S_ = ΔΔ_f_G, where Δ_f_G_c1_ and Δ_f_G_c1S_ are formation Gibbs energies of **C1** and **C1S** complex, respectively). The calculations were conducted using quantum DFT methods with various combinations of functionals and basis sets, also taking into account solvent effects (such as a water–acetone mixture for reaction environment simulation). For the simulation solvent environment, the SMD (Solvation Model Density) model of SCRF (Self-Consistent Reaction Field) was used [25]. Theoretical estimated differences between the Gibbs energy of the formation (ΔΔ_f_G) of the respective complexes (i.e., N-Pt bonded *cis*-complex **C1** versus S-Pt bonded *cis*-complex **C1S** and N-Pt *trans*-complex **C2** versus S-Pt *trans*-complex **C2S**) were significantly large and, depending on the calculation method, were in the range of (−ΔΔ_f_G) 19–48 kcal/mol in favor of the stability of platinum–nitrogen bonded complexes **C1** and **C2** (Table 1). Additional information on performed calculations can be found in Section 3.4 Computational Details.

**Figure 2:**
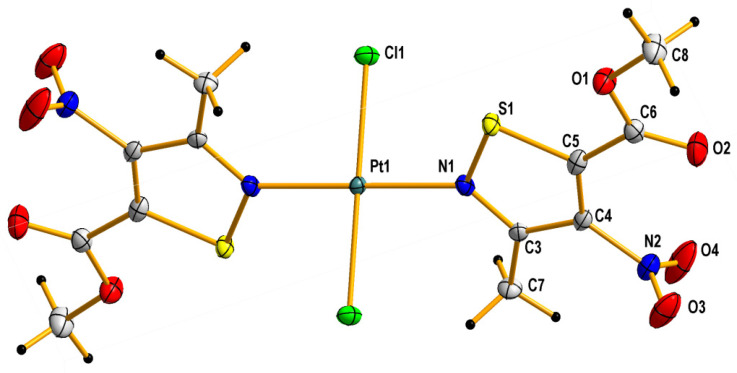
Crystal structure of *trans*-dichlorobis(5-carbomethoxy-3-methyl-4-nitroisothiazole)platinum(II) (**C4**). The displacement ellipsoids are drawn at the 50% probability level.

Thus, this indicates that platinum complexes formed through nitrogen–platinum bonding are thermodynamically much more stable than those formed through the sulfur one. In fact, the results of XRD studies (see Figure 2 and Section 3.3 for details) confirm the theoretical prediction that complexation of nitroisothiazoles by platinum occurs via the nitrogen atom rather than the sulfur atom. In addition, calculated, at B3LYP/aug-cc-pVTZ/DZP theory level, differences between the formation Gibbs free energies for *cis* and *trans* complexes (**C1** versus **C2**), which can be described as the Gibbs energy of the *trans*-to-*cis* isomer conversion (Δ_f_G_cis_ − Δ_f_G_trans_ = ΔG_isom._), are 7.05 kcal/mol and 1.75 kcal/mol for, respectively, vacuum and water–acetone (this mixture in volume 2:1 ratio was actually used to carry out the reaction between K_2_PtCl_4_ and ligand **L1**) environment which indicates that *trans* isomer **C2** is more thermodynamically stable than *cis* isomer **C1**, and this energy difference (isomerization energy (ΔG_isom._), in other words) is strongly dependent on reaction environment. Consequently, this energy and the resulting values of trans/cis equilibrium constant (K_isom._) decrease with increasing polarity of the solvent, which may be due to the greater polarity of the *cis* complex compared to its *trans* isomer, resulting in the more polar compound **C1** being more stabilized in the more polar solvent (Table 2).

**Figure 3:**
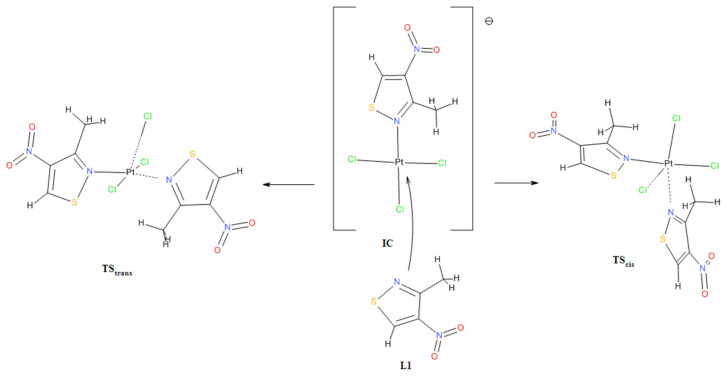
Probable structures of transition states **TS_cis_** and **TS_trans_** forming during reaction of ionic complex **IC** with ligand **L1**.

In fact, the reaction between ligand **L1** and potassium tetrachloroplatinate in a water–acetone mixture in a volume ratio of 2:1 resulted in a mixture of *cis* **C1** and *trans* **C2** isomers in a molar ratio of approximately 2:1 in favor of the *cis* isomer (see experimental part). This result may indicate that, despite the calculated greater thermodynamic stability of the *trans* isomer **C2**, the outcome of the reaction may be determined by the activation energy, i.e., the energy of the corresponding transition state towards the formation of a given isomer. The preliminary theoretical estimate of the activation energy (as Gibbs free energy, ΔG^#^) at B3LYP/aug-cc-pVTZ/DZP theory level, using the similar methodology described in [28], for the formation of the *cis* isomer **C1** was 19.72 kcal/mol and for the *trans* isomer **C2** 21.15 kcal/mol, which gives a difference of approximately 1.4 kcal/mol in favor of the formation of the *cis* isomer (presented values are for calculation performed for isolated molecules in vacuum environment). Corresponding values for water–acetone (2:1 vol.) solvent mixture simulation are, respectively, as follows: 25.75 and 28.43 kcal/mol, which gives a difference which is approximately 2.6 kcal/mol in favor of the formation of the *cis* isomer. Activation energy ΔG^#^ was calculated as the difference between the Gibbs energy of the corresponding transition state (**TS_cis_** and **TS_trans_**) and the sum of the Gibbs energy of the ionic complex **IC** and ligand **L1** (Figure 3). The ionic complex **IC** is an intermediate product that must be formed first in the reaction of ligand **L1** with chloroplatinate and is a substrate that can react with another ligand **L1** to form both the *cis* **C1** and *trans* **C2** complexes. The designed transition state structures (**TS_cis_** and **TS_trans_**) were optimized and validated by confirming the presence of a single imaginary wave number through oscillatory calculations (freq) and then checked to see if they were a saddle point connecting substrates and products through intrinsic reaction coordinate (IRC) calculations [29].

### 2.2. Reactions of Nitroisothiazoles with Potassium Tetrachloroplatinate and Structural Analysis of Resulting Pt-Complexes

**Figure 4:**
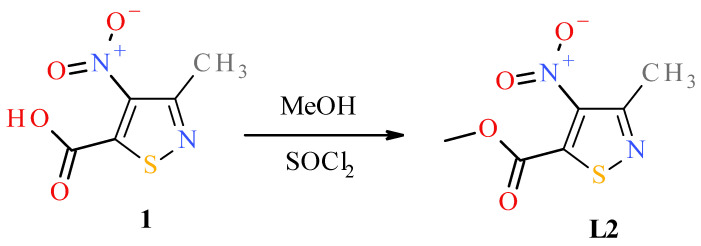
Scheme of synthesis of methyl 3-methyl-4-nitroisothiazole-5-carboxylate (**L2**).

To obtain the desired nitroisothiazole complexes, we synthesized two known 4-nitroisotiazole derivatives according to the previously described procedures, namely, 3-methyl-4-nitroisothiazole (**L1**), 3-methyl-4-nitroisothiazole-5-carbonitrile (**L3**) [30], and one undescribed methyl 3-methyl-4-nitroisothiazole-5-carboxylate (**L2**), which were used as ligands for complexation reactions. Ester **L2** was obtained from known 3-methyl-4-nitro-isothiazole-5-carboxylic acid (**1**) [30] in an esterification reaction with anhydrous methanol through its acid chloride (Figure 4, see Section Preparation of Methyl 3-Methyl-4-nitro-5-isothiazolecarboxylate (**L2**) for details).

**Figure 5:**
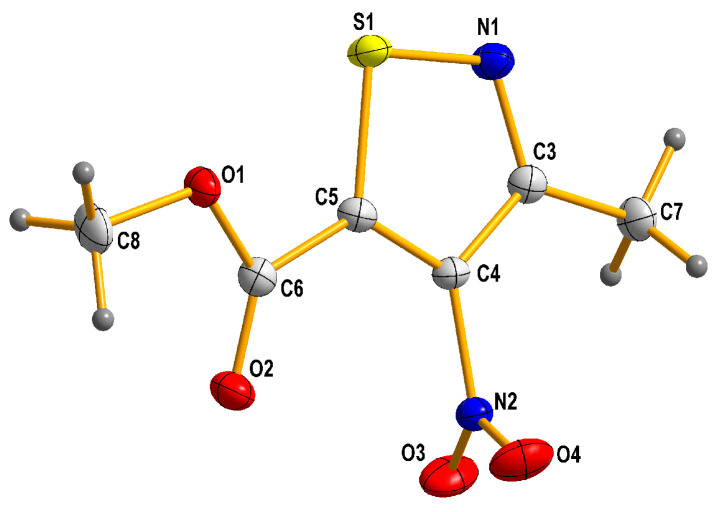
Molecular structure of methyl 3-methyl-4-nitroisothiazole-5-carboxylate (**L2**). (Thermal ellipsoids are plotted at a 50% probability level.).

The structure of new methyl 3-methyl-4-nitroisothiazole-5-carboxylate (**L2**) was proven and characterized by appropriate spectroscopic measurements, such as ^1^H, ^13^C NMR, MIR, and crystallographic data with the use of the single crystal X-ray diffraction (XRD) method (Figure 5 and crystallographic data collected in Tables S1–S6 in the Supplementary Materials).

**Figure 6:**
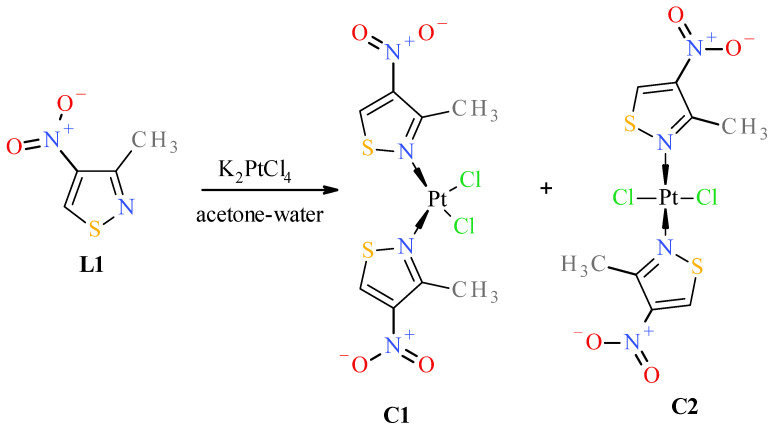
Scheme of reaction of 3-methyl-4-nitroisothiazole (**L1**) with K_2_PtCl_4_.

**Figure 7:**
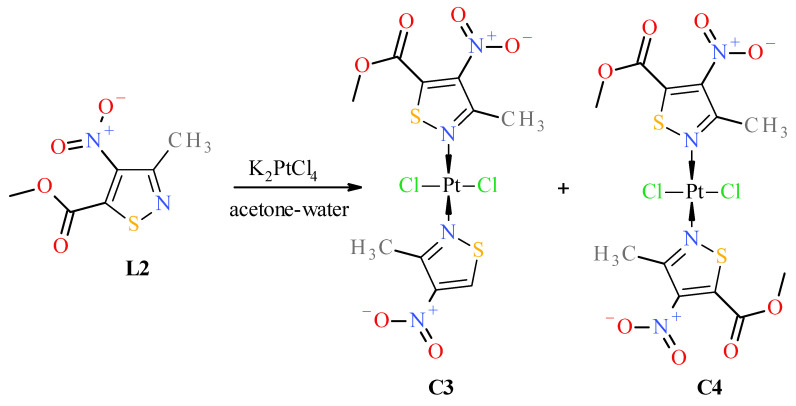
Scheme of reaction of methyl 3-methyl-4-nitroisothiazole-5-carboxylate (**L2**) with K_2_PtCl_4_.

**Figure 8:**
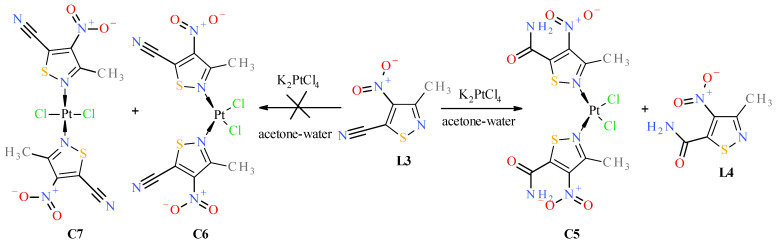
Scheme of reaction of 3-methyl-4-nitroisothiazole-5-carbonitryl (**L3**) with K_2_PtCl_4_.

Reactions of K_2_PtCl_4_ with appropriate nitroisotiazole ligands have been conducted at room temperature in light protection (Figure 6, Figure 7 and Figure 8). The progress of the reaction was monitored by TLC chromatography. Details on the handling, separation, and purification of products can be found in the experimental Section 3. As a result of the reactions carried out, five new complexes, **C1**–**C5**, were isolated from the reaction mixtures and identified. All novel platinum(II) complexes **C1**–C**5** were characterized by ^1^H, ^13^C NMR, MIR, far-IR, and MS-ESI spectroscopy. Additionally, a ^1^H-^13^C 2D HMBC (Heteronuclear Multiple Bond Correlation) spectrum was obtained for *cis*-complex **C5**, which facilitated the assignment of chemical shifts in the ^13^C-NMR spectrum (see Experimental Section 3 Methods and Materials). Furthermore, *trans* complex **C4** was analyzed by X-ray diffraction (XRD) (Figure 2 and crystallographic data collected in Tables S7–S11 in the Supplementary Materials).

Both isomeric complexes (i.e., *cis* **C1** and *trans* **C2**, Figure 6) were obtained only in the case of the reaction of 3-methyl-4-nitroisothiazole (**L1**) with K_2_PtCl_4_. The structure and assignment of the correct configuration to isomers **C1** and **C2** were determined as follows. ESI-MS spectra indicate that both complexes **C1** and **C2** have the same molecular weight and contain a platinum atom (as can be seen from the isotope peaks). Combustion analysis indicates that both compounds have the same elemental composition (same empirical formulas). Combined with the mass spectrum data, this gives the same molecular formulas for both compounds **C1** and **C2.** A conclusion can be drawn that complexes **C1** and **C2** are mutual isomers. Complex **C2** has more shifted methyl group protons (3.25 ppm) than isomer **C1** (3.12 ppm) in ^1^H-NMR. More shifted protons of the methyl groups located in the position adjacent to the nitrogen atom, which is complexed by the platinum atom, are characteristic of *trans* isomers. All *trans* complexes with ligands from the nitro-1,2-azole group (i.e., pyrazoles and isoxazoles) that we have studied so far have always shown this relationship, and we have not encountered any exceptions to this rule [17,18]. This is related to the stronger deshielding of surroundings by the platinum atom in *trans* isomers than in *cis* isomers, which implies that the platinum atom is more shielded by the surroundings in *trans* isomers. This causes protons and methyl group protons to be more strongly deshielded in positions adjacent to complexed nitrogen atoms in *trans* isomers than in *cis* ones, which can be observed in ^1^H-NMR spectra. The absence of splitting of the Pt-Cl stretching vibration bands (only antisymmetric vibration) in the far IR spectrum (appearing in 360–335 cm^−1^ range) further confirms that complex **C2** (345 cm^−1^ (ν_as_Cl-Pt)) is a *trans*-isomer, and the split band (symmetric and antisymmetric vibrations) indicates that complex **C1** (343 cm^−1^ (ν_s_Cl-Pt), 337 cm^−1^ (ν_as_Cl-Pt)) is a *cis*-isomer. Compound **C1** has a lower value of retardation factor (R_f_) in thin-layer chromatography (TLC) than its isomeric compound **C2** (see Table 3, visualization of TLC chromatogram plates has been included in Supplementary Material, Figures S48 and S49). *cis* complexes with the structure *cis*-PtCl_2_L_2_ (where L is a ligand and both are identical) always have non-zero dipole moments, unlike their *trans* isomers (*trans*-PtCl_2_L_2_), which always have zero dipole moments. Therefore, *cis* complexes with the above structure are more polar than their *trans* isomers. All *cis* isomers of pyrazoles and isoxazoles previously studied by us always, without exception, had lower R_f_ values than their *trans* isomers, which always had higher R_f_ values, so we have never noticed, among a pair of *cis*–*trans* isomeric complexes, the *trans* isomer with a zero dipole moment having R_f_ lower than the *cis* compound with the same surface area and the same substituents but with a nonzero dipole moment, which is associated with a greater polarity of its molecule. All the above proofs (albeit indirect) are sufficient to correctly assign the *cis* configuration to compound **C1** and the *trans* configuration to compound **C2**.

Surprisingly, no formation of the *cis* isomer with ester ligand **L2** was observed. Instead, two complexes, *trans* **C3** and *trans* **C4**, were obtained in the reaction with ester **L2** (Figure 7). The structure of *trans* complex **C4** was unambiguously determined with XRD measurement (Figure 2). Analysis of NMR, IR, and MS spectra has shown that complex **C3** has mixed ligands, which indicate that one of the ligands of the *trans* complex **C4** undergoes hydrolysis and subsequent decarboxylation under the reaction conditions. The tendency for the acid **1** to undergo easy decarboxylation is known from previous studies [30]. The acid **1** undergoes slow decarboxylation to 3-methyl-4-nitroisothiazole (**L1**) during storage at room temperature and very rapid decarboxylation at elevated temperatures. The configuration of *trans* complex **C3** was established by comparison of methyl group proton shifts in ^1^H-NMR spectra of *trans*-complexes **C2** and **C4**, whose structures are unambiguous. Methyl group protons for ligand **L2** of complex **C3** have the same shift (i.e., δ = 3.12 ppm) as in *trans*-complex **C4** (δ = 3.12 ppm), and methyl group protons for ligand **L1** of complex **C3** have nearly the same shift (i.e., δ = 3.24 ppm) as in *trans*-complex **C2** (δ = 3.25 ppm). It was surprising that during the reaction of potassium tetrachloroplatinate with nitrile **L3**, a *cis* complex **C5**, instead of the expected Pt-nitrile **L3** complexes **C6** and **C7** (Figure 8), was formed (precipitated from the reaction solution) in which the nitrile ligand was converted into a known 3-methyl-4-nitroisothiazole-5-carboxamide (**L4**) [30]. In addition, only one isomer, **C5** (i.e., *cis* one), was separated from the reaction mixture (Figure 8). No *trans* isomer with amide ligand **L4** was found in the post-reaction mixture. Configuration *cis*-isomer **C5** was determined on the basis of the Far IR spectroscopy, which is very useful for distinguishing *cis* from *trans* isomers for platinum dihalogen complexes, because in the range (at about 340–360 cm^−1^) they exhibit strong Pt-Cl bond vibration bands [31]. In the Far-IR spectrum of complex **C5**, the band of the stretching vibration of both Pt-Cl bonds was split due to active symmetric (at 344 cm^−1^) and antisymmetric (at 339 cm^−1^) vibrations of these bonds. In the case of the *trans* isomer, only the antisymmetric vibration of these bonds is active due to the change in dipole moment, while the symmetric vibration is inactive because the dipole moment does not change in this case. In the ^1^H-NMR spectrum of complex **C5**, the shift for the protons of the methyl group at third position of isothiazole is 3.19 ppm and the corresponding shift for the methyl group of free amide ligand **L4** is 2.65 ppm which results from the strong influence of the platinum ion on the weakening of the shielding of the protons of the methyl group located in close proximity, similarly to other complexes. It is worth noting that in both amide ligand **L4** and *cis*-complex **C5**, there is a very strong intramolecular hydrogen bond between one proton of the amide group and the oxygen of the nitro group, which manifests itself in a large difference in shifts in the ^1^H-NMR spectrum between the two protons of the amide group, i.e., Δδ = 0.34 ppm and 0.29 ppm for amide **L4** and its Pt-complex **C5**, respectively.

As mentioned above, in the case of the ^1^H-NMR spectroscopy of all complexes, the strong effect of electron deshielding of the methyl protons of the isothiazole ring caused by electron withdrawal by the central platinum ion is clearly visible, which is in line with expectations. This effect is stronger in the case of *trans* complex **C2** than in the case of the corresponding *cis* isomer **C1** (see discussion above and the values of chemical shifts in Section 3.2.2 of the experimental part and visualization of the spectra in Figures S3 and S5 in the Supplementary Materials). The same effect was observed in the case of platinum complexes with nitropyrazoles and nitroisoxazoles [17,18]. If we are certain that the complexes under investigation are a pair of *cis* and *trans* isomers (like *cis* **C1** and *trans* **C2**), a good and quick way to distinguish between the two is to measure their R_f_ value using TLC (Thin Liquid Chromatography). *cis* complexes are significantly more polar due to their non-zero dipole moment, unlike *trans* isomers, which have zero dipole moments (provided that both ligands are the same) and are therefore more lipophilic than *cis* isomers (see Table 3). The most lipophilic is the *trans* **C4** complex (with two ester ligands), slightly less *trans* **C3** (with one ester ligand), followed by *trans* **C2** (with 3-methyl-4-nitroisothiazole (**L1**) ligand), then its *cis* isomer **C1**, and least of all *cis*-complex **C5** with a hydrophilic amide ligand.

The complexes **C1**–**C5** studied are thermally stable. They undergo slow thermal decomposition above 130 °C in the case of complex **C3** and above 200 °C in the other cases (**C1**, **C2**, **C4,** and **C5**). When stored at room temperature, away from light, they do not undergo any noticeable changes. Complexes **C1**–**C4** are slightly soluble in acetone and very poorly soluble in chloroform. The most polar complex **C5**, on the other hand, is very poorly soluble in acetone, slightly better in acetonitrile, and practically insoluble in chloroform. Their acetone solutions are stable, and no isomerization or decomposition of the complexes has been observed, even after storing the solutions at room temperature for a month (protected from light), which was confirmed by TLC chromatography and ^1^H-NMR spectroscopy. They are unstable in dimethylsulfoxide (DMSO) and dimethylformamide (DMF) solutions due to decomposition and isomerization.

### 2.3. In Vitro Cytotoxic Activity

The cytotoxicity of the complexes **C1**–**C5** was estimated by examining cell viability using the sulforhodamine B assay. Due to the poor solubility of the compounds in water for in vitro cytotoxicity study, for subsequent dilutions in biological medium, initial solutions of the **C1**–**C4** complexes were prepared in acetone because the compounds in acetone were stable and did not decompose, similarly to our previous work on nitro-1,2-azole complexes [17,18]. However, due to the very poor solubility of the most polar complex **C5** in acetone, its initial solution had to be prepared in polar *N*,*N*-dimethylacetamide (DMA) (see Section 3.7). Cisplatin was used as the reference drug. The results are presented as IC_50_ (50% inhibitory concentration), i.e., concentrations that reduce cell survival by 50%. The test was conducted on three cancer cell lines and one normal healthy cell line, namely, breast adenocarcinoma (MCF-7), ovarian adenocarcinoma (ES-2), lung adenocarcinoma (A549), and murine embryonic fibroblast (BALB/3T3). The results are presented in Table 4.

Three complexes, namely, *cis*-complex **C1**, *trans* **C2**, and *trans* **C3**, showed slightly better cytotoxic activity than cisplatin against the MCF-7 cell line (Table 4). The polar complex **C5** (with an amide ligand **L4**) showed the weakest activity against MCF-7. The most lipophilic *trans*-complex **C4** showed cytotoxicity against MCF-7 similar to cisplatin. All complexes, except **C5**, showed under hypoxic conditions similar activity to cisplatin, which, moreover, did not differ from that under normoxic conditions. Apart from *trans* **C2**, the complexes studied, including the reference cisplatin, showed similar toxicity towards healthy and cancerous MCF-7 cells. Hence, the selectivity indices (SI), i.e., the ratio of cytotoxicity towards healthy cells to cytotoxicity towards cancer, are similar and equal ≈ 1 for all these compounds. It is noteworthy that the *trans* **C2** complex showed statistically significantly higher toxicity towards healthy cells than towards MCF-7 cells in normoxia, so its SI is very low (≈0.2).

In the case of the ES-2 cancer cell line, under normoxic and hypoxic conditions, all complexes except **C5** showed statistically similar activity to cisplatin. No differences in their cytotoxic activity against ES-2 cells were observed for both conditions, i.e., normoxia and hypoxia. All the complexes **C1**–**C5**, including the reference cisplatin, were similarly toxic towards healthy and ES-2 cancer cells (SI ≈ 1).

Studies of the activity of compounds against the A549 line under normoxic conditions revealed that only complexes **C1**–**C3** showed activity comparable to cisplatin, while complexes **C4** and **C5** were significantly weaker than cisplatin cytotoxicity. Under hypoxic conditions, all complexes tested, apart from *trans* **C2**, which showed activity similar to cisplatin, were inactive against A549 cancer cells. Here, as in the case of the MCF7 cancer cell line, *trans*-complex **C2** showed significantly greater (statistically significant) toxicity to healthy cells than to A549 cancer cells, resulting in a very low SI, which was 0.2.

In summary, on the basis of the results of cytotoxicity studies conducted in vitro, it can be concluded that new platinum complexes with isothiazole ligands have shown the best effects against breast adenocarcinoma cells (MCF-7) and proved to be the least active against lung adenocarcinoma (A549). In addition, they are highly toxic to healthy cells, unlike previously studied platinum complexes with nitropyrazoles, which appeared to be less toxic [17]. In addition, no complex with nitroisothiazole exceeds the anticancer activity of the platinum complex with ntroisoxazole (i.e., *trans*-dichlorobis(3,5-dimethyl-4-nitro-isoxazole)platinum(II)) previously described and tested by us [18].

### 2.4. Reactivity with L-Glutathione (GSH)

Deactivation of platinum anticancer drugs occurs in the organism at both systemic and intracellular levels. After intravenous administration, these drugs undergo a series of metabolic processes, and the concentration of their active forms can be reduced by various mechanisms. At the systemic level, the first step in deactivation is plasma protein binding: Platinum drugs rapidly bind to plasma proteins (over 90%), especially albumin [32]. This binding prevents them from entering cancer cells, significantly reducing their concentration in target tissues. At the cellular level, platinum drugs are neutralized by binding to intracellular proteins and peptides such as glutathione and metallothionein [33]. Binding to peptide and protein thiol and thioether moieties, which are the primary binding sites for such reactions [32,33], prevents the drug from reaching DNA and may be responsible for its nephrotoxicity [34]. As in previous studies [17,18], we assessed the susceptibility of the tested compounds to this type of inactivation in the cancer tissues by measuring the half-life of the compound in an aqueous solution containing a physiological concentration of the reduced form of glutathione (GSH) using UV photometry at a wavelength of 260 nm similarly as in Hagrman et al. [35]. Using the same methodology allowed us to directly compare the GHS reactivity of nitroisothiazole compounds with their pyrazole and isoxazole analogues. As a control, the UV spectra of complexes **C1** and **C2** in dioxane were recorded to check whether there was excessive absorption at a wavelength of 260 nm, which could interfere with the measurement of the reaction of the complexes with glutathione (visualization of the UV spectra can be found in the Supplementary Materials, Figures S46 and S47).

**Figure 9:**
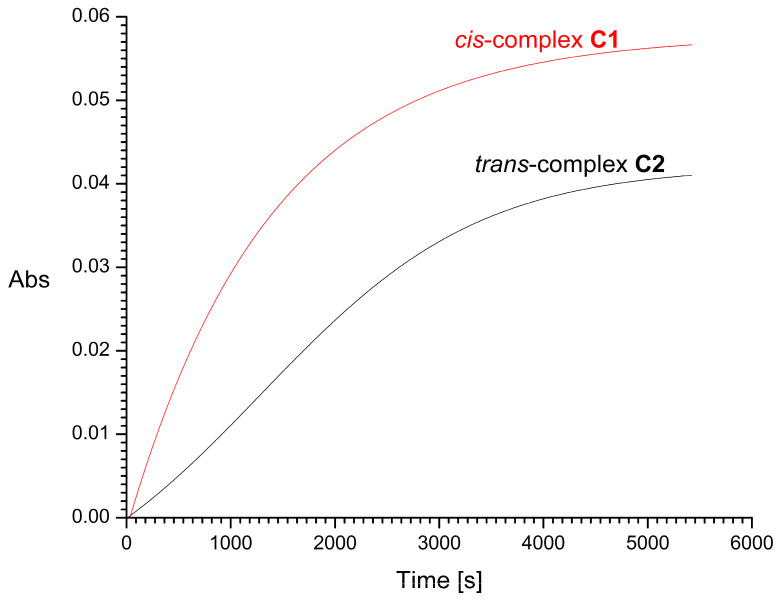
Time dependence of UV absorbance (at 260 nm) of *cis*-complex **C1** (red line) and its *trans*-isomer **C2** (black line) in the presence of 2 mM of L-glutathione (GSH). Abs is absorbance.

**Figure 10:**
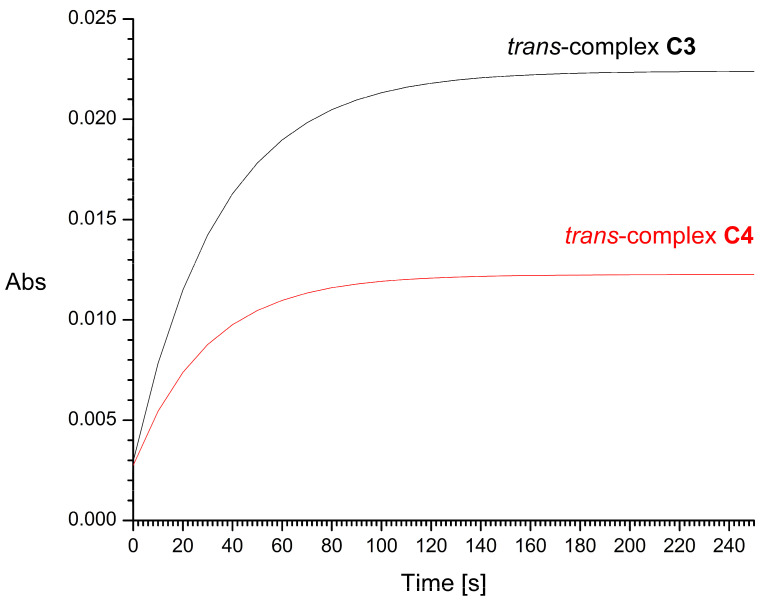
Time dependence of UV absorbance (at 260 nm) of *trans*-complex **C3** (black line) and *trans*-isomer **C4** (red line) in the presence of 2 mM of L-glutathione (GSH). Abs is absorbance.

The half-times of the reaction with 2 mM GSH, resulting mainly in the formation of Pt-S bonds, were found to be about 16 and 29 min for *cis*-complex **C1** and its *trans* isomer **C2**, respectively (Figure 9). For *trans* complexes **C3** and **C4**, the half-times of the reaction with 2 mM GSH were very short, i.e., about 20 and 10 s, respectively (Figure 10). Increasing the concentration of L-glutathione to 4, 8, or 16 mM does not significantly affect the measurement results. For comparison, the half-time of cisplatin in glutathione solution under similar conditions was previously estimated by Hagrman et al. to be approximately 60 min [35] and by Suchankova et al. to be 66 min, while the half-life of the pharmacologically inactive isomeric transplatin in GSH solution was only 4 min [36].

## 3. Methods and Materials

Measurement of the melting points (m.p.) of the obtained compounds was performed with the Büchi M560 melting point device (BÜCHI Labortechnik AG, CH-9230 Flawil/SG, Flawil, Switzerland) and was uncorrected. The ESI-MS (electrospray ionization mass spectroscopy) spectra were recorded with the compactTM Bruker Daltonics Electrospray Ionization Quadrupole Time-of-Flight (ESI-Q-TOF) spectrometer (Bruker Daltonics GmbH, Bremen, Germany). The ESI-MS measurements for new compounds were performed in both positive- and negative-ion mode in LC-MS-grade using LC-MS grade methanol as a solvent. ESI-MS spectral analysis and the simulation of the theoretical monoisotopic mass of detected ions were performed using Bruker Compass Data Analysis 4.2 software (Bruker Daltonics GmbH, Bremen, Germany). All ^1^H and ^13^C-NMR spectra were recorded on the following instruments: Bruker NMR AVANCE III™ 600 MHz spectrometer with Ascend™ technology magnet with a quadruple resonance NMR ‘inverse’ cryoprobe (Bruker Corporation, Billerica, MA, USA) and/or Bruker ARX-300 (300 MHz) spectrometer (Bruker Analytische Messtechnik GmbH, Rheinstetten, Germany). The NMR measurements of samples of the compounds were carried out at room temperature (20–25 °C) using 5 mm tubes in deuterated acetone-d_6_. NMR chemical shifts were referenced to the solvent signal, i.e., for ^1^H-NMR: δ (quintet of residual acetone-d_6_) = 2.05 ppm and for ^13^C-NMR: δ (septet of acetone-d_6_) = 29.84 ppm. The Nicolet iS50 FT-IR spectrophotometer (Thermo Fisher Scientific Inc., Waltham, MA, USA) was used to record the Attenuated Total Reflectance IR (ATR-FT-IR) spectra (at MIR range 4000–450 cm^−1^). Recording of spectra was performed on the diamond crystal surface (32 scans, resolution: 1 cm^−1^, measurement temperature: 20–25 °C) with ATR intensity correction in the Laboratory of Elemental Analysis and Structural Research, Faculty of Pharmacy, Wroclaw Medical University. A Fourier transform, Bruker VERTEX 70V vacuum spectrometer equipped with an air-cooled DTGS detector was used to obtain the Far-IR (far-infrared) spectra (below 500 cm^−1^) of the Pt(II) complexes. The spectra measurements were conducted with the ATR accessory, such as a diamond ATR crystal, in the resolution of 1 cm^−1^ with 64 scans in the Laboratory of Vibrational Spectroscopy of the Faculty of Chemistry at Wrocław University of Science and Technology. No ATR intensity correction was applied to Far-IR spectra. Omnic 9.3.30 and Omnic Specta 2.0 software (Thermo Fisher Scientific Inc.) was used to process and analyze spectra. A double beam U-2800 Spectrophotometer (Hitachi—Science & Technology, Tokyo, Japan) was used to record UV spectra of Pt-complexes (**C1** and **C2**) and reaction rate measurements of the GSH complexes. Dioxane solutions of complexes were used in the following concentrations: c = 2 × 10^−5^ M and 3 × 10^−5^ M, respectively, for **C1** and **C2**. Measurement of UV spectra was conducted in quartz cuvettes (path length = 10.0 mm) in the range 190–400 nm (sampling interval 1 nm, scan speed 200 nm/min). All UV experiments were performed in the Laboratory of Elemental Analysis and Structural Research, Faculty of Pharmacy, Wroclaw Medical University. Baselines were corrected with pure dioxane as the reference solvent. Thin-layer chromatography (TLC) was used to monitor the progress of the reaction course and determine the purity of the synthesized compounds. Silica gel on TLC Al foils with fluorescent indicator 254 nm (Sigma-Aldrich, Merck Group, Darmstadt, Germany) was used as the stationary phase and chloroform–acetone mixtures (volume ratios 9:1, 7:3, and 1:1) as the eluent. Detection of Pt(II) compounds in the chromatograms was performed using a 0.5% acetone solution of rubeanic acid (dithiooxamide), and organic impurities were detected by observing the plates under UV light at 250 nm. Separation and purification of the compounds was performed by flash chromatography using a Pure Chromatography C-900 apparatus (BÜCHI Labortechnik AG, Meierseggstrasse 40, Postfach CH-9230 Flawil 1). Carlo Erba series NA 1500 elemental analyzer (Carlo Erba, Milan, Italy) was used to determine elemental analysis by the combustion method.

### 3.1. Synthesis of 4-Nitroisothiazole Ligands

Ligands, such as 3-methyl-4-nitroisothiazole (**L1**) and 3-methyl-4-nitroisothiazole-5-carbonitrile (**L3**), were prepared according to the previously described methods [30]. For comparison purposes, their ^1^H and ^13^C NMR spectra were included in the Supplementary Materials (Figures S1 and S2 for ligand **L1** and Figures S22 and S23 for ligand **L3**).

#### Preparation of Methyl 3-Methyl-4-nitro-5-isothiazolecarboxylate (**L2**)

A total of 3 g (15.9 mmole) of 3-methyl-4-nitro-5-isothiazolocarboxylic acid [30] was dissolved in 60 mL of anhydrous methanol. The solution was cooled to about −5 °C. Then, 12 mL of SOCl_2_ was dropped. The mixture was kept at −5 °C for 2 h and then was set at room temperature for 24 h. The next day, the excess of methanol and SOCl_2_ was evaporated to dryness. To the residue, 5 mL of distilled water was added, and a solution of NaHCO_3_ was dropped to an alkaline pH. Ester was extracted with diethyl ether three times. The combined ether phases were dried with anhydrous MgSO_4_. Then, ether was evaporated, giving about 3.3 g of crude product, m.p. 33–36 °C. Sample (325.2 mg) of ester was purified with column chromatography, silica gel 60 (0.063–0.200 mm, 400–250 mesh), eluent: chloroform, yielding 319.6 mg of pure ester, m.p. of purified ester = 36–37 °C. Elemental analysis (accomplished by combustion analysis) for formula C_6_H_6_N_2_O_4_S of **L2** (calculated/found (%)): C 35.64/35.64, H 2.99/2.81, N 13.86/13.84, S 15.86/15.90.

^1^H NMR (600 MHz, acetone-d_6_): δ [ppm] 2.60 (s, 3H, CH_3_ in third position of isothiazole ring), 3.97 (s, 3H, CH_3_ of ester group). ^13^C NMR (150 MHz, acetone-d_6_): δ [ppm] 18.0 (CH_3_ in third position of isothiazole ring), 54.2 (CH_3_ of ester group), 144.9 (quaternary carbon of 4th position of isothiazole ring), 153.9 (quaternary carbon of 5th position of isothiazole ring), 158.8 (carbonyl carbon of ester group), 162.1 (quaternary carbon of 3th position of isothiazole ring). IR-ATR ν [cm^−1^]: 2961 (C-H stretching band of methyl group), 1734 (C=O bond strong stretching band of ester carbonyl), 1531 (C=C and C=N bond stretching bands of isothiazole ring), 1434, 1254 (C-O bond stretching of ester group). Visualizations of IR ^1^H-NMR, ^13^C-NMR and IR spectra of compound **L2** are in the Supplementary Materials (Figures S7, S8 and S26, respectively).

Analysis of mass spectra (ESI-MS) of methyl 3-methyl-4-nitro-5-isothiazolecarboxylate (**L2**): The calculated value of the parent peak mass [M] for the formula C_6_H_6_N_2_O_4_S is 202.004828 u and for the ionized form (C_6_H_6_N_2_O_4_S + H^+^) is 203.012104 u. The ESI-MS (positive ionization in methanol) of ester **L2** revealed, amongst others, the following peaks given as a ratio *m*/*z* [u/e] (relative intensity to base peak [%]): 224.994037 (100%) [M + Na^+^]^+^—quasi-molecular ion (base peak), 203.012113 (6.2%) [M + H^+^]^+^—quasi-molecular ion (parent) peak. Visualizations of ESI-MS spectra are in the Supplementary Materials (Figure S39).

### 3.2. Reactions of Nitroisothiazoles with Potassium Tetrachloroplatinate

#### 3.2.1. General Description of the Reactions of Nitroisothiazoles with K_2_PtCl_4_

A total of 2 mmol of the appropriate organic ligand was dissolved in the appropriate amount of the mixture of acetone and distilled water. Then, 415 mg (1 mmol) of K_2_PtCl_4_ in 10 mL of water was added to a solution of the appropriate ligand, and the whole mixture was stirred and left in a sealed reaction flask protected from light as long as the reaction was completed (i.e., from 5 days to about 9 months depending on ligand).

#### 3.2.2. Complexation with 3-Methyl-4-nitroisothiazole (L1) Resulting in *cis* and *trans*-Dichlorobis(3-methyl-4-nitroisothiazole)platinum(II) (**C1** and **C2**) (M_mol_ = 554.28908 g/mol)

Solutions of 288.3 mg (2 mmol) of 3-methyl-4-nitroisothiazole (**L1**) in the mixture of 10 mL of acetone and 10 mL of distilled water were mixed with a solution of 415 mg (1 mmol) of K_2_PtCl_4_ in 10 mL of distilled water. The flask containing the reaction mixture was corked and protected from light. The course of the reaction was checked by TLC. After 5 days, the resulting yellow precipitate was filtered off and dried in a vacuum. A total of 430 mg (yield 77.6%) of crude product was yielded, which appeared to be a mixture of two complexes with platinum (test with rubeanic acid on TLC chromatogram). The compounds were separated and purified using column chromatography with silica gel type 60 (pore size 60 Å, particle size 0.040–0.063 mm, i.e., 400–230 mesh SiO_2_). Initially, chloroform was used as eluent, then gradually acetone was added (CHCl_3_-Acetone 95:5 volume ratio—finally 8:2 volume ratio). First, a less polar fraction was collected that contained *trans* complex **C2**. Then, a more polar fraction that contained *cis* complex **C1** was collected. 270 mg of pale-yellow *cis* isomer **C1** from the more polar fraction was obtained (yield 48.7%). The melting point (m.p.), the slow decomposition, and darkening of *cis*-isomer C**1** start at about 165 °C, with complete decomposition occurring at ≥230 °C. From a less polar fraction, 140 mg (yield 25.3%) of the light-yellow *trans* complex **C2** was separated. The m.p. (decomposition) of *trans* isomer **C2** starts at about 220 °C. It is weakly soluble in acetone but a little better than the *cis* isomer **C1**. Both isomers are very slightly soluble in chloroform Elemental analysis for formula C_8_H_8_N_4_O_4_S_2_Cl_2_Pt of *cis*-complex **C1** (calculated/found (%)): C 17.33/17.35, H 1.46/1.30, N 10.11/10.06, S 11.57/11.59. Elemental analysis for formula C_8_H_8_N_4_O_4_S_2_Cl_2_Pt of the *trans* complex **C2** (calculated/found (%)): C 17.33/17.51, H 1.46/1.46, N 10.11/10.08, S 11.57/11.50.

Spectral analysis for the *cis* complex **C1**:

^1^H-NMR (600.13 MHz, acetone-d_6_): δ [ppm] 3.12 (s, 3H, CH_3_ in third position of isothiazole ring), 10.04 (s, 1H, proton in fifth position of isothiazole ring). Visualization of the ^1^H-NMR spectrum is in the Supplementary Materials (Figure S3).

^13^C NMR (150.92 MHz, acetone-d_6_): δ [ppm] 21.6 (carbon of CH_3_ in third position of isothiazole ring), 145.4 (quaternary carbon 4 of isothiazole ring), 154.3 (quaternary carbon 5 of isothiazole ring), 166.7 (quaternary carbon 3 of isothiazole ring). Visualization of the ^13^C-NMR spectrum is in the Supplementary Materials (Figure S4).

MIR-ATR ν [cm^−1^]: 3106 (νC-H of isothiazole ring), 3009, 2942, 2846 (νC-H bands of stretching vibrations of methyl group), 1550 (ν_s_O-N-O of nitro group), 1507 (νC=C, C=N), 1420 (bending vibration of CH_3_ group), 1345 (ν_as_O-N-O of nitro group), 1173 (bending vibration of C-H of isothiazole ring). Visualization of the MIR-ATR spectrum is in the Supplementary Materials (Figure S24).

Far-IR-ATR ν [cm^−1^]: 343 (ν_s_Cl-Pt), 337 (ν_as_Cl-Pt). Visualization of the Far IR-ATR spectrum is in the Supplementary Materials (Figure S31).

Analysis of mass spectra (ESI-MS): The calculated value of the parent peak mass [M] for the formula C_8_H_8_N_4_O_4_S_2_Cl_2_Pt is 552.901193 u. The ESI-MS (positive ionization in methanol) of **C1** revealed, amongst others, the following peaks given as a ratio *m*/*z* [u/e] (relative intensity to base peak [%]): 576.885432 (100%) [M + 1 + Na^+^]^+^—isotope peak of quasi-molecular ion (base peak), 575.887610 (70.9%) [M + Na^+^]^+^—quasi-molecular ion (parent) peak; the ESI-MS (negative ionization in methanol) of **C1** revealed, amongst others, the following peaks given as a ratio *m*/*z* [u/e] (relative intensity to base peak [%]): 552.912777 (100%) [M + 1-H^+^]^−^—isotope peak of quasi-molecular ion (base peak), 551.914782 (69.3%) [M-H^+^]^−^—quasi-molecular ion (parent) peak. Visualizations of the ESI-MS spectra are in the Supplementary Materials (Figures S36 and S37).

Spectral analysis for the *trans* complex **C2**:

^1^H NMR (600.13 MHz, acetone-d_6_): δ [ppm] 3.25 (s, 3H, CH_3_ in third position of isothiazole ring), 10.05 (s, 1H, proton in fifth position of isothiazole ring). Visualization of the ^1^H-NMR spectrum is in the Supplementary Materials (Figure S5).

^13^C NMR (150.92 MHz, acetone-d_6_): δ [ppm] 21.8 (carbon of CH_3_ in third position of isothiazole ring), 144.8 (quaternary carbon 4 of isothiazole ring), 154.6 (quaternary carbon 5 of isothiazole ring), 166.4 (quaternary carbon 3 of isothiazole ring). Visualization of the ^13^C-NMR spectrum is in the Supplementary Materials (Figure S6).

MIR-ATR ν [cm^−1^]: 3109 (νC-H stretching vibration of isothiazole ring), 3002, 2929, 2848 (νC-H bands of stretching vibrations of methyl group), 1550 (ν_s_O-N-O of nitro group), 1505, 1507 (νC=C, C=N), 1413 (bending vibration of CH_3_ group), 1345 (ν_as_O-N-O of nitro group), 1178 (bending vibration of C-H of isothiazole ring). Visualization of the MIR-ATR spectrum is in the Supplementary Materials (Figure S25).

Far-IR-ATR ν [cm^−1^]: 345 (ν_as_Cl-Pt). Visualization of the Far IR-ATR spectrum is in the Supplementary Materials (Figure S32).

Analysis of mass spectra (ESI-MS): The calculated value of the parent peak mass [M] for the formula C_8_H_8_N_4_O_4_S_2_Cl_2_Pt is 552.901193 u. The ESI-MS (negative ionization in methanol) of **C2** revealed, amongst others, the following peaks given as a ratio *m*/*z* [u/e] (relative intensity to base peak [%]): 552.907854 (57.69%) [M + 1-H^+^]^−^—isotope peak of quasi-molecular ion, 551.910015 (41.2%) [M-H^+^]^−^—quasi-molecular ion (parent) peak. Visualization of the ESI-MS spectrum is in the Supplementary Materials (Figure S38).

#### 3.2.3. Complexation with Methyl 3-Methyl-4-nitroisothiazole-5-carboxylate (**L2**) Resulting in *trans*-Dichlorobis(3-methyl-4-nitro-5-(methoxycarbonyl)isothiazole)platinum(II) (**C4**) (M_mol_ = 670.36124 g/mol) and *trans*-Dichloro-3-methyl-4-nitroisothiazole 3-Methyl-4-nitro-5-(methoxycarbonyl)isothiazole Platinum(II) (**C3**) (M_mol_ = 612.32516 g/mol)

Solution of 404.4 mg (2 mmol) of 3-methyl-4-nitro-5-isothiazolecarboxylate (**L2**) in 10 mL of acetone was mixed with a solution of 415 mg (1 mmol) of K_2_PtCl_4_ in 10 mL of water. The flask containing the reaction mixture was corked and protected from light. The course of the reaction was checked by TLC. After 21 days, the resulting yellow precipitate was filtered off and dried in a vacuum. A total of 400 mg of crude product was yielded, which appeared to be a mixture of two complexes with platinum (test with rubeanic acid on TLC chromatogram). The compounds were separated and purified using column chromatography with silica gel type 60 (pore size 60 Å, particle size 0.040–0.063 mm, i.e., 400–230 mesh SiO_2_). Initially, chloroform was used as eluent, then gradually acetone was added (initially CHCl_3_-Acetone 95:5 volume ratio—finally 9:1 volume ratio). Firstly, 70 mg of the starting ester **L2** was recovered. Then, the fraction was collected containing Pt-complex that appeared to be *trans* isomer **C4** with both ester ligands **L2**, and finally, the fraction containing Pt-complex that appeared to be *trans*-isomer **C3** with mixed ligands **L1** and **L2**. An amount of 80 mg of yellow *trans*-isomer **C3** from the more polar fraction was obtained (yield 11.9%). Both complexes **C3** and **C4** are slightly soluble in acetone but very poorly soluble in chloroform. The melting point, slow decomposition, and darkening of compound **C3** begins at a temperature of approximately 130 °C, with complete decomposition occurring at 195 °C. From a less polar fraction, 211 mg (yield 31.5%) of the light-yellow *trans* complex C**4** was separated. The m.p. (decomposition) of *trans* isomer **C4** starts at about 215 °C. Elemental analysis for formula C_12_H_12_N_4_O_8_S_2_Cl_2_Pt of the *trans* complex **C4** (calculated/found (%)): C 21.50/21.43, H 1.80/1.57, N 8.36/8.44, S 9.57/9.60.

Spectral analysis for the *trans* complex **C3**:

^1^H NMR (600.13 MHz, acetone-d_6_): δ [ppm] 3.12 (s, 3H, CH_3_ in third position of methyl 3-methyl-4-nitroisothiazole-5-carboxylate ligand (**L2**), 3.24 (s, 3H, CH_3_ in third position of 3-methyl-4-nitroisothiazole ligand (**L1**)), 4.02 (s, 3H, CH_3_ of ester group), 10.06 (s, 1H, proton in fifth position of 3-methyl-4-nitroisothiazole (**L1**)). Visualization of the ^1^H-NMR spectrum is in the Supplementary Materials (Figure S9).

^13^C NMR (150.92 MHz, acetone-d_6_): δ [ppm] 20.7, 21.8 (carbons of CH_3_ in third position of isothiazole ring), 55.0 (carbon of ester methyl group), 143.0, 144.9 (quaternary carbons 4 of isothiazole ring of both ligands **L1** and **L2**), 154.3, 154.7 (quaternary carbon 5 of isothiazole ring of both ligands), 157.8 (quaternary carbonyl carbon of isothiazole ring), 166.4, 166.5 (quaternary carbon 3 of isothiazole ring of both ligands). Visualization of the ^13^C-NMR spectrum is in the Supplementary Materials (Figure S10).

MIR-ATR ν [cm^−1^]: 3109 (νC-H stretching vibration of isothiazole ring), 2951, 2925, 2851 (νC-H bands of stretching vibrations of methyl group), 1751, 1728 (νC=O), 1554 (ν_s_O-N-O of nitro group), 1525, 1510 (νC=C, C=N, bands of stretching vibrations of isothiazole ring), 1436, 1417 (bending vibration of CH_3_ group), 1346 (ν_as_O-N-O of nitro group), 1288 (νC-O vibration of ester bond), 1176 (bending vibration of C-H of isothiazole ring). Visualization of the MIR-ATR spectrum is in the Supplementary Materials (Figure S27).

Far-IR-ATR ν [cm^−1^]: 347 (ν_as_Cl-Pt). Visualization of the Far IR-ATR spectrum is in the Supplementary Materials (Figure S33).

Analysis of mass spectra (ESI-MS): The calculated value of the parent peak mass [M] for the formula C_10_H_10_N_4_O_6_S_2_Cl_2_Pt is 610.906673 u. The ESI-MS (positive ionization in methanol) of **C3** revealed, amongst others, the following peaks given as a ratio *m*/*z* [u/e] (relative intensity to base peak [%]): 634.886749 (6.68%) [M + 1 + Na^+^]^+^—isotope peak of quasi-molecular ion, 633.888908 (4.60%) [M + Na^+^]^+^—quasi-molecular ion (parent) peak; the ESI-MS (negative ionization in methanol) of **C3** revealed, amongst others, the following peaks given as a ratio *m*/*z* [u/e] (relative intensity to base peak [%]):610.924642 (100%) [M + 1-H^+^]^−^—isotope peak of quasi-molecular ion (base peak), 609.926618 (66.53%) [M-H^+^]^−^—quasi-molecular ion (parent) peak. Visualizations of the ESI-MS spectra are in the Supplementary Materials (Figures S40 and S41).

Spectral analysis for the *trans* complex **C4**:

^1^H NMR (600.13 MHz, acetone-d_6_): δ [ppm] 3.12 (s, 3H, CH_3_ in third position of methyl 3-methyl-4-nitroisothiazole-5-carboxylate (**L2**)), 4.02 (s, 3H, CH_3_ of ester group). Visualization of the ^1^H-NMR spectrum is in the Supplementary Materials (Figure S11).

^13^C NMR (150.92 MHz, acetone-d_6_): δ [ppm] 21.8 (carbons of CH_3_ in third position of isothiazole ring), 55.0 (carbon of ester methyl group), 143.0 (quaternary carbon 4 of isothiazole ring), 155.2 (quaternary carbon 5 of isothiazole ring), 157.7 (quaternary carbonyl carbon of isothiazole ring), 166.6 (quaternary carbon 3 of isothiazole ring). Visualization of the ^13^C-NMR spectrum is in the Supplementary Materials (Figure S12).

MIR-ATR ν [cm^−1^]: 2966, 2926 (νC-H bands of stretching vibrations of methyl group), 1739 (νC=O), 1554 (ν_s_O-N-O of nitro group), 1539 (νC=C, C=N, bands of stretching vibrations of isothiazole ring), 1436 (bending vibration of CH_3_ group), 1346 (ν_as_O-N-O of nitro group), 1261 (νC-O vibration of ester bond). Visualization of the MIR-ATR spectrum is in the Supplementary Materials (Figure S28).

Far-IR-ATR ν [cm^−1^]: 354 (ν_as_Pt-Cl). Visualization of the Far IR-ATR spectrum is in the Supplementary Materials (Figure S34).

Analysis of mass spectra (ESI-MS) of **C4**: The calculated value of the parent peak mass for the formula C_12_H_12_N_4_O_8_S_2_Cl_2_Pt is 668.912152 u. The ESI-MS (positive ionization in methanol) of **C4** revealed, amongst others, the following peaks given as a ratio *m*/*z* [u/e] (relative intensity to base peak [%]): 692.883204 (100%) [M + 1 + Na^+^]^+^—isotope peak of quasi-molecular ion (base peak), 691.885349 (67.90%) [M + Na^+^]^+^—quasi-molecular ion (parent) peak; the ESI-MS (negative ionization in methanol) of **C4** revealed, amongst others, the following peaks given as a ratio *m*/*z* [u/e] (relative intensity to base peak [%]): 668.911212 (39.33%) [M + 1-H^+^]^−^—isotope peak of quasi-molecular ion, 667.911813 (27.15%) [M-H^+^]^−^—quasi-molecular ion (parent) peak, 610.903236 (65.05%) [M-CO_2_Me + 1]^−^—isotope peak of fragmentation ion, 609.905014 (47.91%) [M-CO_2_Me]^−^—fragmentation ion peak. Visualizations of the ESI-MS spectra are in the Supplementary Materials (Figures S42 and S43).

#### 3.2.4. Complexation with 3-Methyl-4-nitroisothiazole-5-carbonitrile (**L3**) Resulting in *cis*-Dichlorobis(3-methyl-4-nitroisothiazole-5-carboxamide)platinum(II) (**C5**) (M_mol_ = 640.33864 g/mol)

Solution of 338.3 mg (2 mmol) of 3-methyl-4-nitroisothiazole-5-carbonitrile (**L3**) in 20 mL of acetone was mixed with a solution of 415 mg (1 mmol) of K_2_PtCl_4_ in 10 mL of water. The flask containing the reaction mixture was corked and protected from light. The course of the reaction was checked by TLC. After about 9 months, the resulting pale-yellow precipitate was filtered and dried in a vacuum, giving 110 mg of crude product. TLC of the crude product showed, through reaction with rubeanic acid, that it is a contaminated platinum compound that was purified with flash chromatography with silica gel type 60 (pore size 60 Å, particle size < 0.063 mm, i.e., >230 mesh SiO_2_) column using chloroform–acetonitrile mixture eluent gradientally changing from volume ratio 7:3 to 4:6. The mixture was initially applied as a chloroform solution and developed with chloroform, then the polarity of the eluent was increased by the addition of acetonitrile. As a result, 98.2 mg of pure **C5** complex was obtained. Additionally, 8 mg of known, previously described 3-methyl-4-nitroisothiazole-5-carboxamide (**L4**) was also isolated as a hydrolysis product of nitrile **L3**, m.p. 164–166 °C, and is consistent with the previously given [30,37]. IR, ^1^H, and ^13^C NMR spectra of nitroamide **L4** are consistent with those described earlier in our previous publication [30], and their visualization (see Supplementary Materials, Figures S17–S20 for NMR and S30 for IR) as well as ^1^H-^13^C 2D HMBC (Heteronuclear Multiple Bond Correlation) of nitroamide **L4** spectrum is presented in the Supporting Materials (Figure S21). The presence of a platinum compound identical to the one filtered out was also detected in the filter. The filtrate was concentrated to dryness by vacuum evaporation of the solvents, and the dry residue (about a highly contaminated mixture) was purified by flash chromatography in the same manner as described above, with the difference being that the eluent was a fixed mixture of chloroform and acetonitrile in a volume ratio of 9:1. A total of 24 mg of starting nitrile ligand **L3** was recovered, m.p. = 107–108 °C [30], and about 56.9 mg of nitroamide **L4** was also isolated. An additional 73.9 mg of complex **C5** was obtained from the filtrate. Summarizing the purification process of both precipitate and the filtrate, 24 mg of unreacted starting nitrile **L3** was recovered, 64.9 mg of nitroamide **L4** [30], and 172.1 mg (yield 26.9%) of light-yellow complex **C5** was obtained, which, after analysis, turned out to be a platinum complex with nitroamide **L4**. Complex **C5** is weakly soluble in acetone and slightly better in acetonitrile. The m.p. (decomposition) of complex **C5** starts at about 260 °C. Elemental analysis for formula C_10_H_10_N_6_O_6_S_2_Cl_2_Pt of compound **C5** (calculated/found (%)): C 18.76/18.82, H 1.57/1.40, N 13.12/13.15, S 10.02/9.98.

Spectral analyses for the *cis* complex **C5**:

^1^H NMR (600.13 MHz, acetone-d_6_): δ [ppm] 3.19 (s, 3H, CH_3_ in third position of isothiazole ring), 7.85, and 8.14 (2H, NH_2_ of amide group, the higher shift value for one of the protons of the amide group results from the formation of a strong intramolecular hydrogen bond with the oxygen atom of the neighboring nitro group). Visualization of the ^1^H-NMR spectrum is in the Supplementary Materials (Figure S13).

^13^C NMR (150.92 MHz, acetone-d_6_): δ [ppm] 21.9 (carbons of CH_3_ in third position of isothiazole ring), 141.0 (quaternary carbons 4 of isothiazole ring), 157.9 (quaternary carbon 5 of isothiazole ring), 163.3 (quaternary carbonyl carbon of isothiazole ring), 166.8 (quaternary carbon 3 of isothiazole ring). Visualization of the ^13^C-NMR spectrum is in the Supplementary Materials (Figure S14).

^1^H-^13^C 2D HMBC NMR (Phase cycled magnitude-mode 2D HMBC using low-pass J-filter (hmbclpndqf | HMBCLPND), acetone-d6, F2 = 600.13 MHz, F1 = 150.92 MHz, acetone-d6, 32 scans, relaxation delay = 1.5 s, aqt = 0.2621 s, T = 298 K) spectrum shows strong long range coupling ^2^J_CH_ through two bonds between methyl protons (3.19 ppm) and quaternary carbon 3 (166.5 ppm) and little less intensive signal that points the coupling ^3^J_HC_ through three bonds between methyl protons (3.19 ppm) and quaternary carbon 4 (140.8 ppm). Visualization of the HMBC spectrum is in the Supplementary Materials (Figures S15 and S16).

MIR-ATR ν [cm^−1^]: 3456–2966 (νN-H, stretching vibrations of NH_2_ amide group), 2933, 2894, 2854 (νC-H bands of stretching vibrations of methyl group), 1685, 1675 (νC=O), 1595 (bending vibration of amide NH_2_ group), 1550 (ν_s_O-N-O of nitro group), 1515–1503 (νC=C, C=N, bands of stretching vibrations of isothiazole ring), 1424 (bending vibration of CH_3_ group), 1347 (ν_as_O-N-O of nitro group). Visualization of the MIR-ATR spectrum is in the Supplementary Materials (Figure S29).

Far-IR-ATR ν [cm^−1^]: 344 (ν_s_Cl-Pt), 339 (ν_as_Cl-Pt). Visualization of the Far IR-ATR spectrum is in the Supplementary Materials (Figure S35).

Analysis of mass spectra (ESI-MS) of **C5**: The calculated value of the parent peak mass for the formula C_10_H_10_N_6_O_6_S_2_Cl_2_Pt is 638.912821 u. The ESI-MS (positive ionization in methanol) of **C5** revealed, amongst others, the following peaks given as a ratio *m*/*z* [u/e] (relative intensity to base peak [%]): 662.900409 (98.93%) [M + 1 + Na^+^]^+^—isotope peak of quasi-molecular ion, 661.901957 (70.26%) [M + Na^+^]^+^—quasi-molecular ion (parent) peak; the ESI-MS (negative ionization in methanol) of **C5** revealed, amongst others, the following peaks given as a ratio *m*/*z* [u/e] (relative intensity to base peak [%]): 638.932999 (24.70%) [M + 1-H^+^]^−^—isotope peak of quasi-molecular ion, 637.935003 (17.55%) [M-H^+^]^−^—quasi-molecular ion (parent) peak, 595.925283 (100%) [M-CONH_2_ + 1]^−^—isotope peak of fragmentation ion (base peak), 594.927159 (70.31%) [M-CONH_2_]^−^—fragmentation ion peak. Visualizations of the ESI-MS spectra are in the Supplementary Materials (Figures S44 and S45).

### 3.3. Single Crystal X-Ray Structure Determination of Methyl 3-Methyl-4-nitro-5-isothiazolecarboxylate (**L2**) and Complex **C4**

In order to obtain a crystal of 3-methyl-4-nitro-5-isothiazolocarboxylate methyl (**L2**) that would be suitable for XRD measurement, the solvent was slowly evaporated from its acetone solution. In contrast, a suitable crystal of the *trans*-complex **C4** was formed as a result of slow evaporation of the solvents from the solution of a mixture of carbon tetrachloride (CCl_4_) and acetone. X-ray data were collected on an Xcalibur Ruby, Gemini Ultra diffractometer with Mo-Kα radiation (λ = 0.71073 Å) at 100(2) K, using an Oxford Cryosystems cooler. Data collection, refinement, reduction, and analysis were carried out with the CRYSALIS^PRO^ (Rigaku Oxford Diffraction Ltd., Abingdon, UK, 2020). An analytical absorption correction was applied with the use of CRYSALISPRO RED [Computer Programs: CrysAlis PRO, Rigaku OD, 2015; Rigaku Oxford Diffraction Ltd.: Abingdon, UK, 2020]. The crystal structures were solved with Intrinsic Phasing using the SHELXT program and refined using SHELXL (2015 release) [38,39] with anisotropic thermal parameters for non-H atoms. In the final refinement, all H atoms were treated as riding atoms in geometrically optimized positions. Figure 2 and Figure 5 were produced using the DIAMOND program [40]. CCDC numbers of compound **C4** (2443665) and **L2** (2443883) contain the supplementary crystallographic data for this paper. These data are provided free of charge by The Cambridge Crystallographic Data Centre via www.ccdc.cam.ac.uk/data_request/cif. (accessed on 17 December 2025). The crystal data and details of data collection and refinement procedures are collected in Tables S1–S11 in the Supplementary Materials.

### 3.4. Computational Details

A full geometry optimization and the thermochemistry calculations for molecules taking part in the considered reactions and transformations have been performed on the basis of ab initio quantum mechanical DFT (density functional theory) method using the various combination of B3LYP hybrid density functional [41,42] with the extended Pople 6-311++G(df,pd), Dunning aug-cc-pVTZ and Jorge DZP and Ahlrichs-Karlsruhe def2-TZVPD (Valence triple-zeta polarization with diffuse functions) basis sets for all elements of ligands. DZP (all-electron), Pt-mDZP (all-electron), dhf-qzvpp (ECP—Effective Core Potential), and LANL2TZf (ECP-Effective Core Potential) basis sets [26,27] were used for platinum. The molecules of the compounds were treated as single isolated molecules in a vacuum environment. However, for simulation of the solvent environment, a Solvation Model based on the solute electron density (SMD) model of Self-Consistent Reaction Field (SCRF) [25] was used with the Gaussian 16 default set of parameters for acetone and water solvents. However, for simulation of water–acetone (in ratio 2:1 vol.) solvent mixture environment, the following relative electric permittivity values were used: Eps = 72.079 (low frequency electric field, static) and Epsinf = 1.785278 (high frequency electric field), calculated on the base of mole fraction according to the formula ${\varepsilon_{mix}=\varepsilon_{water}\phi_{water}+\;\varepsilon }_{aceton}\phi_{acetone}$, where $\varepsilon_{mix}$ is electric permittivity of solvent mixture, $\varepsilon_{water}$ and $\varepsilon_{acetone}$ are electric permittivities of pure water and acetone, and φ is mole fraction of appropriate solvent in the mixture [43]. The other applied parameters were like in the acetone case. All the calculations were computed using the Gaussian 2016 revision C.02 software [24]. The absence of imaginary wavenumbers in the resulted frequency calculations is the confirmation that the final structure and presented results correspond to a minimum on the potential energy surface. The thermochemistry calculations were performed for standard conditions, i.e., T = 298.15 K and *p* = 1 atm. The Gibbs energies (ΔG_r_) of the complexation reactions were calculated as the difference between the sums of the Gibbs energies of all the products (G_products_) and all the substrates (G_substrates_). Equilibrium constant of *trans–cis* isomerization (K_iso_) was calculated based on the relation with estimated Gibbs energy of the *trans*-to-*cis* isomer conversion (ΔG_isom._) through the following expression: ΔG_isom._ = −RT·lnK, where R is the molar gas constant (1.98720425864083 × 10^−3^ kcal·K^−1^·mol^−1^) and T is the absolute temperature (298.15 K). Details on how to perform thermochemistry calculations using Gaussian software can be found in the following papers [29,44].

The designed TS structures (cartesian coordinates can be found in Tables S12 and S13 in the Supplementary Materials) were optimized and validated by confirming the presence of a single imaginary wave number through oscillatory calculations (freq) and then checked to see if they were a saddle point connecting substrates and products through intrinsic reaction coordinate (IRC) calculations [29].

### 3.5. Reaction with L-Glutathione

The experiment was conducted in a similar manner to that described in previous publications [17,18]. First, 30 μL of 60 mM solution of the appropriate complex (*cis* **C1**, *trans* **C2,** *trans* **C3** and *trans* **C4**) in purified and anhydrous distilled dioxane was mixed with 3 mL of a 2 mM solution of reduced L-glutathione (GSH) in an argon-saturated medium containing 10 mmol/L NaCl and 10 mmol/L Tris x HCl buffer, pH 7.4, to achieve a final concentration of 6 µM of the tested complexes. The measurement was carried out at 37 °C. The progress of the reaction was monitored by UV absorption spectrometry at a wavelength of 260 nm. Dioxane was used to dissolve the tested Pt complexes due to the lack of UV absorption of this solvent at the used wavelength. The GSH solution was saturated with argon to remove oxygen and thus prevent oxidation of GSH with the formation of S-S bonds, which would interfere with the measurement results.

### 3.6. Cell Culture Used for Testing

Three solid tumor cell lines were used in this study: MCF-7 (breast adenocarcinoma), ES-2 (ovarian adenocarcinoma), and A549 (lung adenocarcinoma). The BALB/3T3 (mouse embryonic fibroblast) cell line was used as a comparative nonpathological tissue. All cell lines were purchased from the American Type Culture Collection (ATCC, Rockville, MD, USA). The preparation of cell cultures for cytotoxicity experiments is described in detail in the following article [17].

### 3.7. Preparation of Stock Solutions of Tested Compounds to In Vitro Studies

The initial solutions for subsequent dilutions in biological medium were prepared in a similar manner as described previously [17]. The complexes **C1**–**C4** and reference cisplatin were dissolved in acetone. The complex **C5** was dissolved in the dimethylacetamide (DMA). All the initial solutions were prepared ex tempore and then were added to the culture medium 1:9 (*v/v*) to obtain a stock solution of 1 mg/mL.

### 3.8. In Vitro Cytotoxicity Test Through Sulforhodamine B Assay

The test was prepared and performed in the same manner as detailed in the following cited works [17,45,46]. The results are shown in Table 4.

### 3.9. Statistical Analysis

Statistical analysis was performed with the use of software such as Statistica (data analysis software system), version 13.3 (2017, TIBCO Software Inc., Palo Alto, CA, USA), and PTC Mathcad Express Prime 6.0.0.0. (Copyright 2019, PTC Inc., Boston, MA, USA). The normal distribution of the data within the groups was verified with the Shapiro–Wilk test and the Kolmogorov–Smirnov (K-S) test with Lilliefors correction. The homogeneity of variances was checked with Levene’s and Brown–Forsythe tests. The statistical significance of the differences in the mean IC_50_ values of the tested compounds in relation to the mean IC_50_ values of the reference drug was tested with Dunnett’s post hoc test (2-sided). In addition, the statistical significance of differences in the mean IC_50_ values of each complex for cancerous cell lines and healthy reference cells, and between the mean IC_50_ values for normoxic and hypoxic conditions, was also tested with Dunnett’s post hoc test (2-sided) and with a T-test for independent samples. The confidence level (limit) was set as 95%, and the threshold for significance level was set at α = 0.05; thus, the differences between the means with a *p*-value (calculated probability value) < 0.05 (i.e., at probability level > 95%) were considered statistically significant.

## 4. Conclusions

A review of the literature revealed that no research results have been published on the nature of platinum complex formation with isothiazoles. No unambiguous confirmation of the complexation of isothiazoles with platinum via nitrogen rather than sulfur has been presented to date. Our studies on the complexation of platinum(II) ion by some 4-nitroisothiazoles indicate that the experimental results (by XRD measurement) finally confirm the theoretical predictions that the complexation of nitroisothiazoles with platinum (II) ion occurs via the nitrogen of the isothiazole ring. Previously, in 1992, van Beusichem and Farrell proved, using the XRD method, that platinum (II) forms complexes with isomeric thiazole via the nitrogen atom [47]. It should be noted that thiazole and isothiazole, despite being isomers, exhibit significantly different chemical properties, mainly due to the direct connection of two heteroatoms in the isothiazole ring, which results in a marked decrease in the basicity of the ring azomethine nitrogen.

A pair of isomers (*cis*-complex **C1** and *trans*-complex **C2**) was obtained only with the simplest ligand used, which was unsubstituted in the fifth position, i.e., 3-methyl-4-nitroisothiazole (**L1**). The theoretically estimated energies of formation for the *cis* **C1** and *trans* **C2** isomeric complexes indicate that the *trans* isomer **C2** should be more thermodynamically stable than the *cis* **C1**. However, the reaction of potassium chloroplatinate with the ligand **L1** predominantly yields the *cis* isomer **C1**. The calculated activation energy (transition state energy TS_cis_) for the formation of the *cis* isomer **C1** is lower than that (TS_trans_) for the *trans* isomer **C2**, which explains why the *cis* isomer **C1** forms predominantly during the reaction, despite its lower thermodynamic stability. This indicates that the reaction is kinetically controlled. In the reaction between potassium tetrachloroplatinate and ester **L2**, two *trans* complexes, **C3** and **C4**, are obtained. One of these (complex **C3**) contains a ligand from which the ester group is removed through hydrolysis and decarboxylation. In contrast, in the reaction with the nitrile ligand **L3**, instead of obtaining a complex with both nitrile ligands, as expected, a complex with amide ligands (*cis* complex **C5**) was formed as a result of hydrolysis of the nitrile to an amide group. Additionally, studies on the complexation of platinum(II) ions with some 4-nitroisothiazoles indicate that this process is more facile than with 4-nitroisoxazoles. In the case of 4-nitroisoxazoles, only two isomeric complexes were obtained, and only one nitroisoxazole derivative (out of four tested) formed a pair of complex isomers, due to the lower stability of the isoxazole ring, which decomposed in most cases during complexation [18]. However, in the case of isothiazole nitronitrile **L3**, the process is lengthy and involves decomposition of the nitrile group to an amide group. Furthermore, the synthesis of nitroisothiazole ligands is tedious and time-consuming, which prolongs the entire process.

Most of all, the tested nitroisothiazole complexes (except **C5**) have shown anticancer activity similar to the reference drug cisplatin. In the case of the MCF7 (breast) cancer cell line, three complexes **C1**–**C3** have revealed stronger activity than cisplatin. However, they are toxic to both rapidly dividing, healthy cells and cancer cells, just like the reference drug cisplatin.

Comparing the anticancer cytotoxic activity of platinum complexes with nitroisothiazoles with previously tested complexes with nitropyrazoles and nitroisoxazoles [17,18], it can be concluded that the best anticancer activity against the tested cancer cell lines was exhibited by complexes with nitroisoxazoles, which were significantly more cytotoxic against all the cancer cell lines tested than the reference drug cisplatin. At the same time, however, complexes with nitroisoxazoles exhibited high cytotoxicity to healthy rapidly dividing cells, which was similar or stronger, depending on the compound, compared to the reference cisplatin. Generally, weaker anticancer activity was shown by complexes with nitropyrazoles and by the tested complexes with nitroisothiazoles, albeit whose anticancer activity was at the level of cisplatin activity. However, it should be noted that complexes with nitropyrazoles were the least cytotoxic to healthy, fast-dividing cells and therefore had the best selectivity indices (SIs) among the three groups of complexes. It should also be noted that complexes with nitropyrazoles were also significantly less cytotoxic to healthy cells than the reference drug cisplatin. In contrast, complexes with nitroisothiazoles were equally or, as particularly evident in the case of the *trans* complex **C2** (which was strongly active against all the cancer lines tested), more toxic to healthy, fast-dividing cells than to some cancer cell lines. However, none of the complexes with nitroisothiazoles proved to be more toxic to healthy, fast-dividing cells than cisplatin (cytotoxicity was at the same level), which distinguishes them from complexes with nitroisoxazoles, as said above. In addition, in a group of nitroisothiazole complexes, no major differences in anticancer activity were observed between normoxic and hypoxic conditions, except for the A549 cell line, where all tested complexes, except *trans* **C2**, proved to be inactive in hypoxic conditions. In turn, in a group of nitropyrazole complexes, most of the tested complexes appeared to be inactive or less active in hypoxic than in normoxic conditions. Furthermore, in a group of nitroisoxazole complexes, the compounds showed activity in both conditions and, depending on the cancer line, comparable or weaker activity in hypoxia.

Previous studies have shown that the susceptibility of cancer tissues to chemotherapy using platinum drugs depends on their ability to metabolize and excrete the drug from the cells [48]. These processes are related to the ability of the chemotherapeutic platinum agent to form Pt(II) complexes with endogenous thiols and proteins rich in sulfur-containing moieties, which may result in its diminished anticancer activity or deactivation.

Therefore, we investigated the reactivity of the most active anticancer complexes **C1**, **C2**, **C3**, and **C4** towards GSH. The results show that two *trans* complexes, **C3** and **C4**, react with GSH very quickly, 12 and 24 times faster than very reactive transplatin (see Table 5). Since the high reactivity of transplatin towards sulfur-containing molecular scavengers is considered one of the reasons for its pharmacological inactivity [49], the exceptionally short half-times of compounds **C3** and **C4** in the glutathione environment basically indicate their uselessness as potential antitumor agents. In turn, it is astonishing that removing the ester groups from the fifth position of the isothiazole ring causes the pair of isomeric complexes, i.e., *cis*-complex **C1** and *trans*-complex **C2**, to react with GSH much more slowly than both complexes **C3** and **C4**, but approximately three and two times faster than cisplatin, respectively (see Table 5). It is surprising that the *trans*-complex **C2** reacts with glutathione about twice as slowly as the isomeric *cis* **C1**, contrary to our previous studies showing that *trans* isomers are usually more reactive towards GSH than their *cis* congeners [17,18]. However, cases of *cis* isomers being more reactive to GSH than *trans* isomers have also been reported [50,51].

Compared to the previously studied *cis*-complexes of nitropyrazoles (e.g., with *cis*-complex with 1-methyl-4-nitropyrazole) and nitroisoxazoles (e.g., *cis*-complex with 3,5-dimethyl-4-nitroisoxazole) [17,18], the half-life of nitroisothiazole complexes in GSH solution is the shortest, nitroisoxazole complexes react more slowly, and nitropyrazole complexes are the least susceptible to this reaction (see Table 5). *cis*-complex with 1-methyl-4-nitropyrazole (*cis*-Pt(NO2Pyraz)_2_Cl_2_) [17] appeared to be less reactive towards GHS than cisplatin and oxaliplatin. *Cis*-complex with 3,5-dimethyl-4–nitroisoxazole (*cis*-Pt(NO2Isoxazol)_2_Cl_2_) [18] reacted with glutathione at a rate comparable to that of oxaliplatin but faster than cisplatin. However, all tested complexes with nitroisothiazole reacted faster than platinum drugs, and the most in vitro anticancer active, the complex **C2**, reacted about 1.5 times faster than oxaliplatin and 2 times faster than cisplatin (Table 5). For the above reasons, platinum complexes with nitroisothiazoles are not good candidates for potential drugs.

## Figures and Tables

**Table 1:** Theoretically predicted differences between the formation Gibbs energies (ΔΔ_f_G) of N-Pt bonded complexes and S-Pt bonded complexes. Calculations were performed for standard conditions (T = 298.15 K, *p* = 1 atm) with DFT (density functional theory) method using various combinations of functionals and basis sets. SMD (Solvation Model Density) model of SCRF (Self-Consistent Reaction Field) [25] for solvent (water–acetone (2:1 vol.) mixture parametrization for simulation of reaction environment) effect was used.

DFT Method Functional/Basis Sets	Difference Between Formation Gibbs Energies of *cis*-Complexes N-Pt (C1) and S-Pt (C1S) ΔΔ_f_G [kcal/mol]		Differences Between Formation Gibbs Energies of *trans*-Complexes N-Pt (C2) and S-Pt (C2S) ΔΔ_f_G [kcal/mol]	
	Environment			
	Vacuum	Water Acetone Mixture	Vacuum	Water Acetone Mixture
B3LYP/DZP *	−34.5	−41.1	−40.7	−44.0
B3LYP/aug-cc-pVTZ/DZP *^#^	−32.4	−40.1	−42.4	−45.0
B3LYP-GD3 ^‡^/aug-cc-pVTZ/DZP *^#^	−33.9	−40.7	−44.1	−47.4
B3LYP/aug-cc-pVTZ/Pt-mDZP *^#^	−30.0	−36.3	−39.3	−43.8
mPW1PW91/6-311++G(df,pd)/dhf-qzvpp *^#^	−23.4	−30.4	−29.9	−33.4
PBE1PBE/6-311++G(df,pd)/LANL2TZf *^#^	−26.9	−32.9	−32.5	−36.2
B3LYP/6-311++G(df,pd)/DZP *^#^	−41.8	−48.2	−46.5	−47.5
mPW1PW91/Def2TZVPD */dhf-qzvpp *^#^	−19.4	−25.9	−25.4	−29.1
PBE1PBE/Def2TZVPD */LANL2TZf *^#^	−21.2	−28.0	−26.6	−30.8
B3LYP/Def2TZVPD */DZP *^#^	−31.0	−38.0	−36.7	−40.0

* Basis set taken from EMSL/PNNL Basis Set Exchange [26,27]. ^#^ Basis set applied only for platinum atom. ^‡^ Empirical Dispersion GD3 correction was applied.

**Table 2:** Theoretically predicted the Gibbs energy of the *trans*-to-*cis* isomer conversion for *cis*-complex **C1** and *trans*-complex **C2** (Δ_f_G_cis_ − Δ_f_G_trans_ = ΔG_isom._). The calculations were performed for standard conditions (T = 298.15 K, *p* = 1 atm) with DFT (density functional theory) at B3LYP/aug-cc-pVTZ/DZP theory level. All-electron DZP basis set was applied for platinum atom and aug-cc-pVTZ for atoms of ligands. SMD (Solvation Model Density) model of SCRF (Self-Consistent Reaction Field) was used for simulation of solvent effect.

Environment	The Gibbs Energy of the *trans*-to-*cis* Isomer Conversion (C2 Versus C1) ΔG_isom._ [kcal/mol]	Equilibrium Constant of *trans*-*cis* Isomerization K_isom._
Vacuum	7.05	1.490 × 10^5^
Acetone	2.59	7.938 × 10
Water–Acetone (2:1 vol.)	1.75	1.913 × 10
Water	0.18	1.344

**Table 3:** TLC (Thin Liquid Chromatography) R_f_ (retardation factor) as a measure of the relative lipophilicity of the tested complexes **C1**–**C5**. TLC Al foils with fluorescent indicator 254 nm were used. The detection of compounds on chromatograms was carried out with a 0.5% acetone solution of rubeanic acid (dithiooxamide). Visualization of TLC chromatograms can be found in Supplementary Materials (Figures S48 and S49).

Compound	R_f_		
	Eluent: Chlorofom:Acetone		
	Ratio 9:1 Vol.	Ratio 9:7 Vol.	Ratio 1:1 Vol.
**C1**	0.054	0.442	0.726
**C2**	0.355	0.756	0.833
**C3**	0.645	0.814	0.845
**C4**	0.828	0.849	0.863
**C5**	0.005	0.215	0.655

**Table 4:** Cytotoxic activity of platinum complexes of 4-nitroisothiazoles **C1**–**C5** and reference cisplatin in normoxic and hypoxic conditions against some cancer and normal cell lines was determined in the SRB (sulforhodamine B) viability assay. Data are given as inhibitory concentration IC_50_ that causes a 50% reduction in the cell viability, IC_50_ ± SD [µM].

Compound	Cancer Cells						Normal Cells
	MCF-7 (Breast)		ES-2 (Ovarian)		A549 (Lung)		BALB/3T3
	Normoxia	Hypoxia	Normoxia	Hypoxia	Normoxia	Hypoxia	Normoxia
**C1**(*cis*)	4.59 ± 1.57 **	8.45 ± 2.25	7.29 ± 1.42	6.37 ± 1.69	31.41 ± 18.76	inactive	7.31 ± 0.82
**C2**(*trans*)	**6.16 ± 0.78** **	9.07 ± 5.07	2.14 ± 2.21	1.10 ± 0.34	**6.34 ± 1.47**	**6.52 ± 2.28**	1.14 ± 0.28
**C3**(*trans*)	5.25 ± 1.01 **	7.13 ± 1.93	3.90 ± 2.68	4.02 ± 2.47	5.74 ± 0.65	inactive	2.94 ±3.06
**C4**(*trans*)	6.94 ± 2.25	7.68 ± 4.45	3.89 ± 1.62	5.44 ± 1.64	**40.30 ± 19.93** *	inactive	5.99 ± 1.28
**C5**(*cis*)	57.57 ± 7.69 *	67.34 ± 28.34 *	62.48 ± 22.70 *	50.10 ± 7.97 *	66.20 ± 1.15 *	inactive	64.16 ± 15.83 *
**Cisplatin**	14.42 ± 1.40	9.74 ± 5.84	9.85 ± 4.68	6.28 ± 2.19	12.49 ± 1.64	14.75 ± 1.93	8.10 ± 5.61

** Cytotoxicity is statistically significantly higher than cisplatin; * statistically significantly less cytotoxic than cisplatin. The values marked in bold font show a statistically significant difference between cytotoxic activity against cancer cells and healthy ones. The confidence level (limit) was set as 95%, and the threshold for significance level was set at α = 0.05; hence, the differences between the means with a *p*-value (calculated probability value) < 0.05 (i.e., a probability level > 95%) were considered statistically significant.

**Table 5:** Comparison of half-times of reaction of chosen platinum drugs and tested platinum complexes with glutathione (GSH), arranged from slowest to fastest reacting with glutathione.

Compound/Drug	Half-Time, *t*_1/2_
Carboplatin	16.7–32.7 h [35]
*cis*-Pt(NO2Pyraz)_2_Cl_2_ ^‡^ [17]	83 min [17]
Cisplatin	60 min [35], 66 min [36]
Oxaliplatin	44 min [35]
*cis*-Pt(NO2Isoxazol)_2_Cl_2_ ^#^ [18]	42 min [18]
*trans*-complex **C2**	29 min
*trans*-Pt(NO2Isoxazol)_2_Cl_2_ ^#^ [18]	18 min [18]
*cis*-complex **C1**	16 min
*trans*-Pt(NO2Pyraz)_2_Cl_2_ ^‡^ [17]	13 min [17]
Transplatin	4 min [36]
*trans*-complex **C3**	20 s
*trans*-complex **C4**	10 s

^‡^ NO2Pyraz = 1-methyl-4-nitropyrazole; ^#^ NO2Isoxazol = 3,5-dimethyl-4–nitroisoxazole.

## Data Availability

The original contributions presented in this study are included in the article/Supplementary Material. Further inquiries can be directed to the corresponding authors.

## Supplementary Materials

The following supporting information can be downloaded at: https://www.mdpi.com/article/10.3390/molecules31010034/s1, including the detailed analysis of ESI-MS spectra, visualizations of IR and NMR spectra, and other additional information associated with this paper. CCDC reference numbers for compound **L2** and **C4** are: CCDC2443883 and CCDC2443665, respectively (Supplementary Data available from CCDC, 12 Union Road, Cambridge CB2, 1EZ, UK on request). These data can be obtained free of charge via www.ccdc.cam.ac.uk/structures/—accessed on 17 December 2025 (or from the Cambridge Crystallographic Data Centre, 12, Union Road, Cambridge CB2 1EZ, UK; fax: +44-1223-336033) or e-mail: deposit@ccdc.cam.ac.uk.
