# Supplementary material for: Studies on the Complexation of Platinum(II) by Some 4-Nitroisothiazoles and the Cytotoxic Activity of the Resulting Complexes
Source: Molecules. 2025 Dec 22;31(1):34. doi: 10.3390/molecules31010034 (PMC12786457; doi:10.3390/molecules31010034)
Supplement: Supplementary file 1 [file molecules-31-00034-s001.zip › molecules-3987912-supplementary.pdf]

SUPPLEMENTARY MATERIAL

Studies on the Complexation of Platinum(II) by Some 4-Nitroisothiazoles and the Cytotoxic Activity of the Resulting Complexes

Andrzej Regiec [1*], Joanna Wietrzyk [2], Magdalena Milczarek [2], Andrzej Kochel [3] and Henryk Mastalarz [1*],

[1] Department of Organic Chemistry and Drug Technology, Faculty of Pharmacy, Wrocław Medical University, 211A Borowska Street, 50-556 Wrocław, Poland

[2] Hirszfeld Institute of Immunology and Experimental Therapy Polish Academy of Sciences, 12 Rudolf Weigl Street, 53-114 Wrocław, Poland

[3] Faculty of Chemistry, Wrocław University, 14F Joliot-Curie Street, 50-383 Wrocław, Poland

Table of Contents

NMR spectra of ligands **L1-L5** and complexes **C1-C5 (Figures S1-S23)** pp **2-24**
MIR spectra of ligands **L2**, **L4** and complexes **C1-C5 (Figures S24-S30)** pp **25-31**
Far IR spectra of complexes **C1-C5 (Figures S31-S35)** pp **32-36**
MS-ESI spectra of ligand **L2** and complexes **C1-C5 (Figures S36-S45)** pp **37-46**
UV spectra of complexes **C1** and **C2 (Figures S46-S47)** p. **47**
Crystal data and structure refinement for compound **L2** (**Table S1**) p. **48**
Atomic coordinates and equivalent isotropic displacement parameters for**L2 (Table S2)** p. **49**
Selected bond lengths and angles for ligand **L2 (Table S3)** p. **50**
Anisotropic displacement parameters for ligand **L2** (**Table S4**) p. **51**
Hydrogen coordinates and isotropic displacement parameters for compound **L2** (**Table S5**) p. **51**
Torsion angles for compound **L2** (**Table S6**) p. **52**
Crystal data and structure refinement for *trans*-complex **C4** (**Table S7**) p. **53**
Atomic coordinates and equivalent isotropic displacement parameters for **C4 (Table S8)** p. **54**
Selected Bond lengths and angles for *trans*-complex **C4 (Table S9)** pp **55-56**
Anisotropic displacement parameters for *trans*-complex **C4 (Table S10)** p. **56**
Hydrogen coordinates and isotropic displacement parameters for *trans*-complex **C4 (Table S11)** p. **57**
Cartesian coordinates of TS_{cis} (**Table S12**) p. **58**
Cartesian coordinates of TS_{trans} (**Table S13**) p. **59**
TLC chromatograms of **C1-C5** (**Figures S48** and **S49**)

*Corresponding author, e-mail: Andrzej Regiec (andrzej.regiec@umw.edu.pl) and Henryk Mastalarz (henryk.mastalarz@umw.edu.pl)

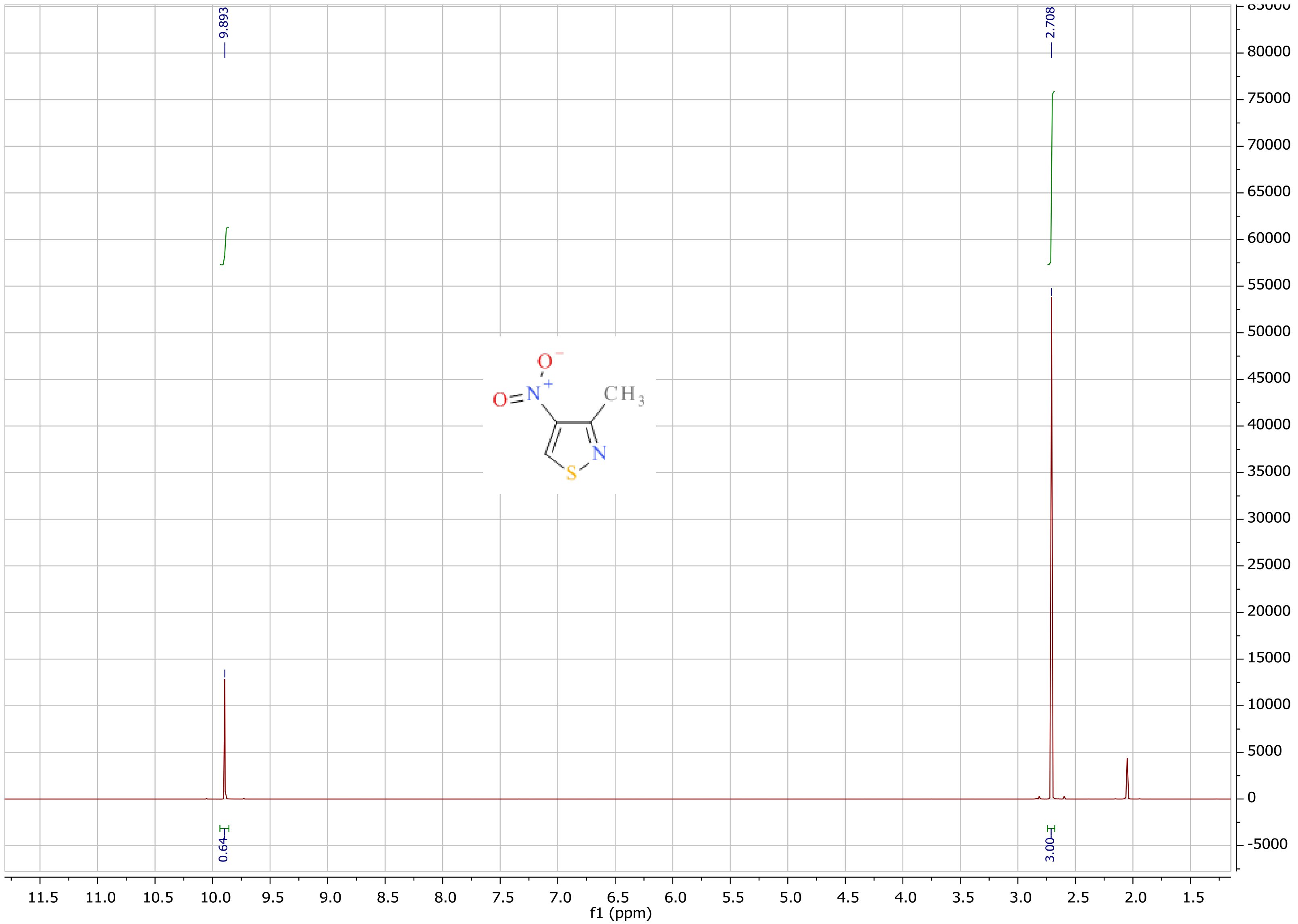

Figure S1. ^{1}H NMR (600.13 MHz, acetone-d_6) spectrum of 3-methyl-4-nitroisothiazole (**L1**).

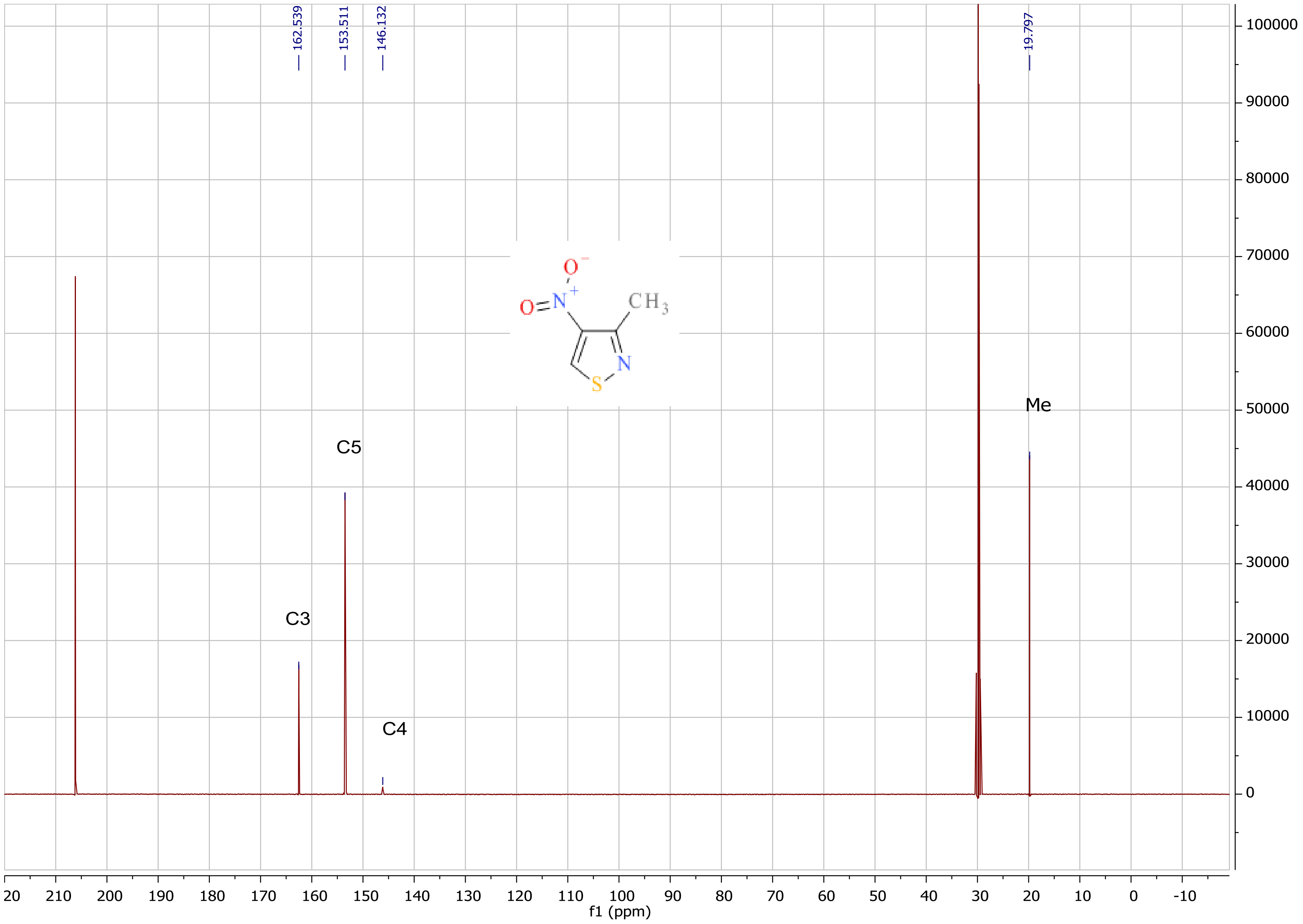

Figure S2. ^{13}C NMR (150.92 MHz, acetone-d_6) spectrum of 3-methyl-4-nitroisothiazole (**L1**).

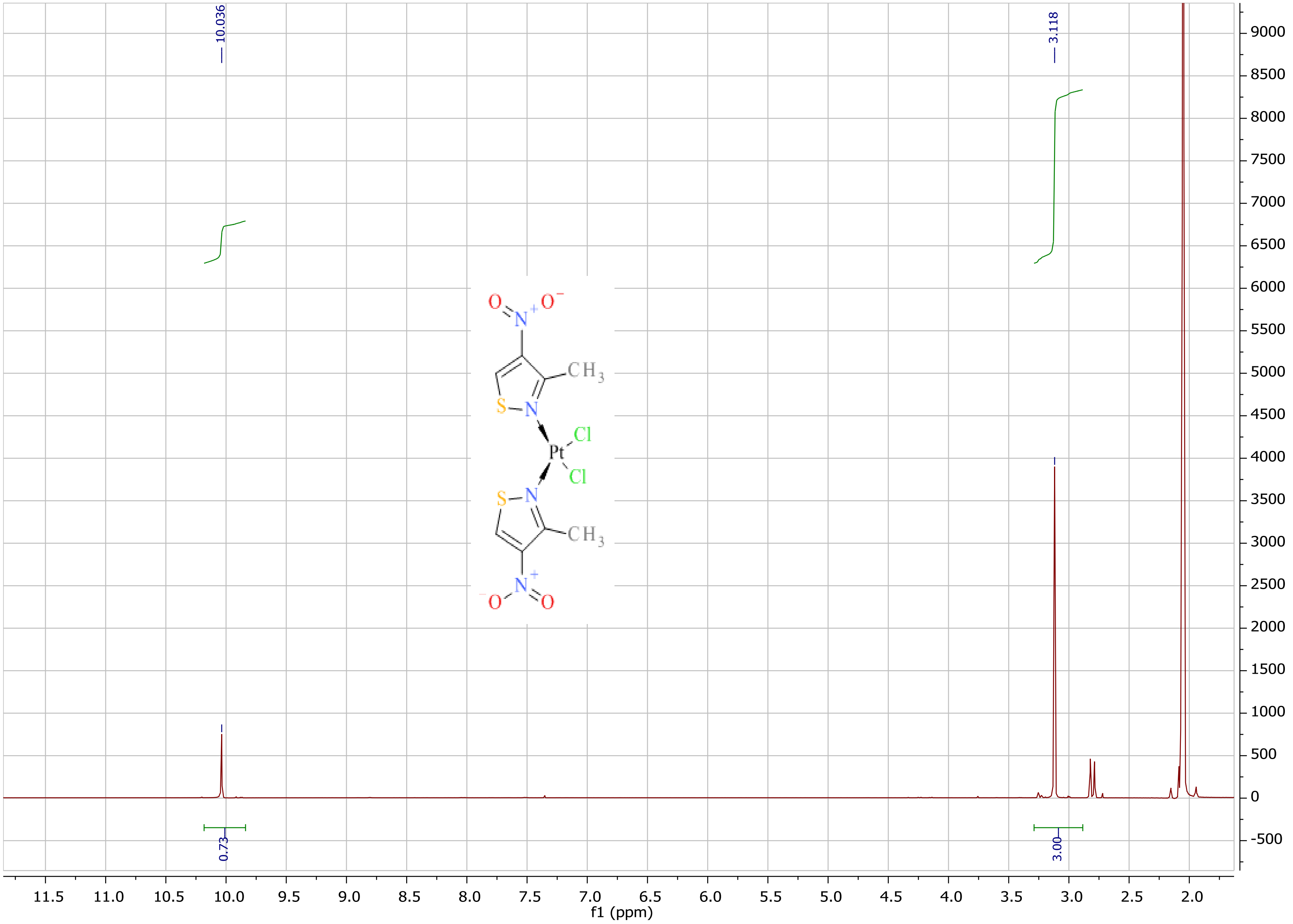

Figure S3 . ^{1}H NMR (600.13 MHz, acetone-d_6) spectrum of *cis*-dichlorobis(3-methyl-4-nitroisothiazole)platinum(II) (**C1**).

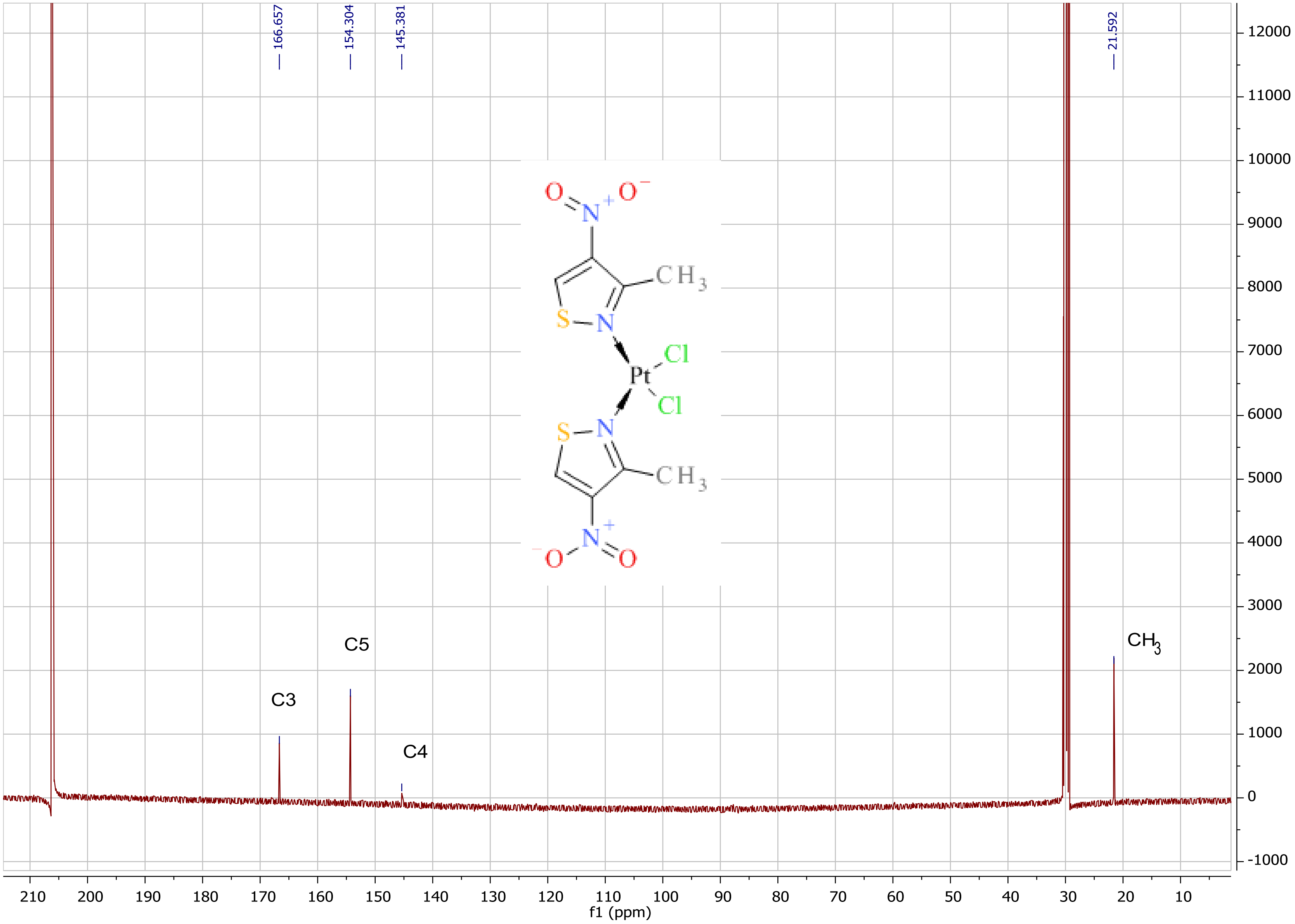

Figure S4. ^{13}C NMR (150.92 MHz, acetone-d_6) spectrum of *cis*-dichlorobis(3-methyl-4-nitroisothiazole)platinum(II) (**C1**).

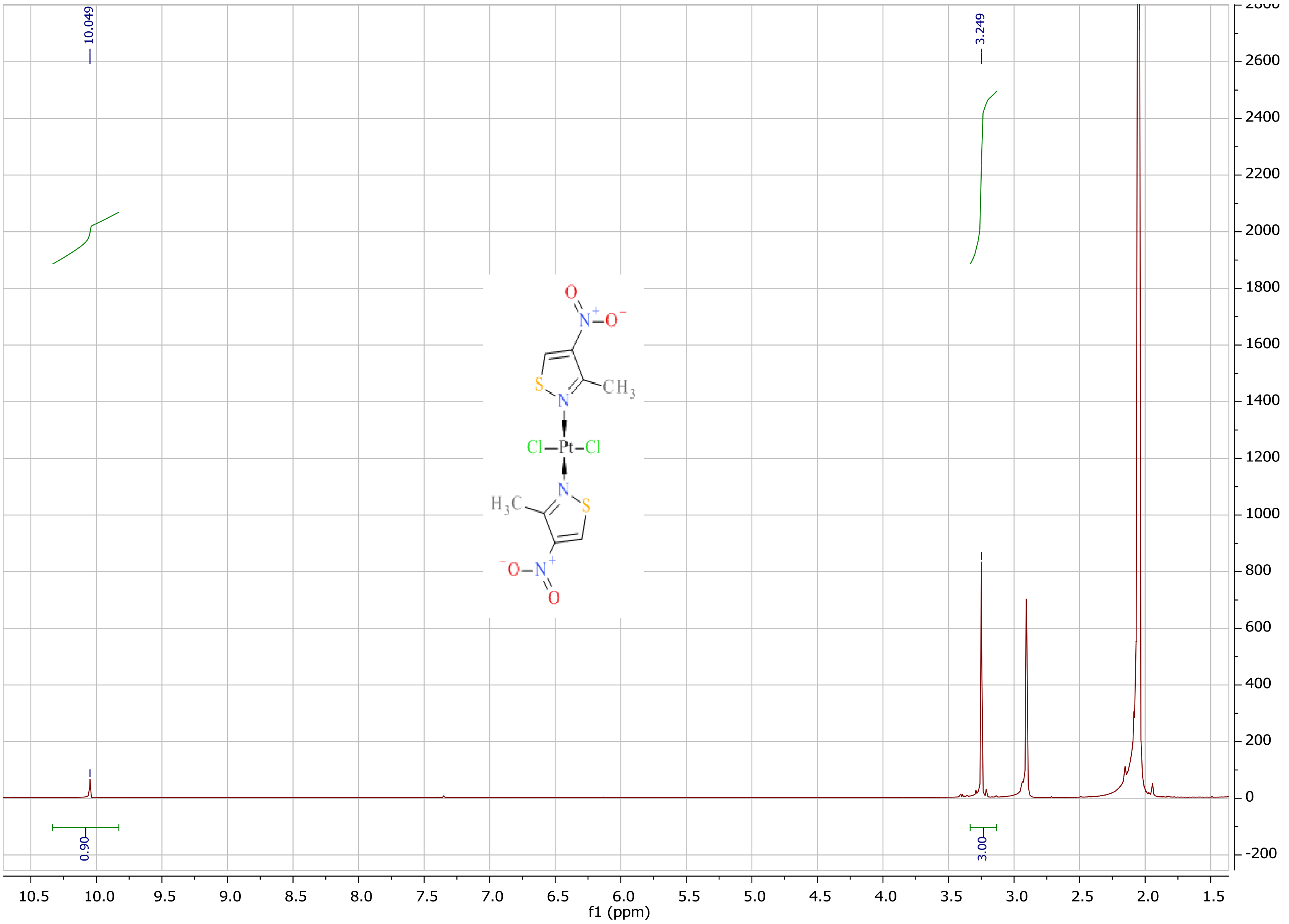

Figure S5. ^{1}H NMR (600.13 MHz, acetone-d_6) spectrum of *trans*-dichlorobis(3-methyl-4-nitroisothiazole)platinum(II) (**C2**).

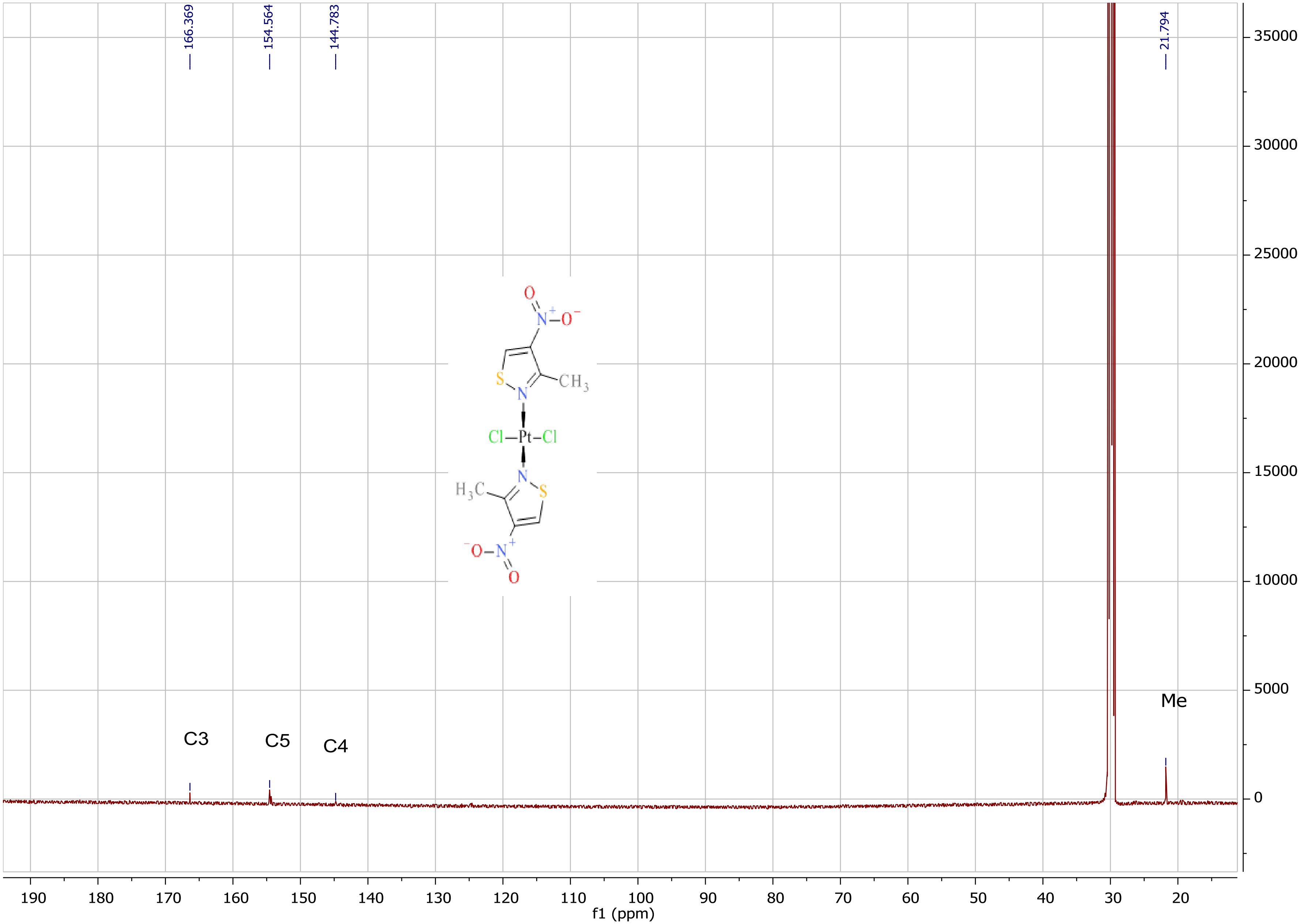

Figure S6. ^{13}C NMR (150.92 MHz, acetone-d_6) spectrum of *trans*-dichlorobis(3-methyl-4-nitroisothiazole)platinum(II) (**C2**).

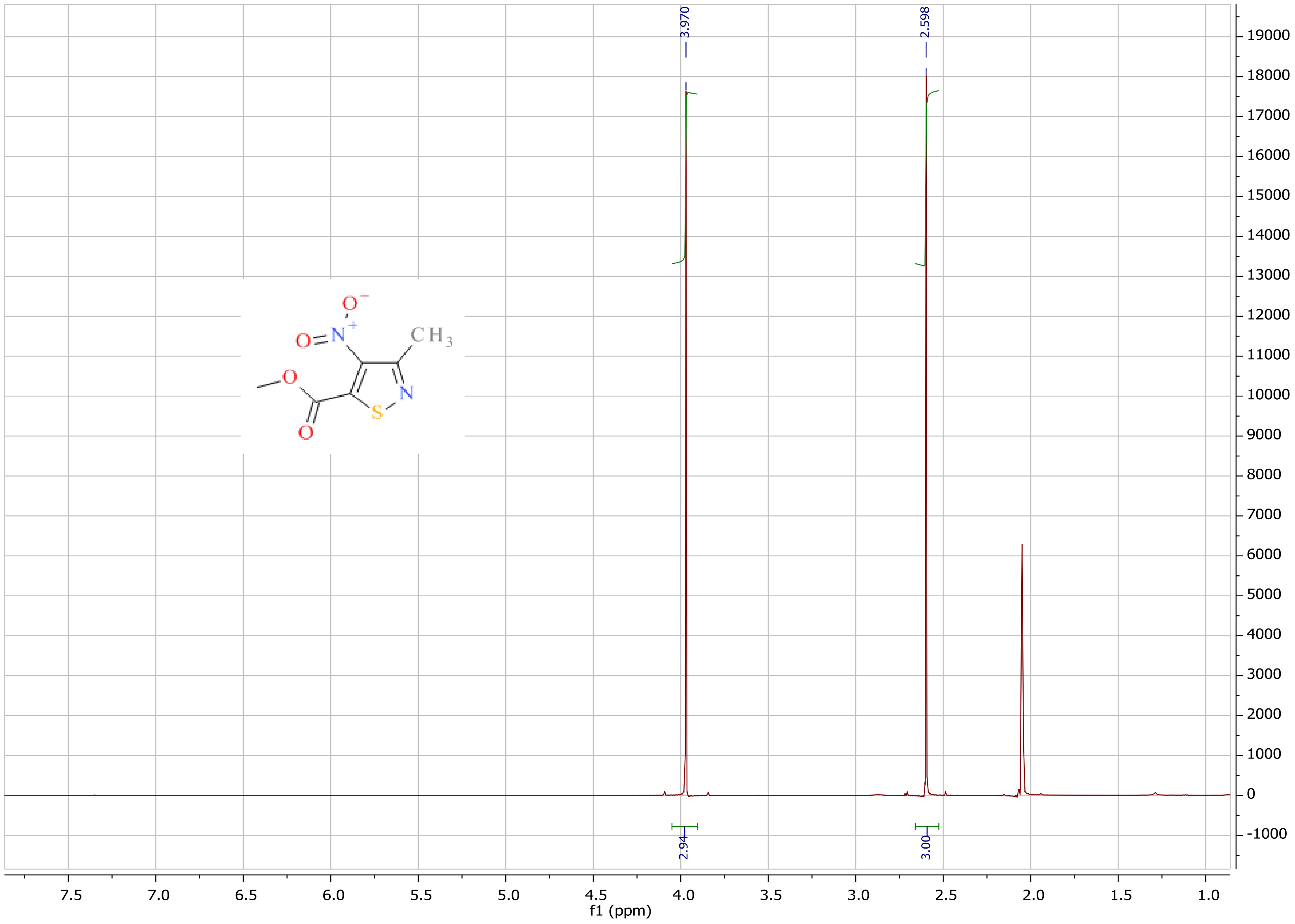

Figure S7. ^{1}H NMR (600.13 MHz, acetone-d_6) spectrum of methyl 3-methyl-4-nitro-5-isothiazolecarboxylate (**L2**).

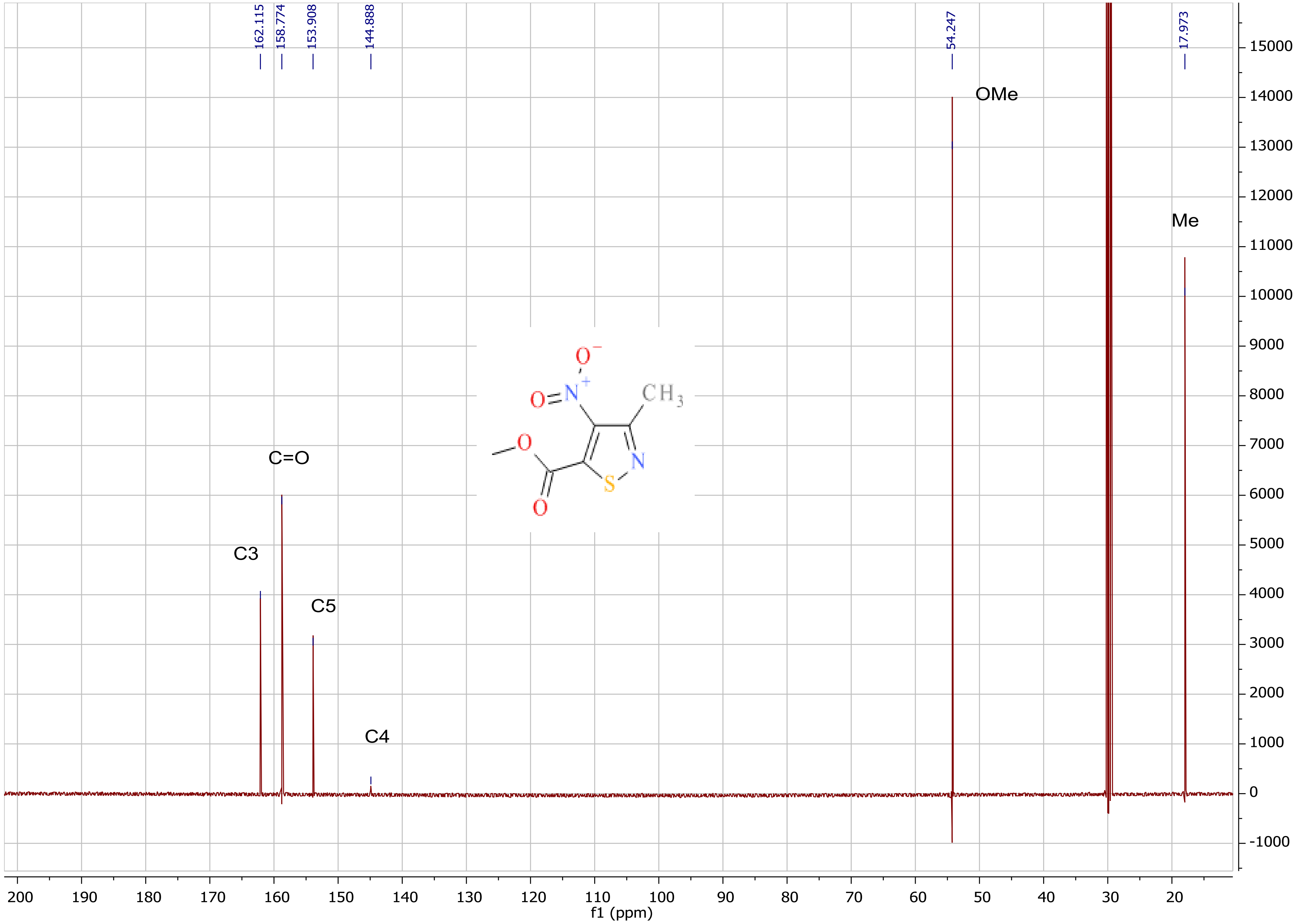

Figure S8. ^{13}C NMR (150.92 MHz, acetone-d_6) spectrum of methyl 3-methyl-4-nitro-5-isothiazolecarboxylate (**L2**).

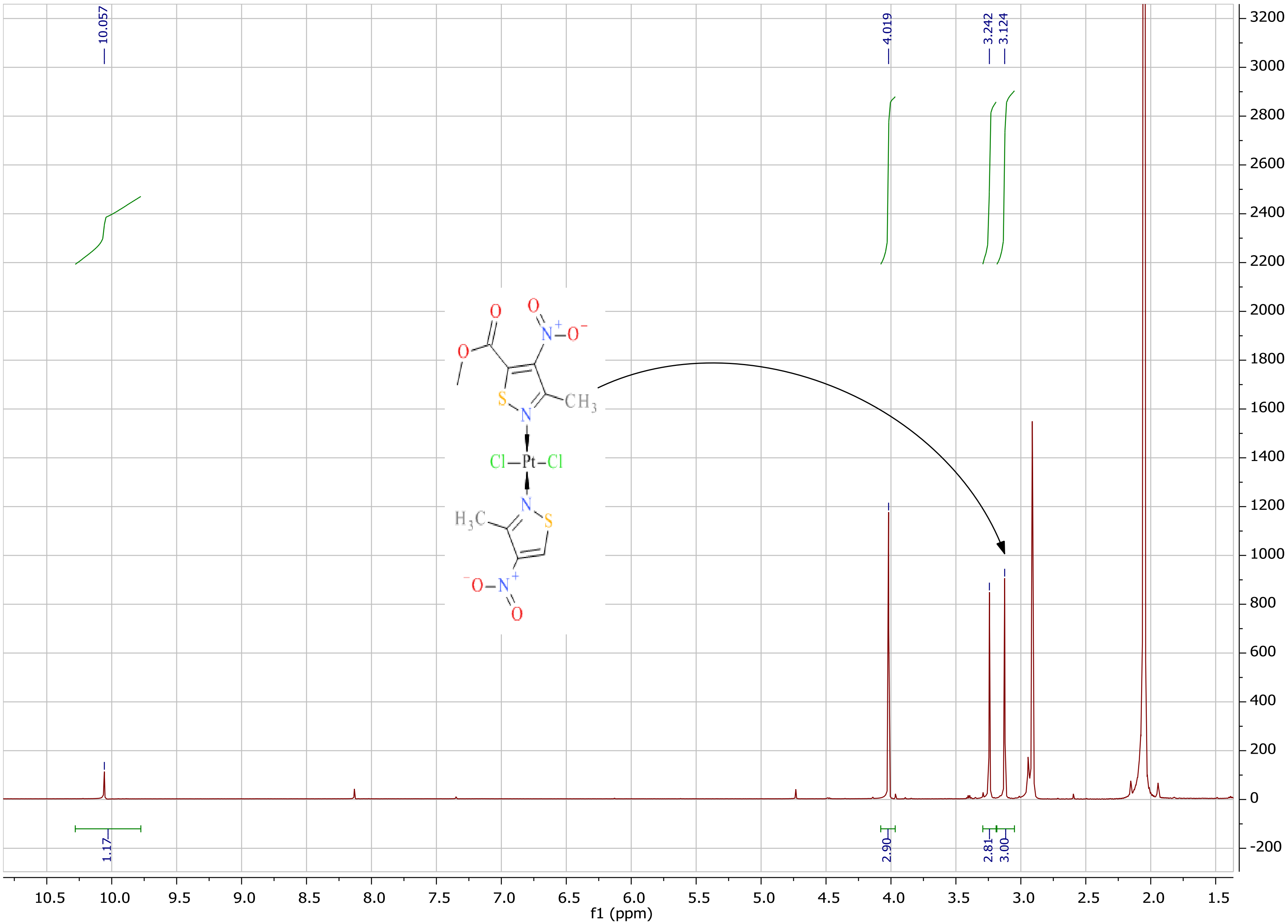

Figure S9. ^{1}H NMR (600.13 MHz, acetone-d_6) spectrum of *trans*-dichloro-3-methyl-4-nitroisothiazole 3-methyl-4-nitro-5-(methoxycarbonyl)isothiazole platinum(II) (**C3**).

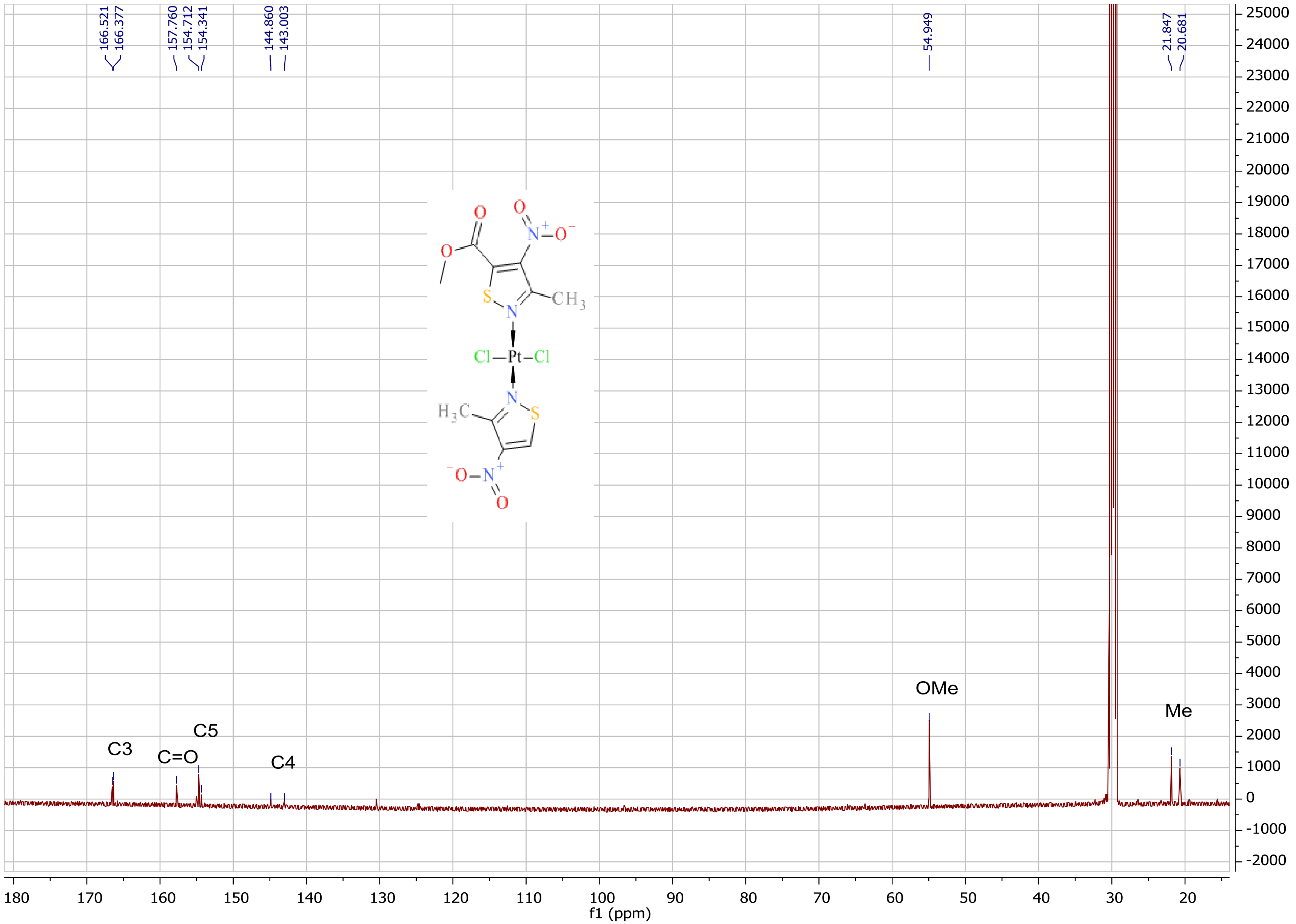

Figure S10. ^{13}C NMR (150.92 MHz, acetone-d$_6$) spectrum of *trans*-dichloro-3-methyl-4-nitroisothiazole 3-methyl-4-nitro-5-(methoxycarbonyl)isothiazole platinum(II) (**C3**).

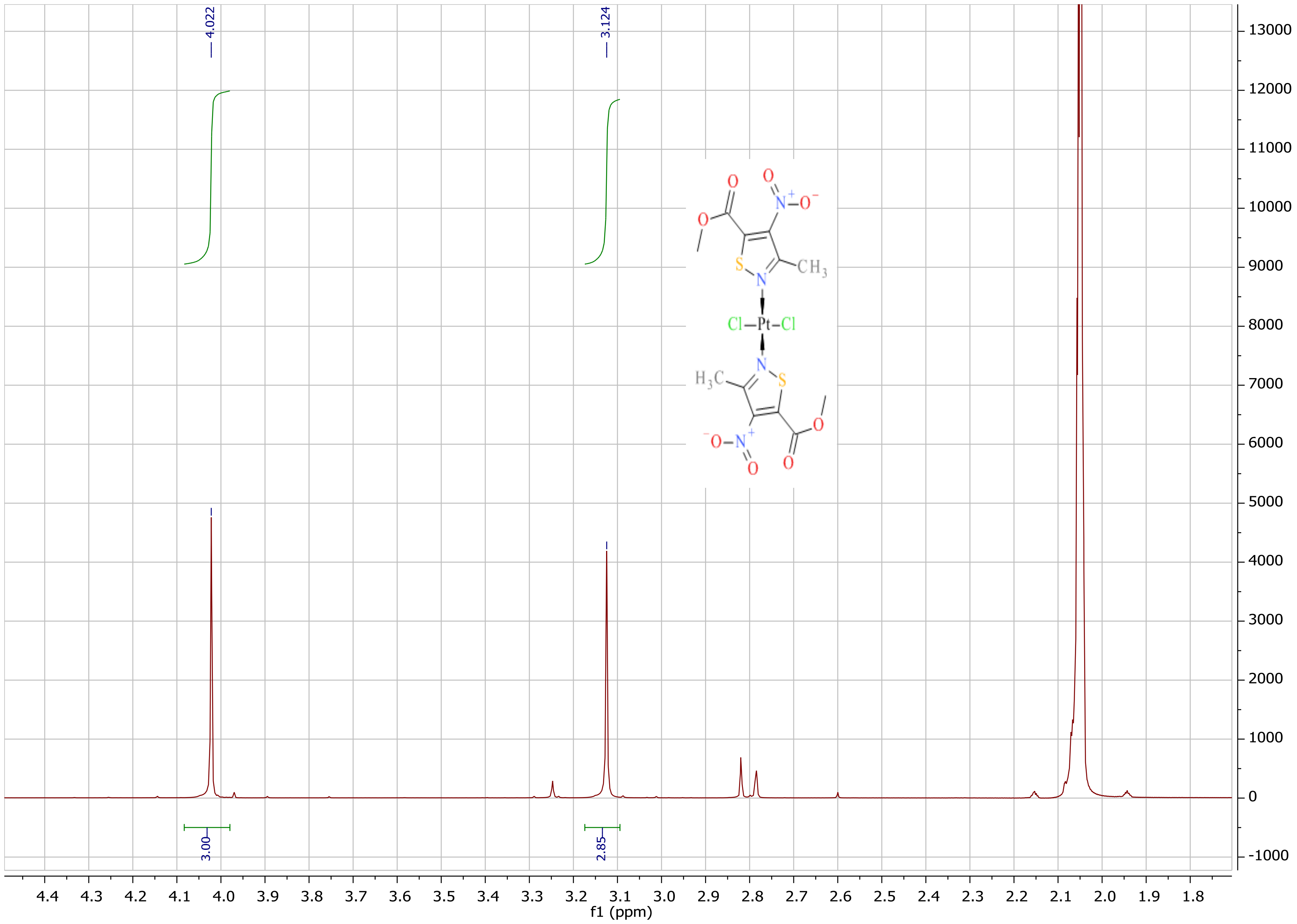

Figure S11. ^{1}H NMR (600.13 MHz, acetone-d_6) spectrum of *trans*-dichlorobis(3-methyl-4-nitro-5-(methoxycarbonyl)isothiazole)platinum(II) (**C4**).

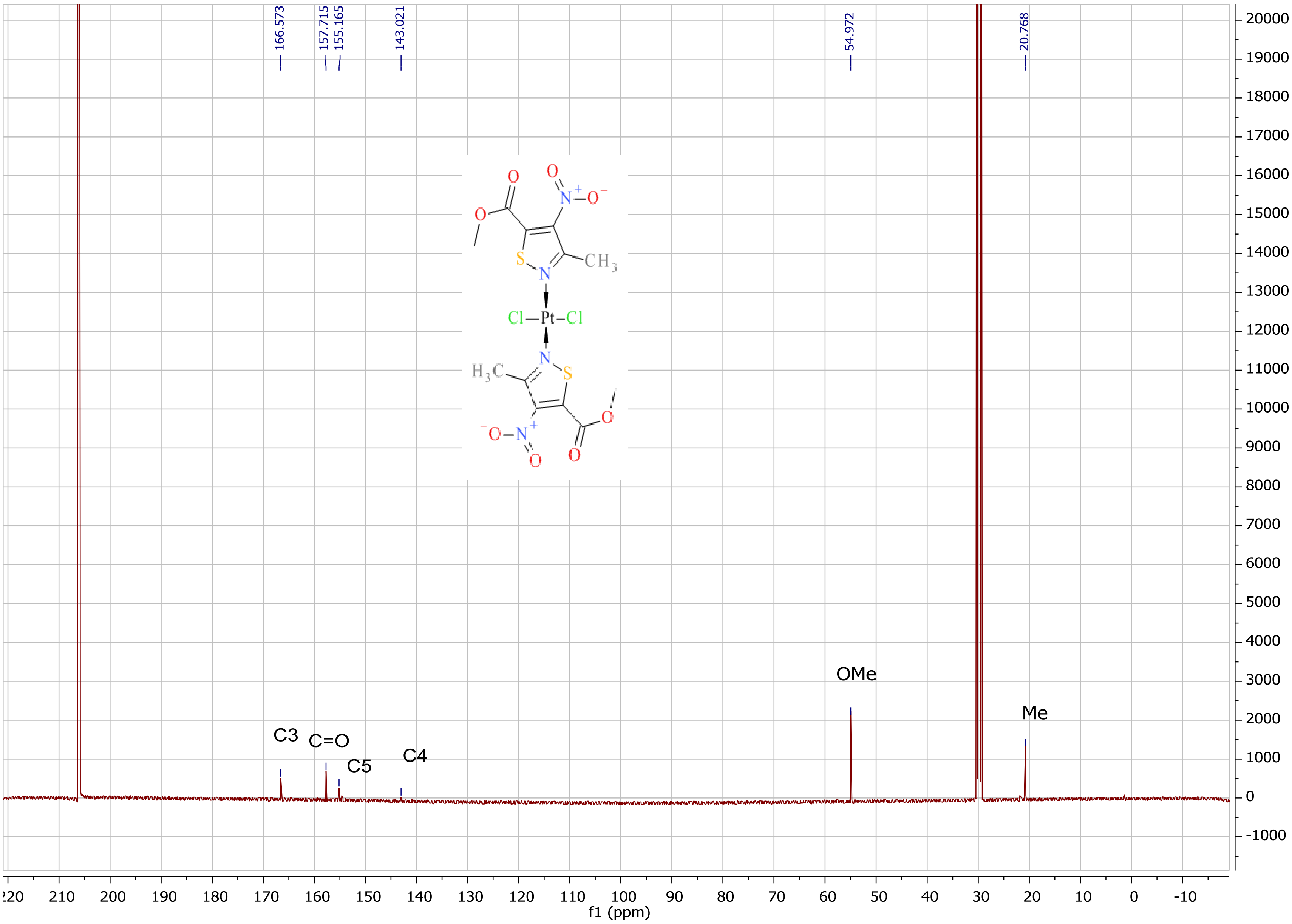

Figure S12. ^{13}C NMR (150.92 MHz, acetone-d_6) spectrum of *trans*-dichlorobis(3-methyl-4-nitro-5-(methoxycarbonyl)isothiazole)platinum(II) (**C4**).

.

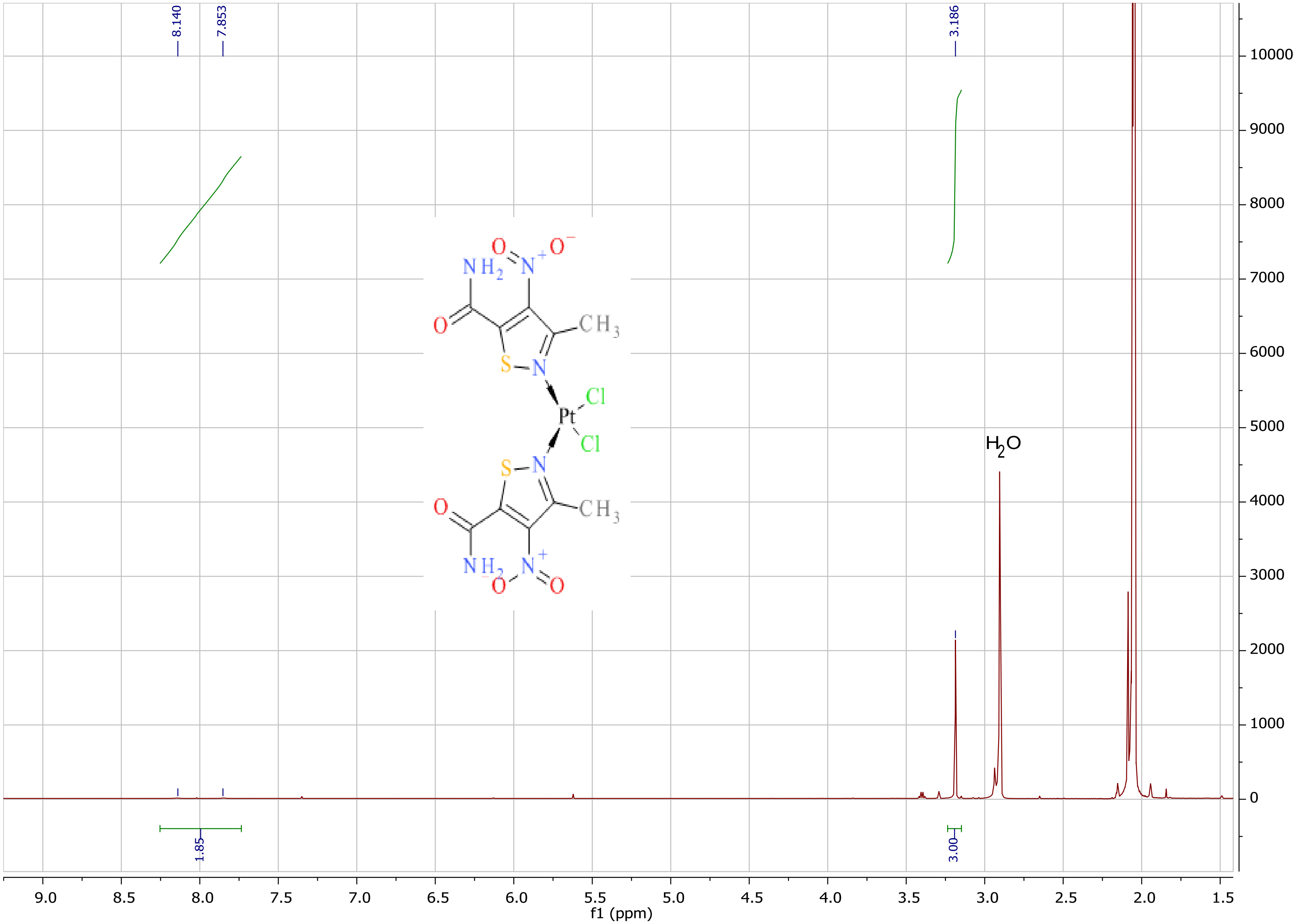

Figure S13. ^{1}H NMR (600.13 MHz, acetone-$d_{6,}$ relaxation delay=2s, T=298) spectrum of *cis*-dichlorobis(3-methyl-4-nitroisothiazole-5-carboxamide)platinum(II) (**C5**).

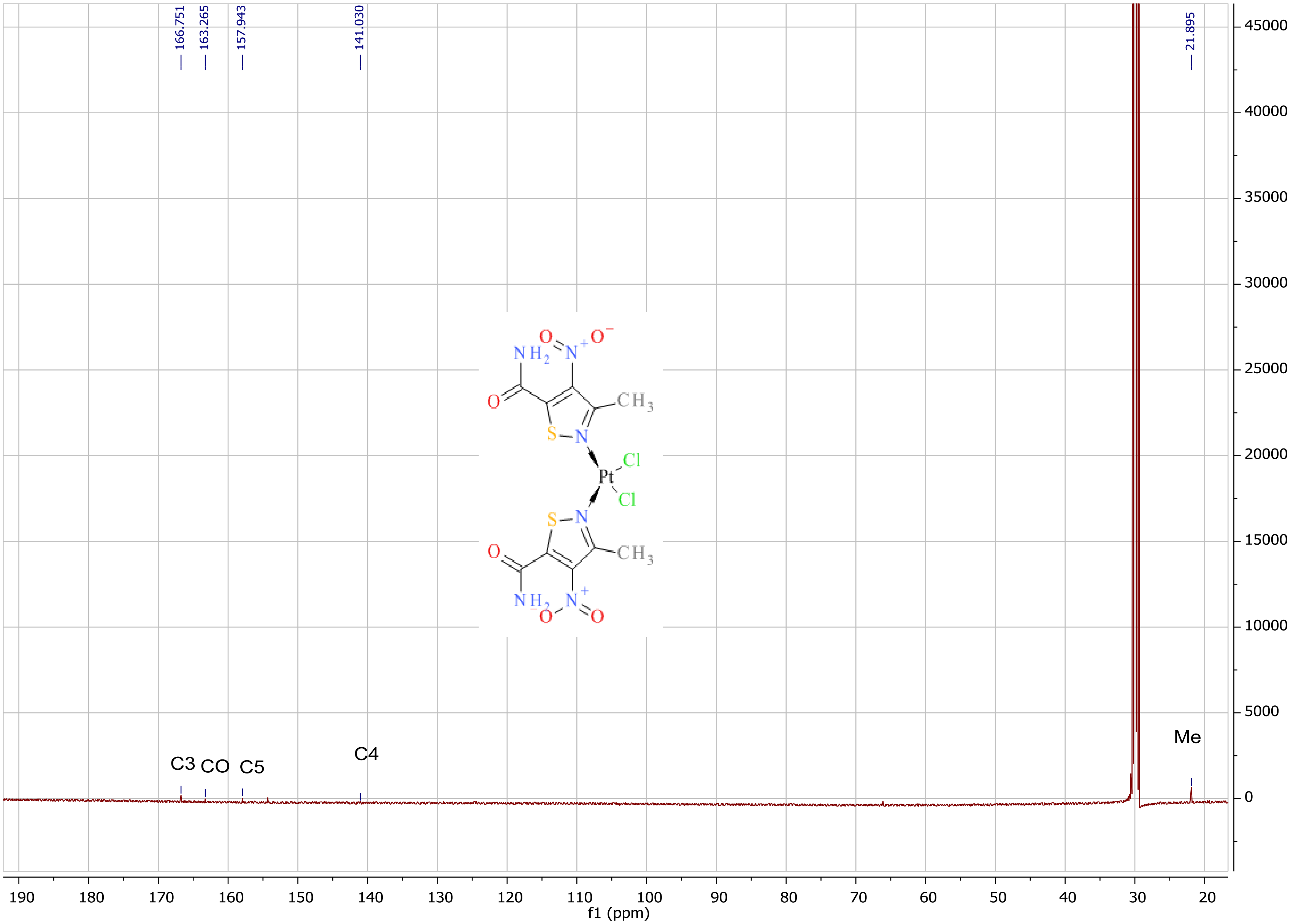

Figure S14. ^{13}C NMR (150.92 MHz, acetone-d$_6$, relaxation delay=3s, T=298K) spectrum of *cis*-dichlorobis(3-methyl-4-nitroisothiazole-5-carboxamide)platinum(II) (**C5**).

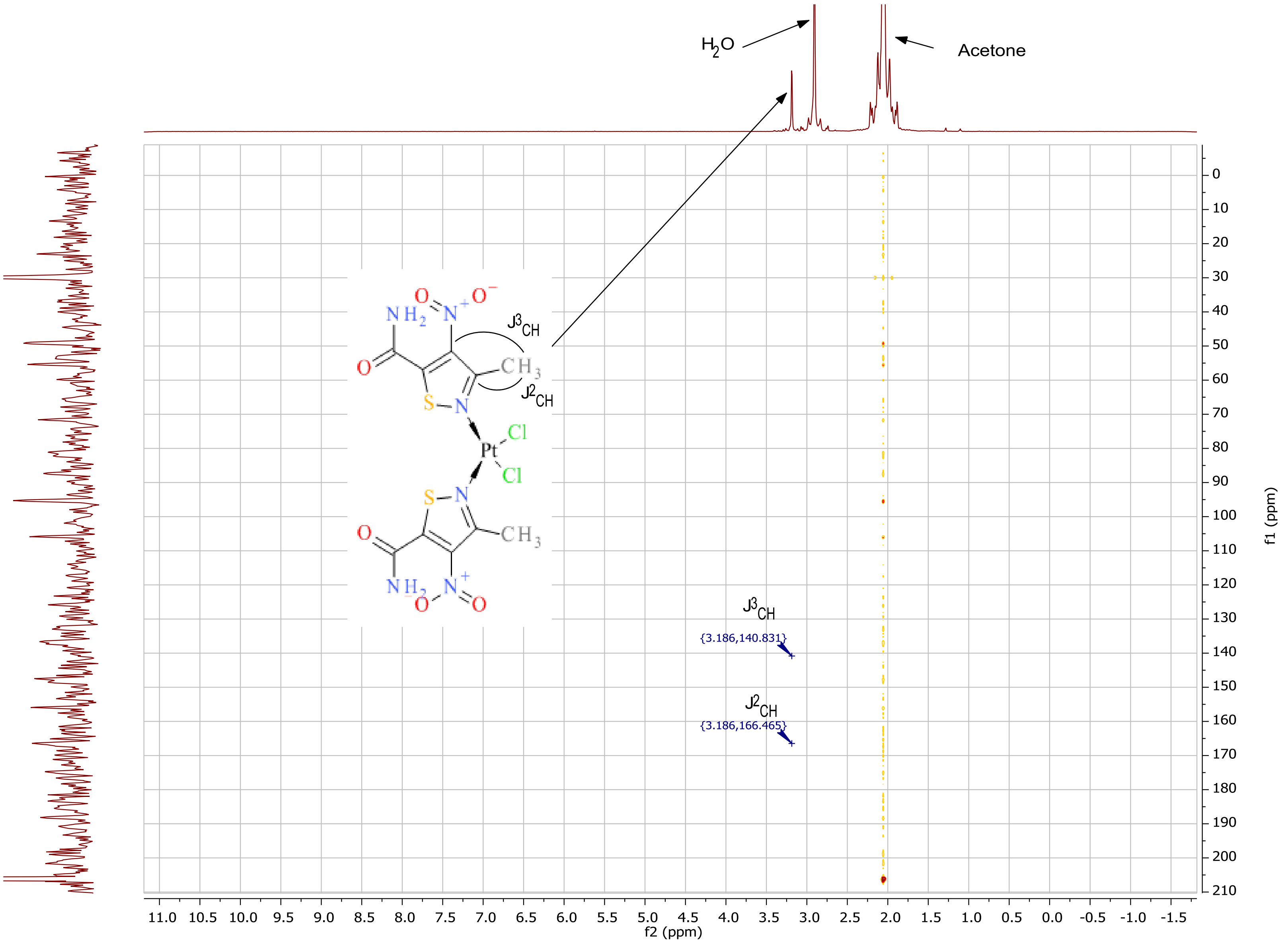

Figure S15. 2D HMBC (Phase cycled magnitude-mode 2D HMBC using low-pass J-filter (hmbclpndqf | HMBCLPND was used), acetone-d_6, F2=600.13 MHz, F1=150.92 MHz, acetone-d_6, 32 scans, relaxation delay=1.5s, aqt=0.2621s, T=298K) spectrum of *cis*-dichlorobis(3-methyl-4-nitroisothiazole-5-carboxamide)platinum(II) (**C5**).

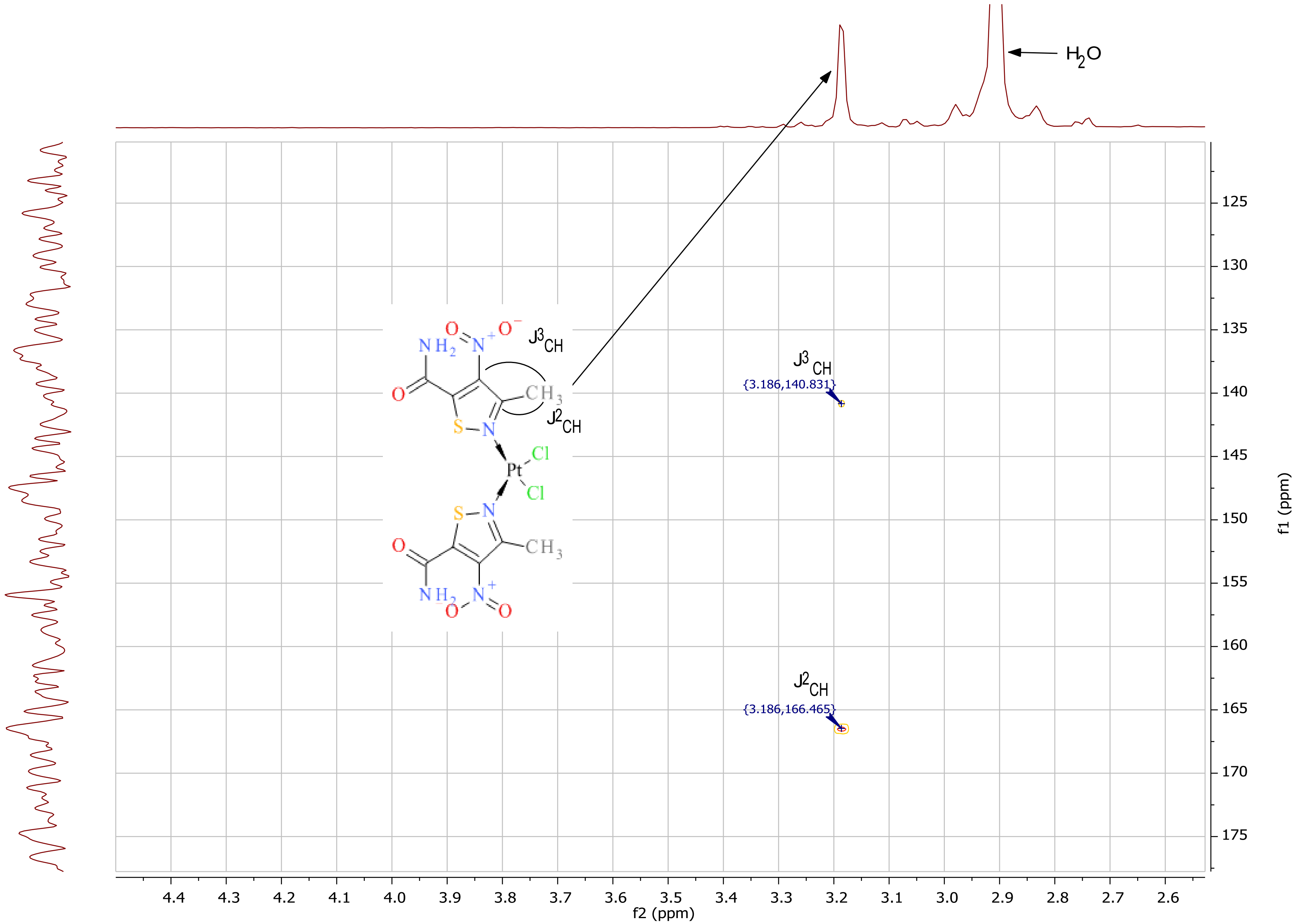

Figure S16. 2D HMBC (Phase cycled magnitude-mode 2D HMBC using low-pass J-filter (hmbclpndqf | HMBCLPND), acetone-d_6, F2=600.13 MHz, F1=150.92 MHz, acetone-d_6, 32 scans, relaxation delay=1.5s, aqt=0.2621s, T=298K) spectrum of *cis*-dichlorobis(3-methyl-4-nitroisothiazole-5-carboxamide)platinum(II) (**C5**).

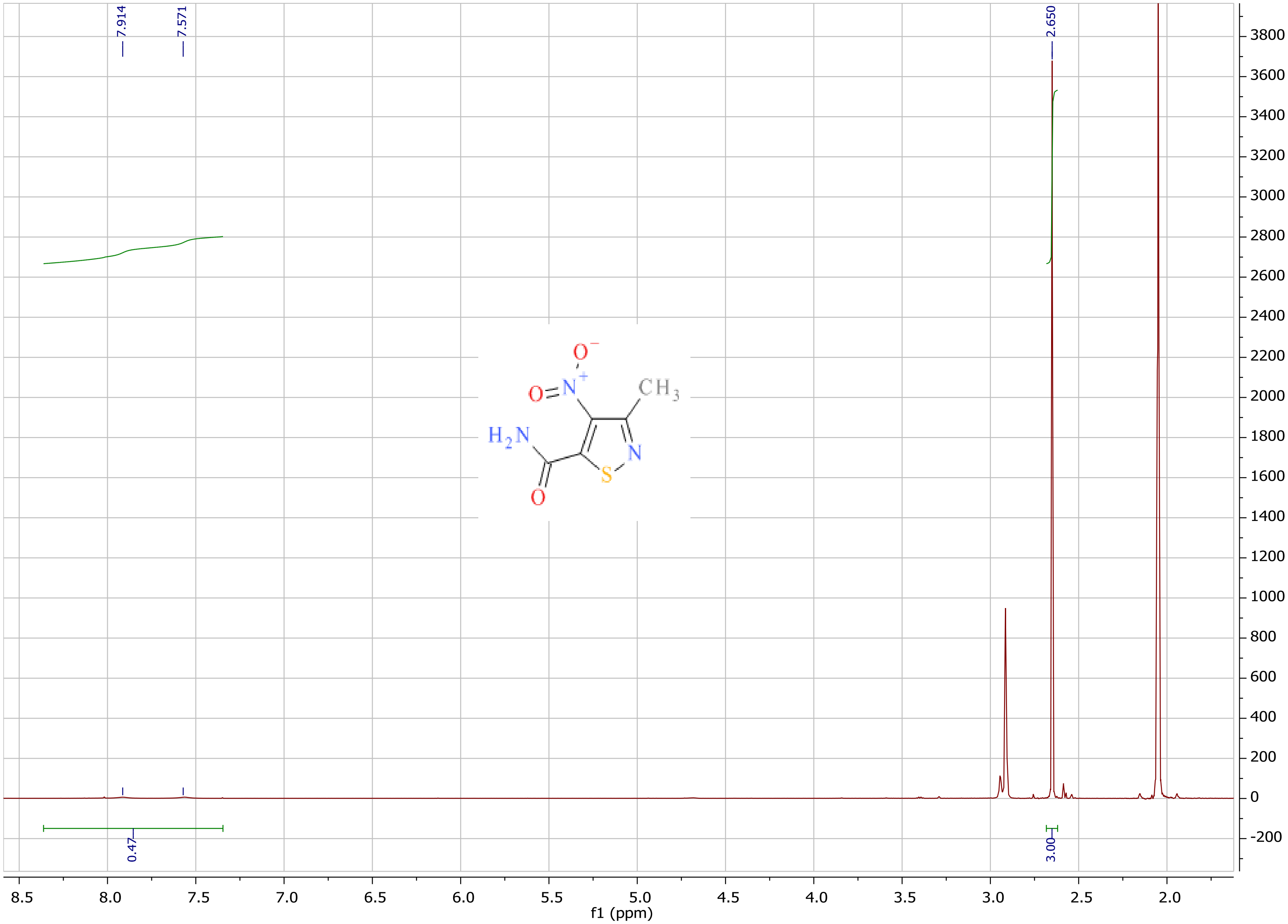

Figure S17. ^{1}H NMR (600.13 MHz, acetone-d$_6$) spectrum of 3-methyl-4-nitroisothiazole-5-carboxamide (**L4**).

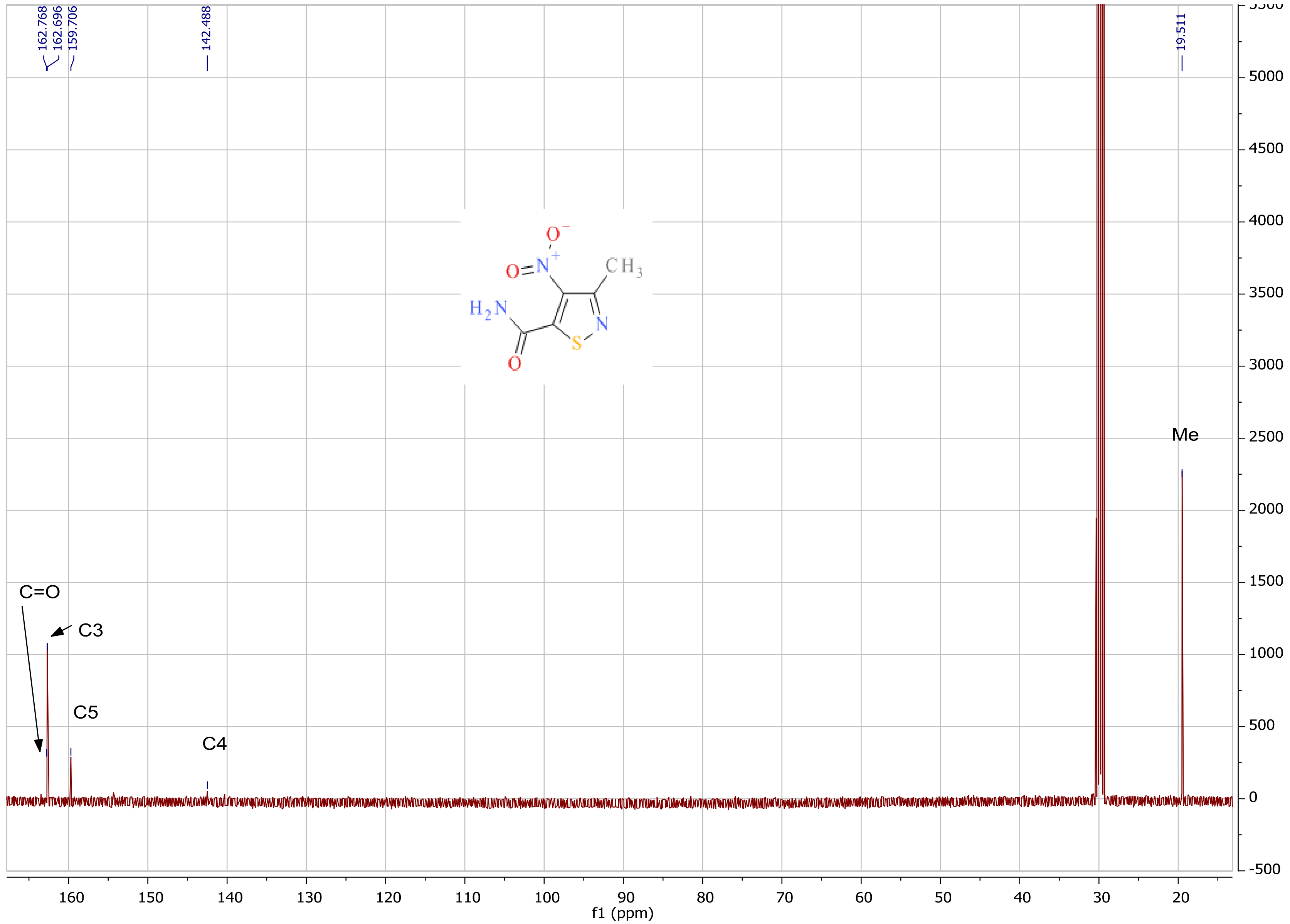

Figure S18. ^{13}C NMR (150.92 MHz, acetone-d_6) spectrum of 3-methyl-4-nitroisothiazole-5-carboxamide (**L4**).

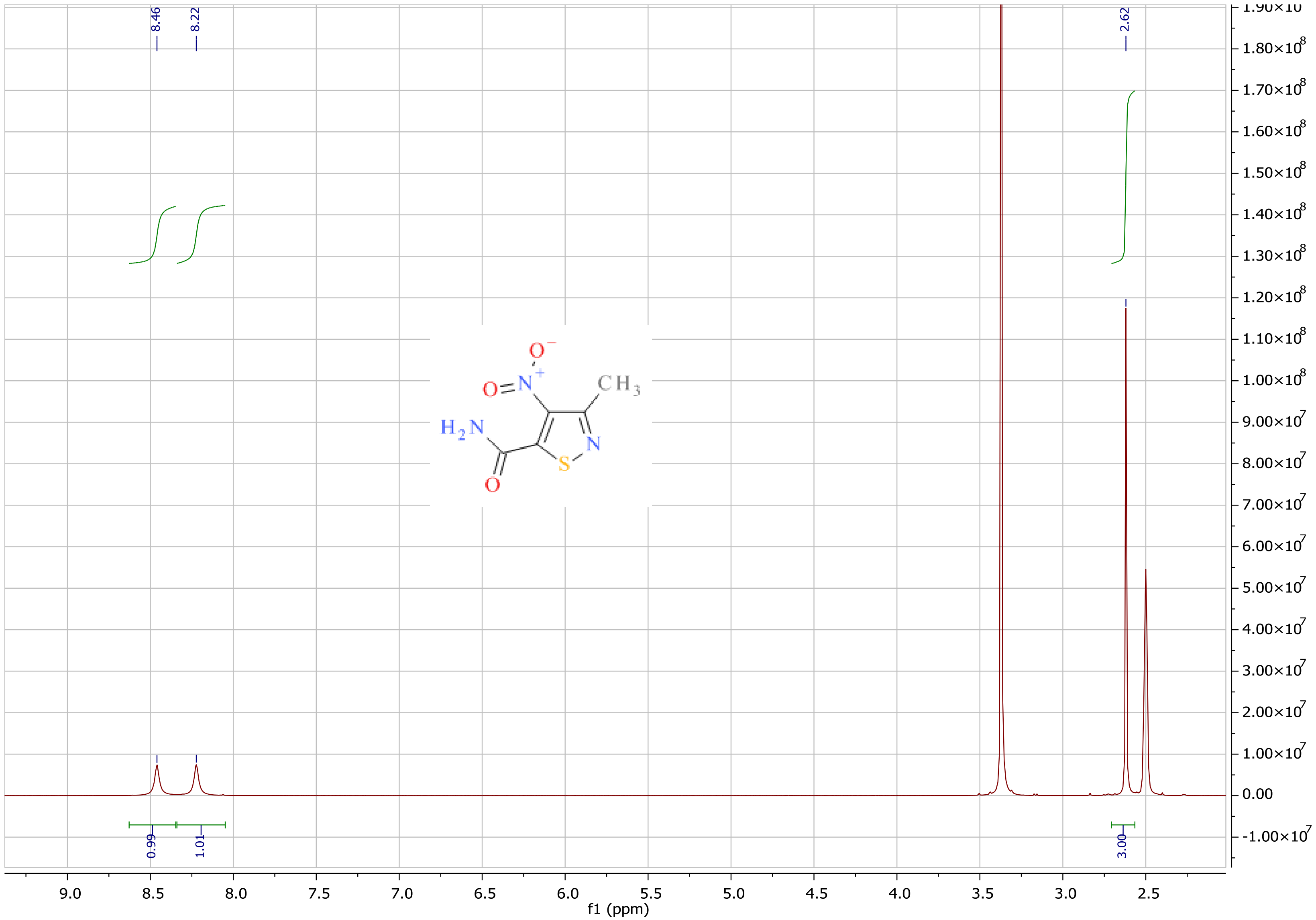

Figure S19. ^{1}H NMR (300.15 MHz, DMSO-d_6, T=295.4K) spectrum of 3-methyl-4-nitroisothiazole-5-carboxamide (**L4**).

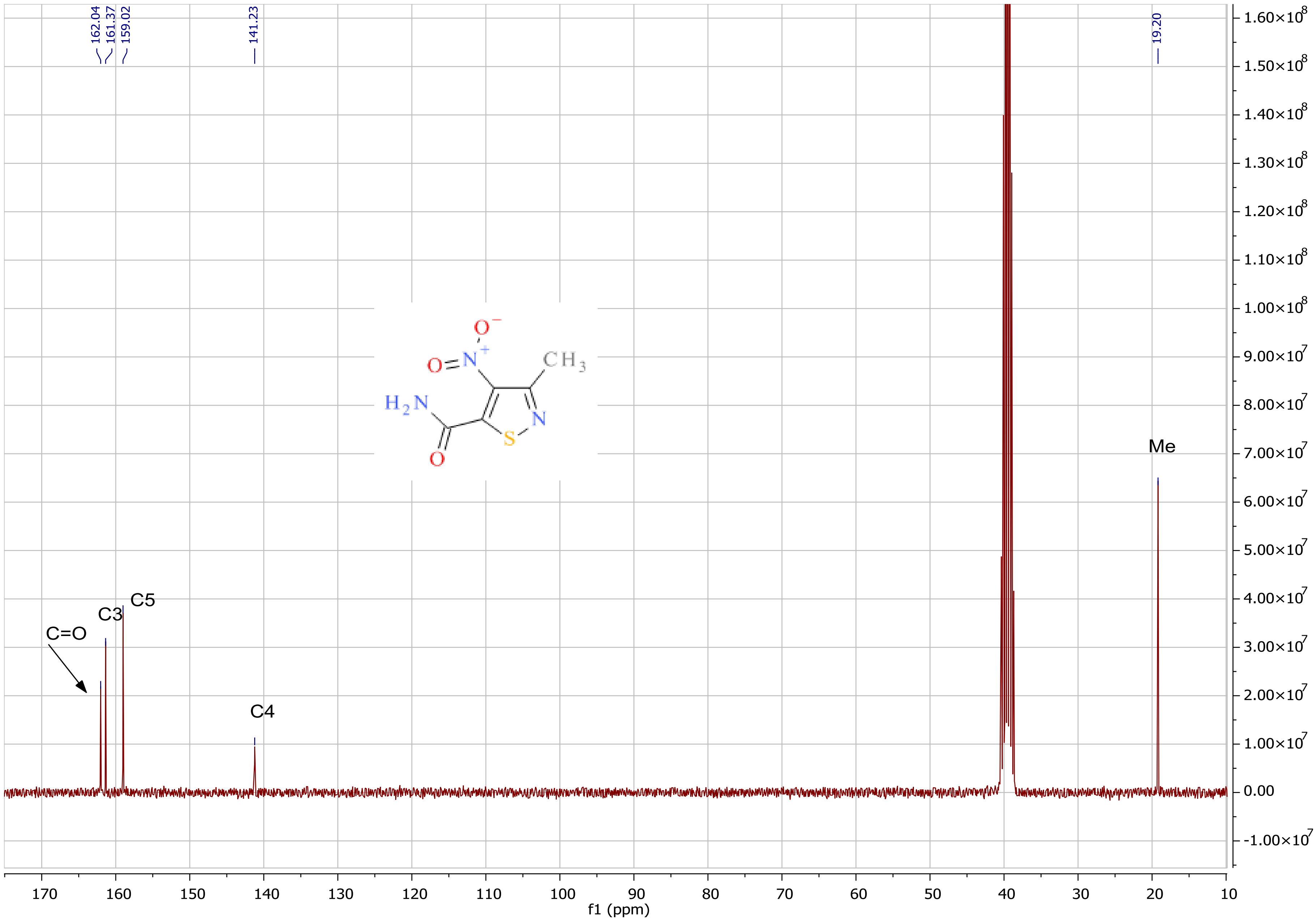

Figure S20. ^{13}C NMR (75.47 MHz, DMSO-d_6, T=296.6K) spectrum of 3-methyl-4-nitroisothiazole-5-carboxamide (**L4**).

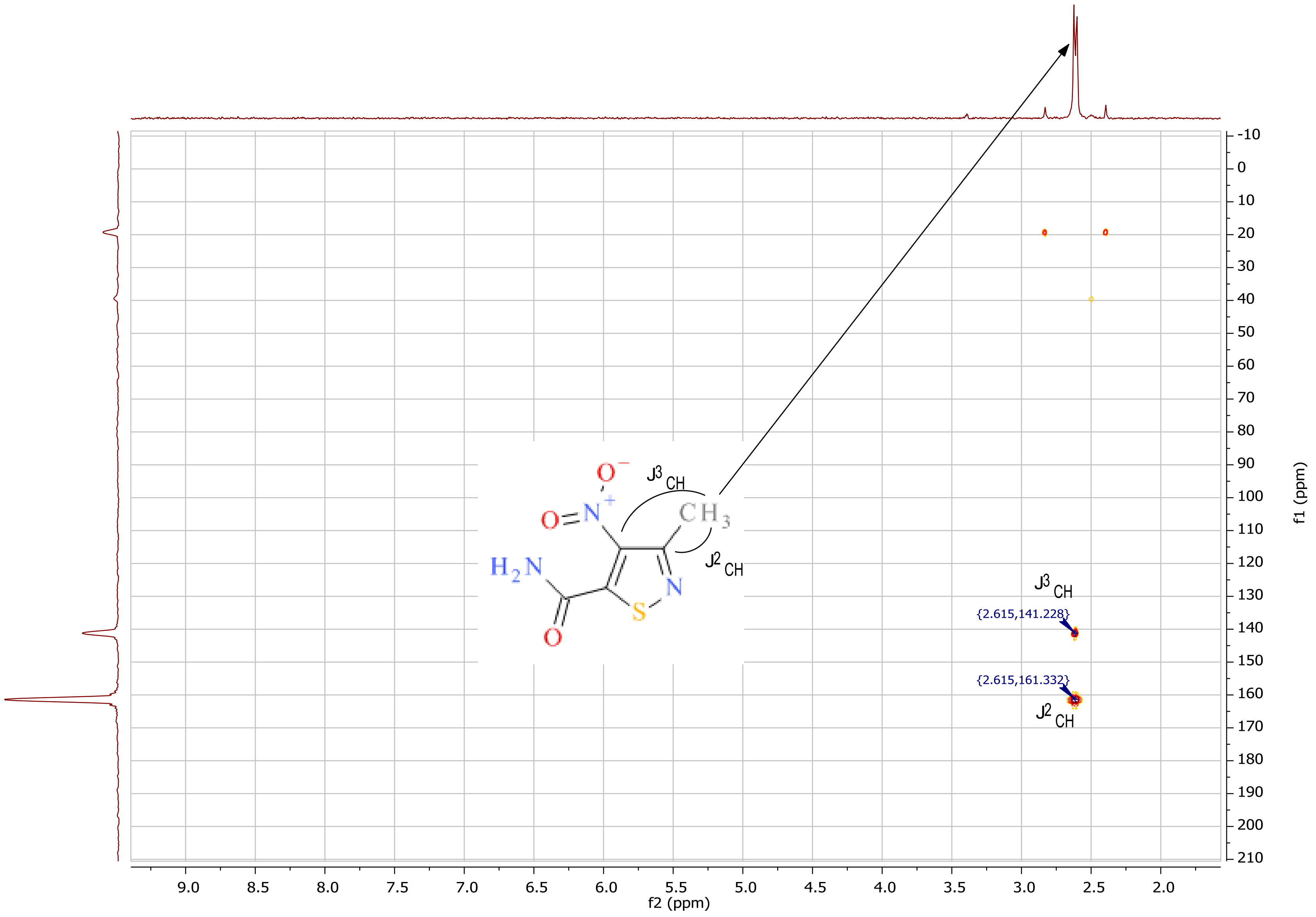

Figure S21. 2D HMBC (Gradient-based magnitude-mode ge-2D HMBC using low-pass J-filter (hmbcgplpndqf |HMBCGPLPND, DMSO-d_6, F2=300.15 MHz, F1=75.47 MHz, 32 scans, relaxation delay=1.1518s, aqt=0.8717s, T=299.3K) spectrum of 3-methyl-4-nitroisothiazole-5-carboxamide (**L4**).

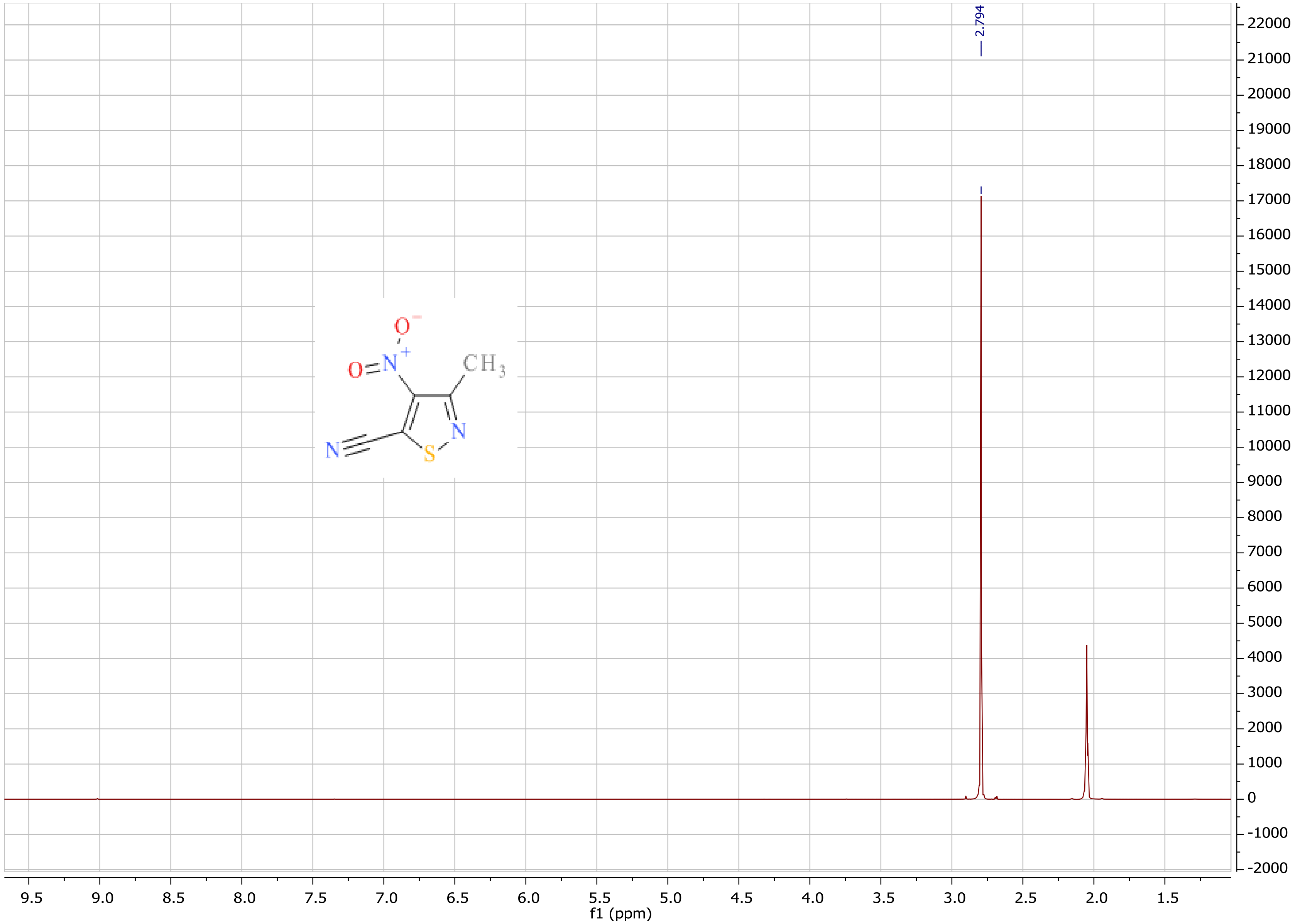

Figure S22. ^{1}H NMR (600.13 MHz, acetone-d$_6$) spectrum of 3-methyl-4-nitroisothiazole-5-carbonitrile (**L3**).

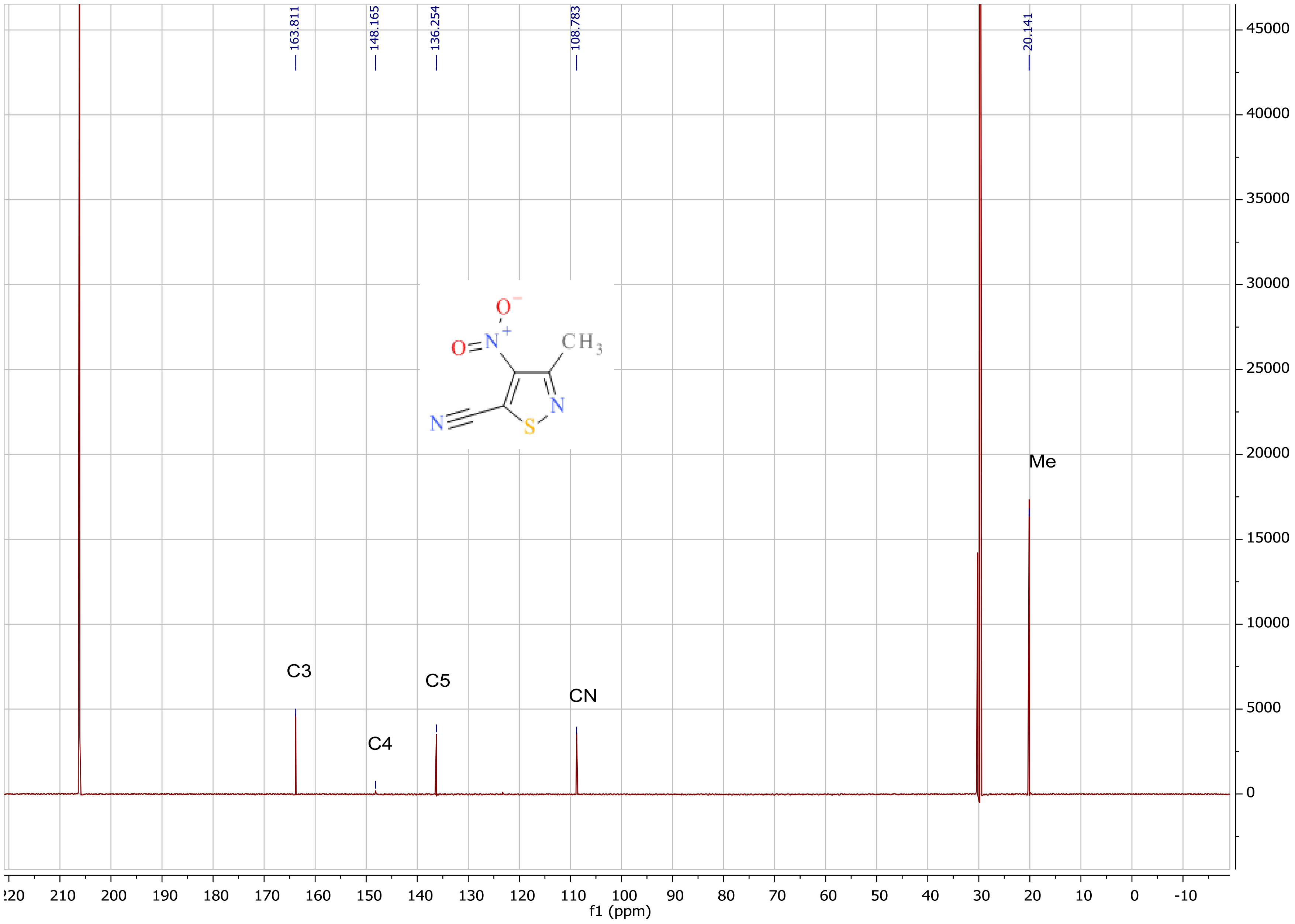

Figure S23. ^{13}C NMR (150.92 MHz, acetone-d_6) spectrum of 3-methyl-4-nitroisothiazole-5-carbonitrile (**L3**).

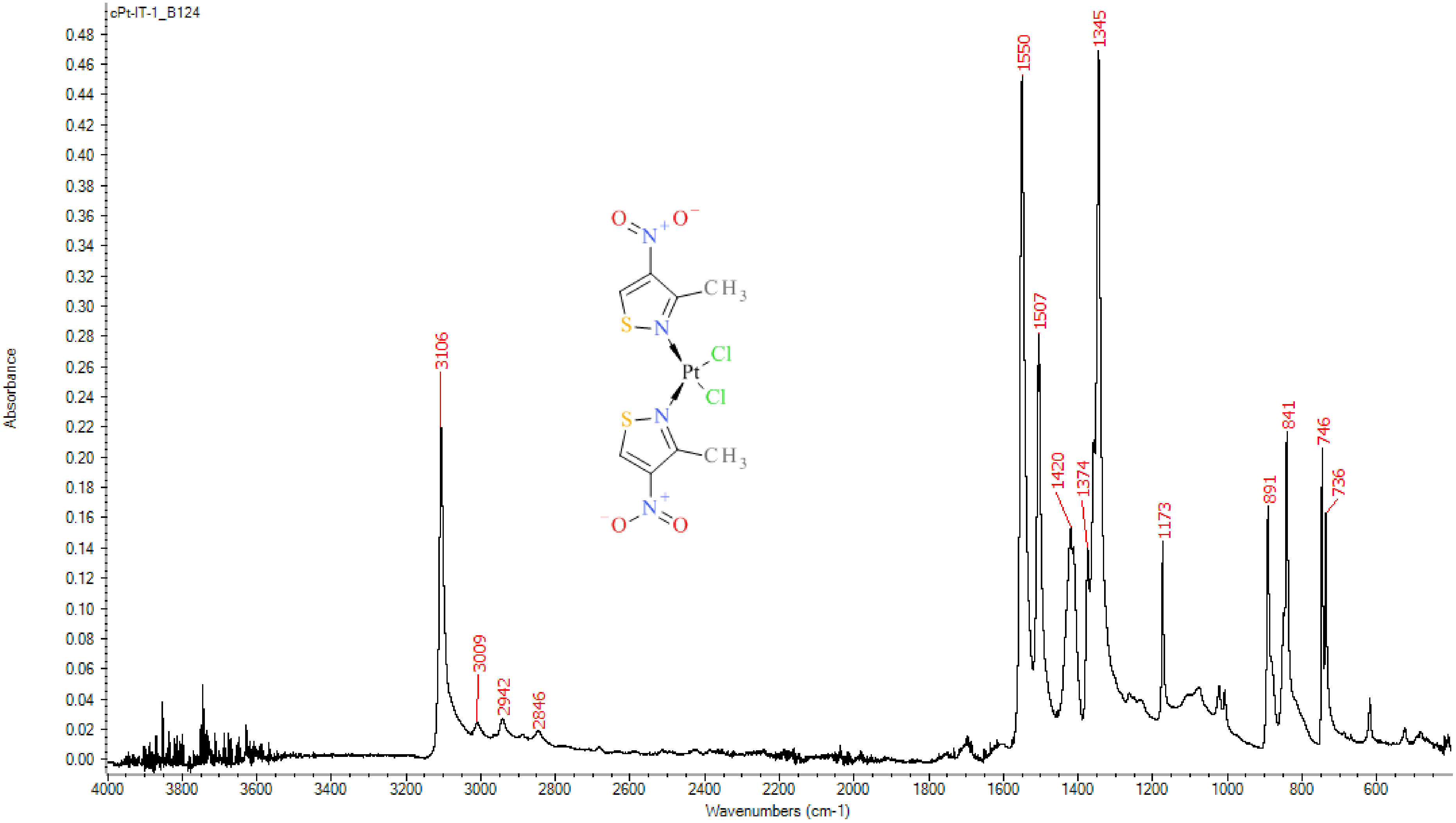

Figure S24. MIR-ATR (Medium Infrared) spectrum of *cis*-dichlorobis(3-methyl-4-nitroisothiazole)platinum(II) (**C1**).

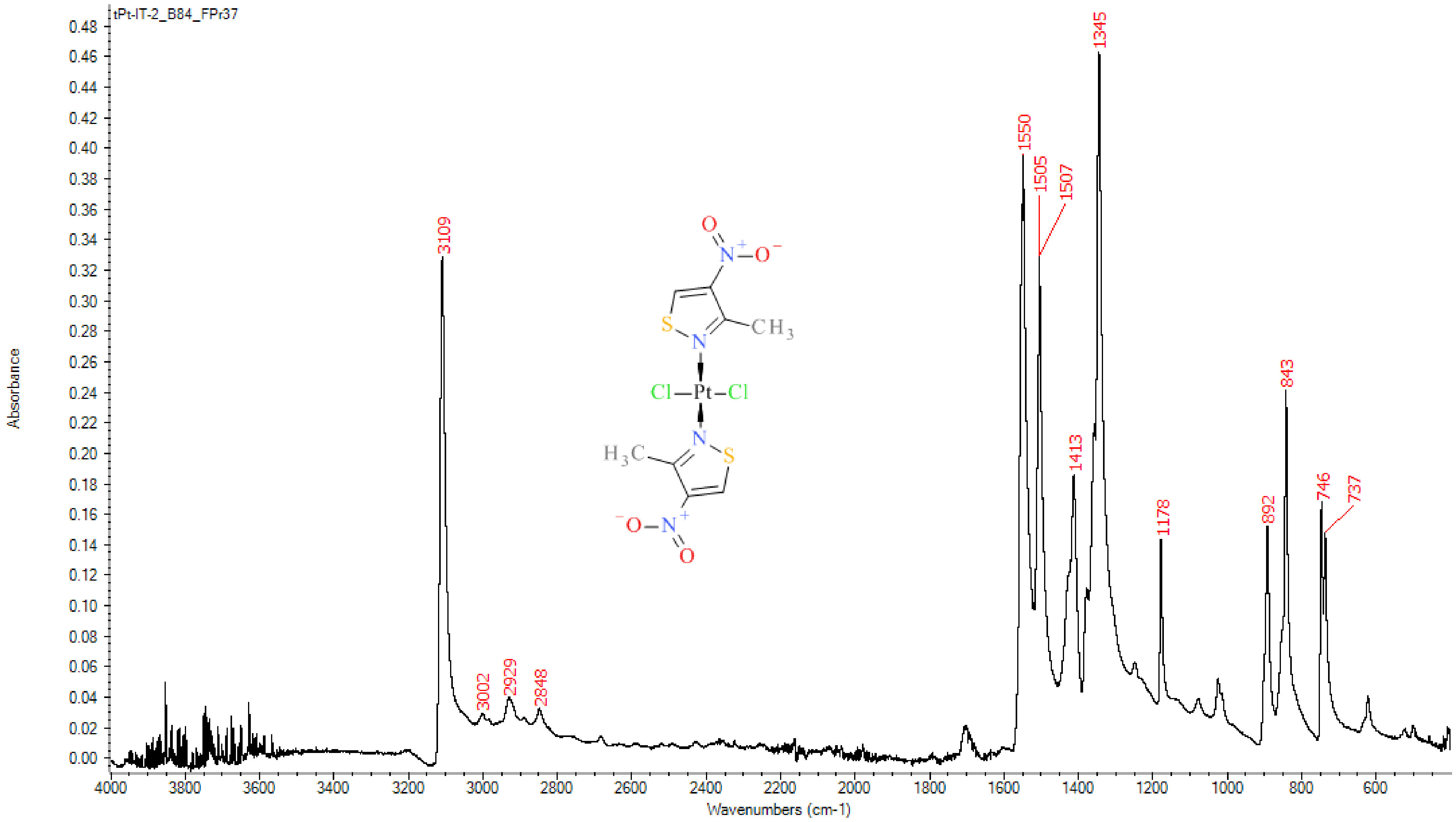

Figure S25. MIR-ATR (Medium Infrared) spectrum of *trans*-dichlorobis(3-methyl-4-nitroisothiazole)platinum(II) (**C2**).

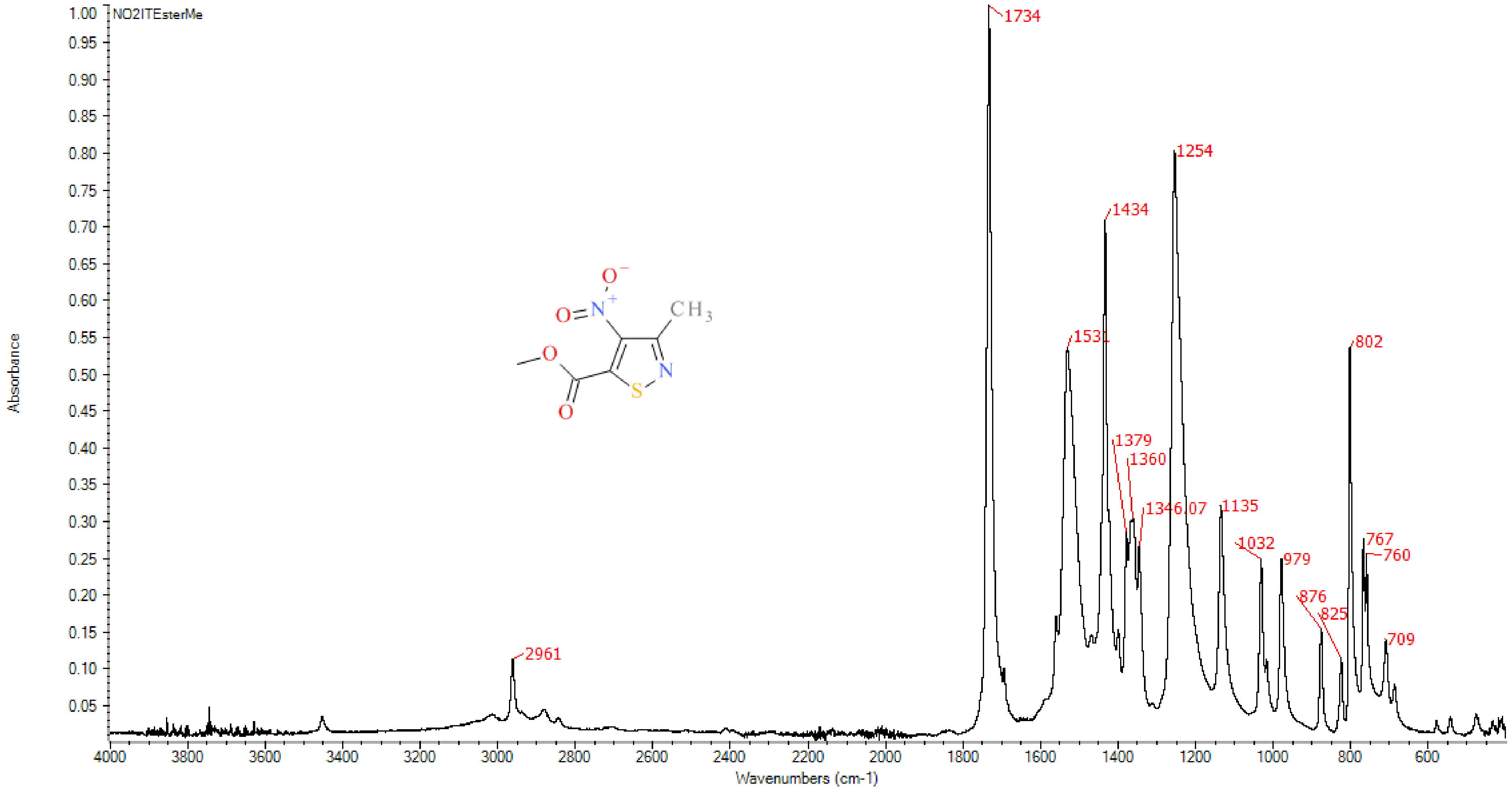

Figure S26. MIR-ATR (Medium Infrared) spectrum of methyl 3-methyl-4-nitro-5-isothiazolecarboxylate (**L2**).

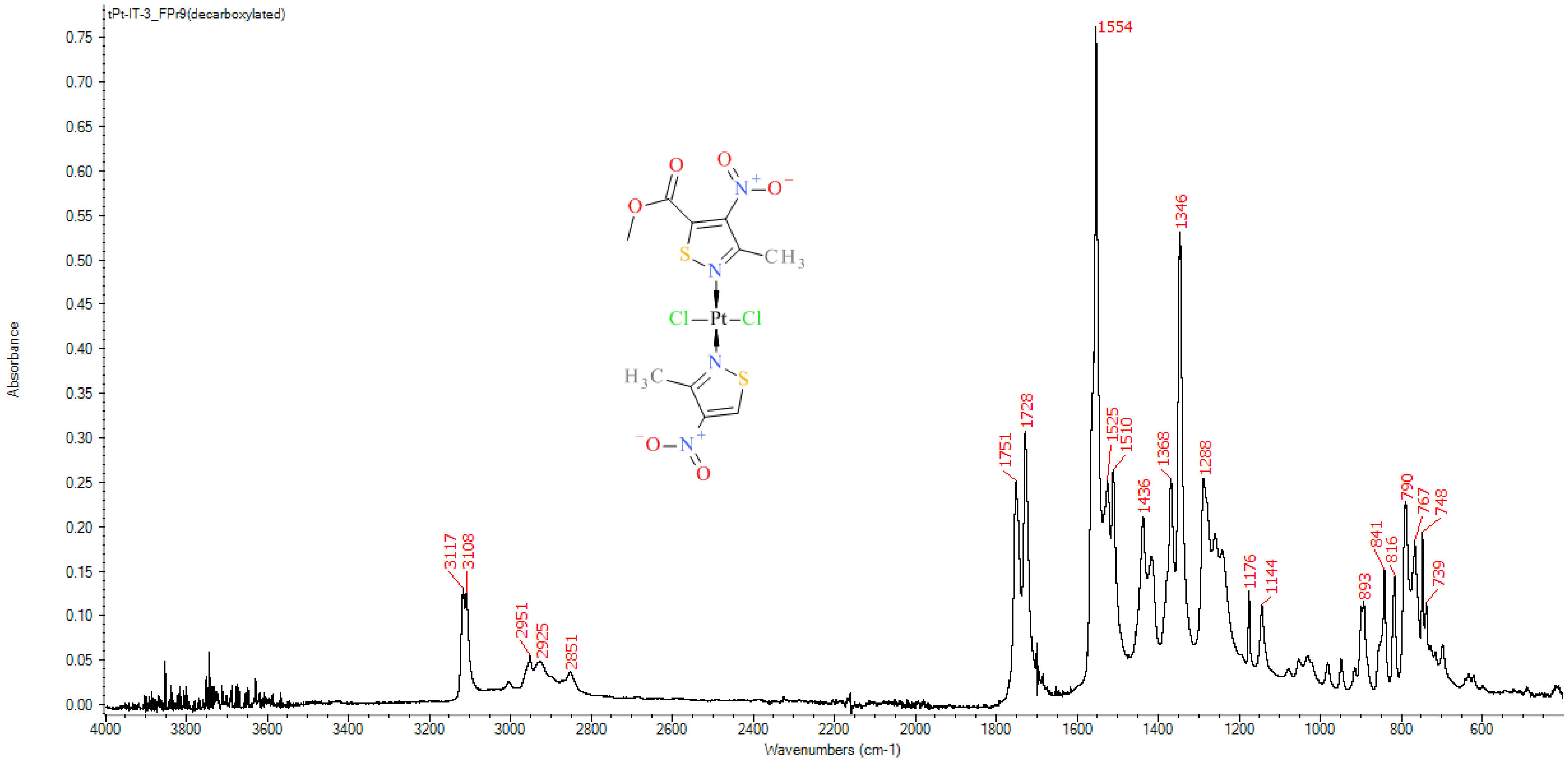

Figure S27 MIR-ATR (Medium Infrared) spectrum of *trans*-dichloro-3-methyl-4-nitroisothiazole 3-methyl-4-nitro-5-(methoxycarbonyl)isothiazole platinum(II) (**C3**).

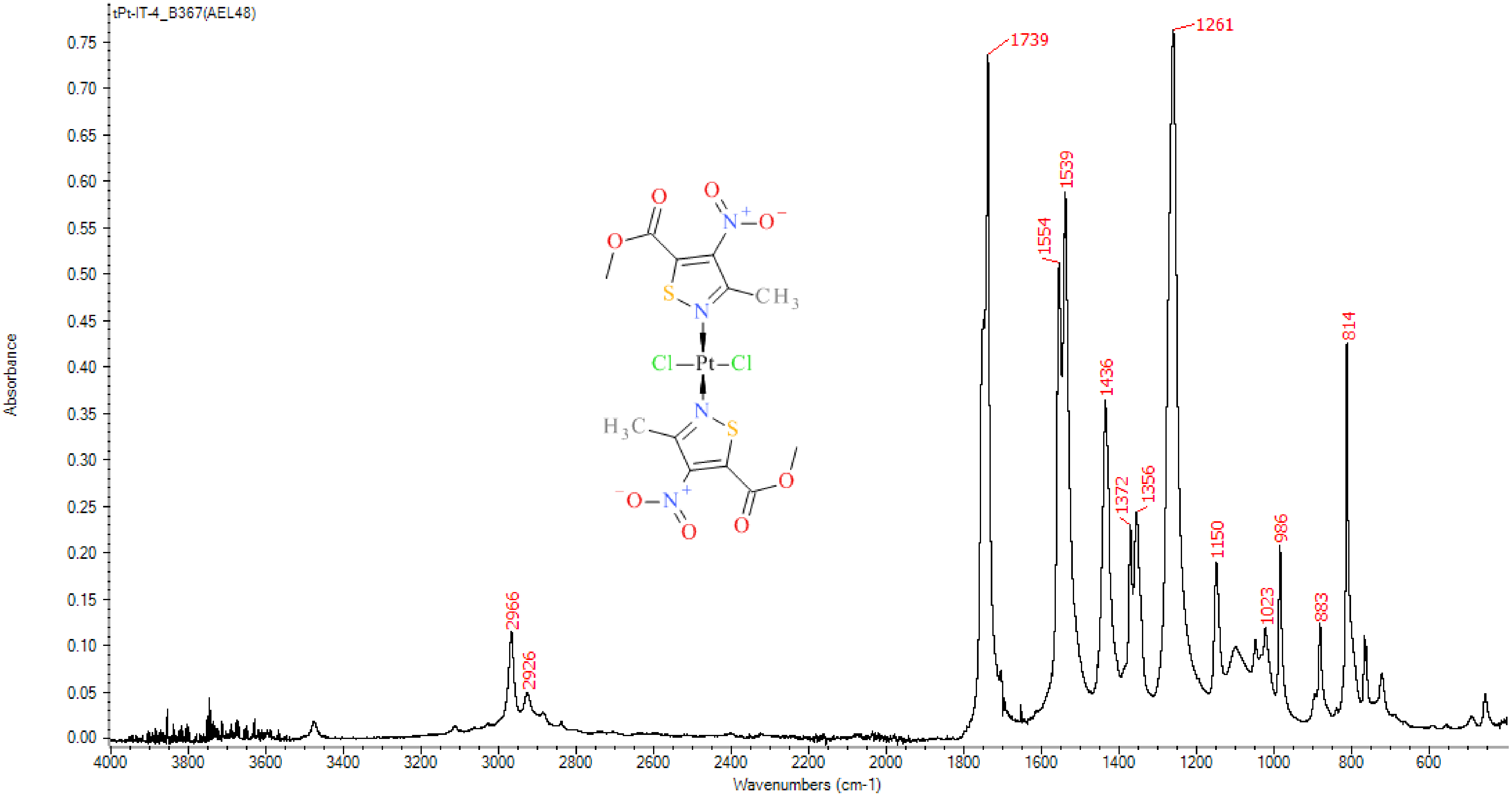

Figure S28. MIR-ATR (Medium Infrared) spectrum of *trans*-dichlorobis(3-methyl-4-nitro-5-(methoxycarbonyl)isothiazole)platinum(II) (C**4**).

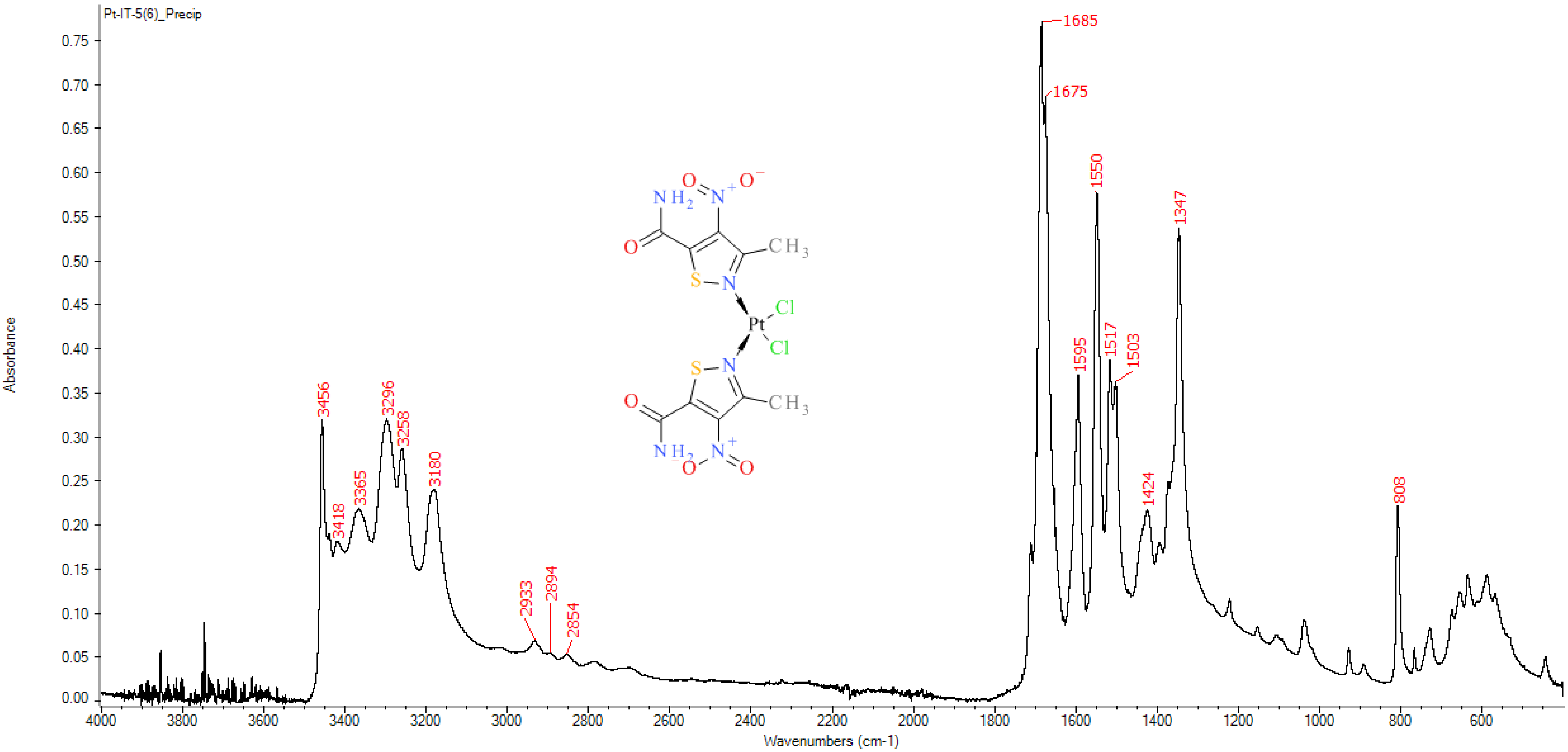

Figure S29. MIR-ATR (Medium Infra red) spectrum of *cis*-dichlorobis(3-methyl-4-nitroisothiazole-5-carboxamide)platinum(II) (C**5**).

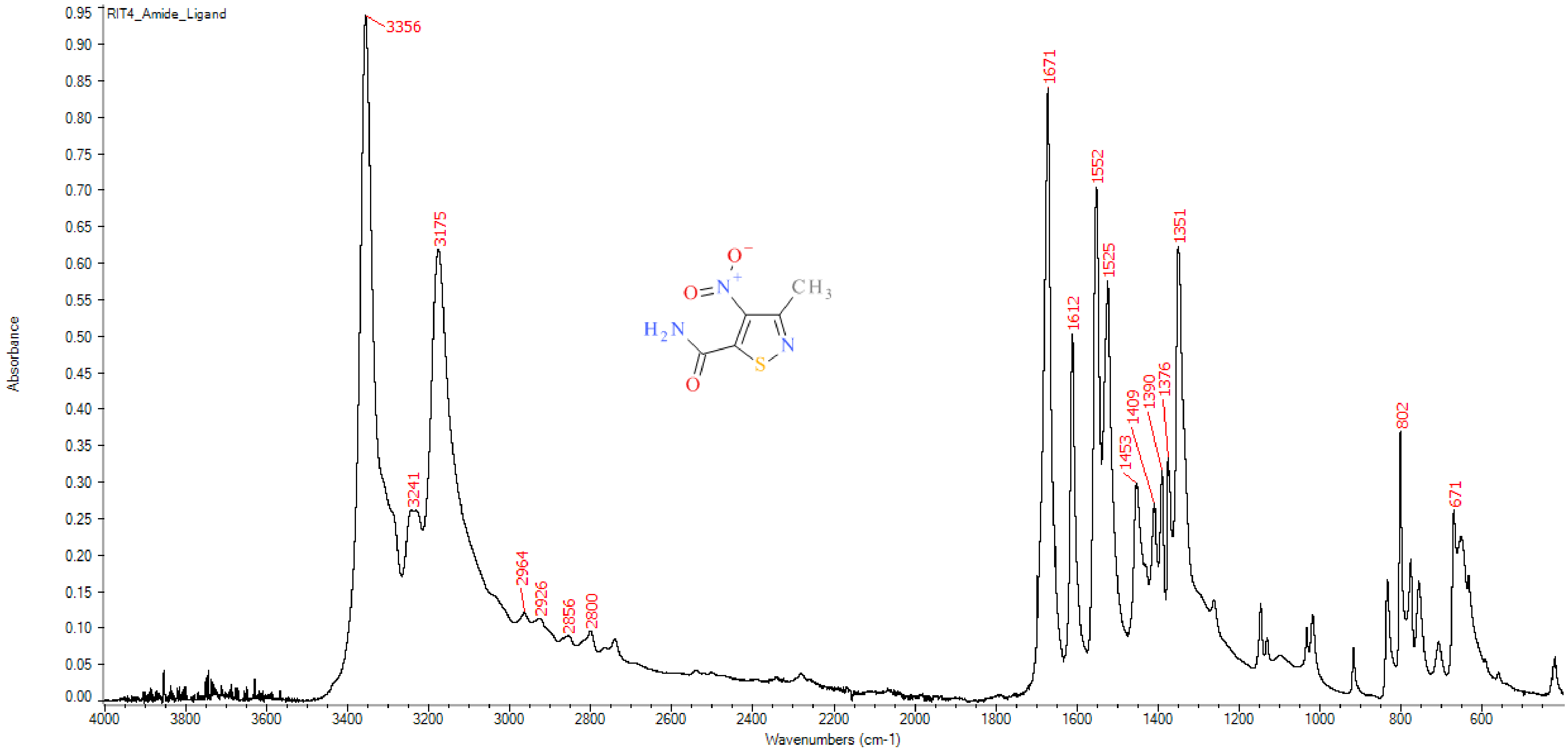

Figure S30. MIR-ATR (Medium Infrared) spectrum of 3-methyl-4-nitroisothiazole-5-carboxamide (**L4**).

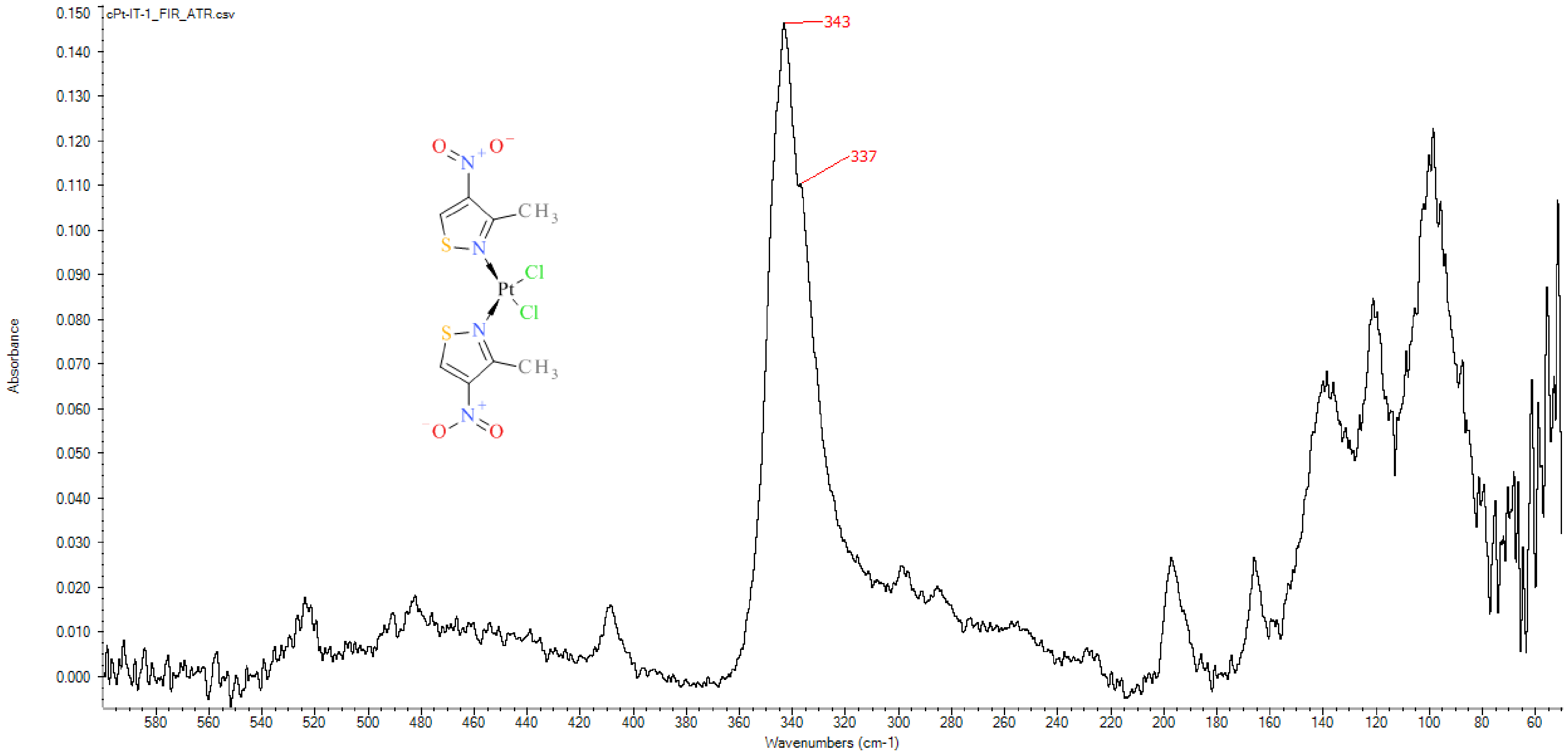

Figure S31. Far IR-ATR spectrum of *cis*-dichlorobis(3-methyl-4-nitroisothiazole)platinum(II) (**C1**), ν[cm^{-1}]: 343 (ν_sCl-Pt), 337 (ν_{as}Cl-Pt).

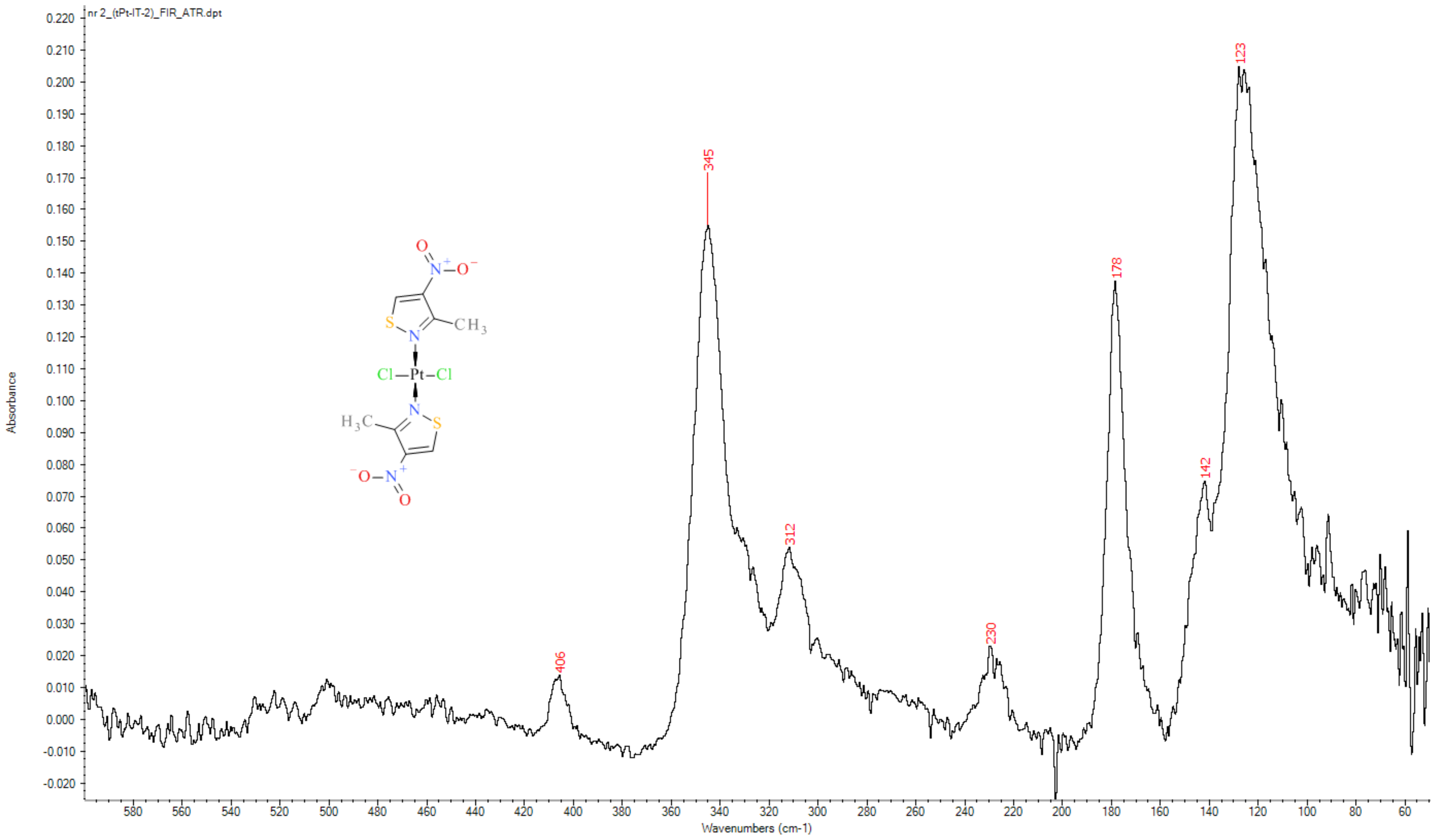

Figure S32. Far IR-ATR spectrum of *trans*-dichlorobis(3-methyl-4-nitroisothiazole)platinum(II) (**C2**).

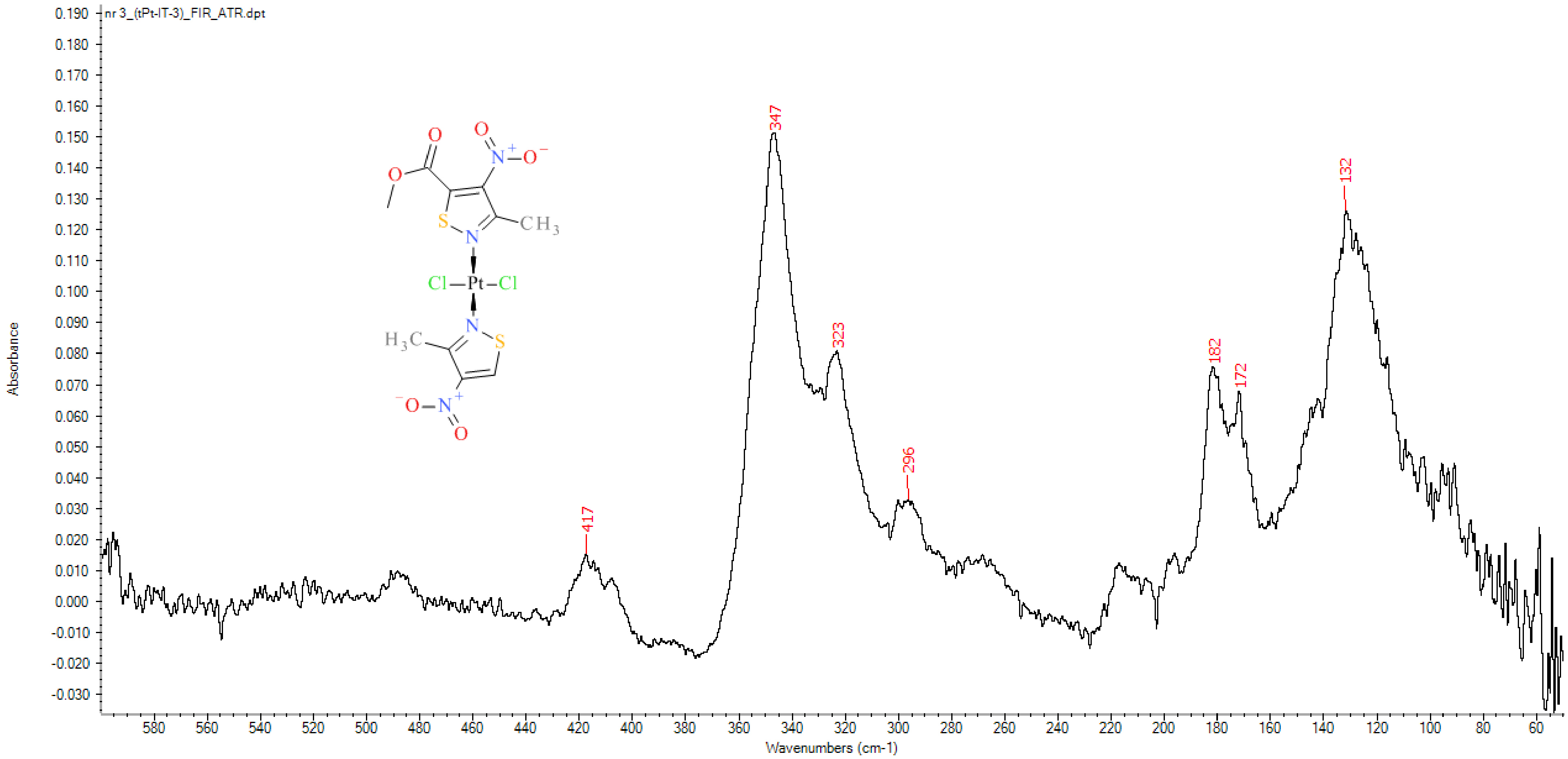

Figure S33. Far IR-ATR spectrum of *trans*-dichloro-3-methyl-4-nitroisothiazole 3-methyl-4-nitro-5-(methoxycarbonyl)isothiazole platinum(II) (**C3**).

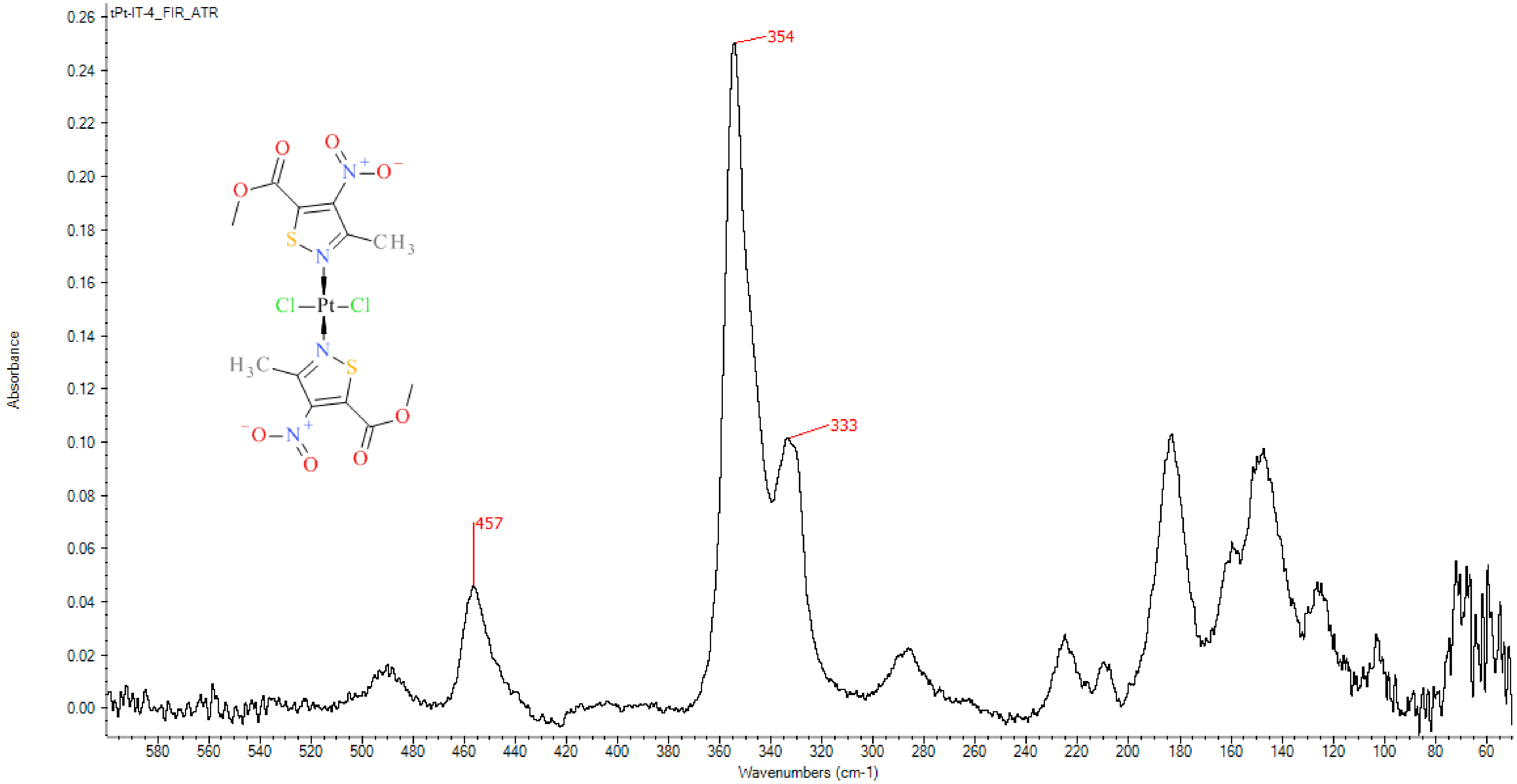

Figure S34. Far IR-ATR spectrum of *trans*-dichlorobis(3-methyl-4-nitro-5-(methoxycarbonyl)isothiazole)platinum(II) (**C4**).

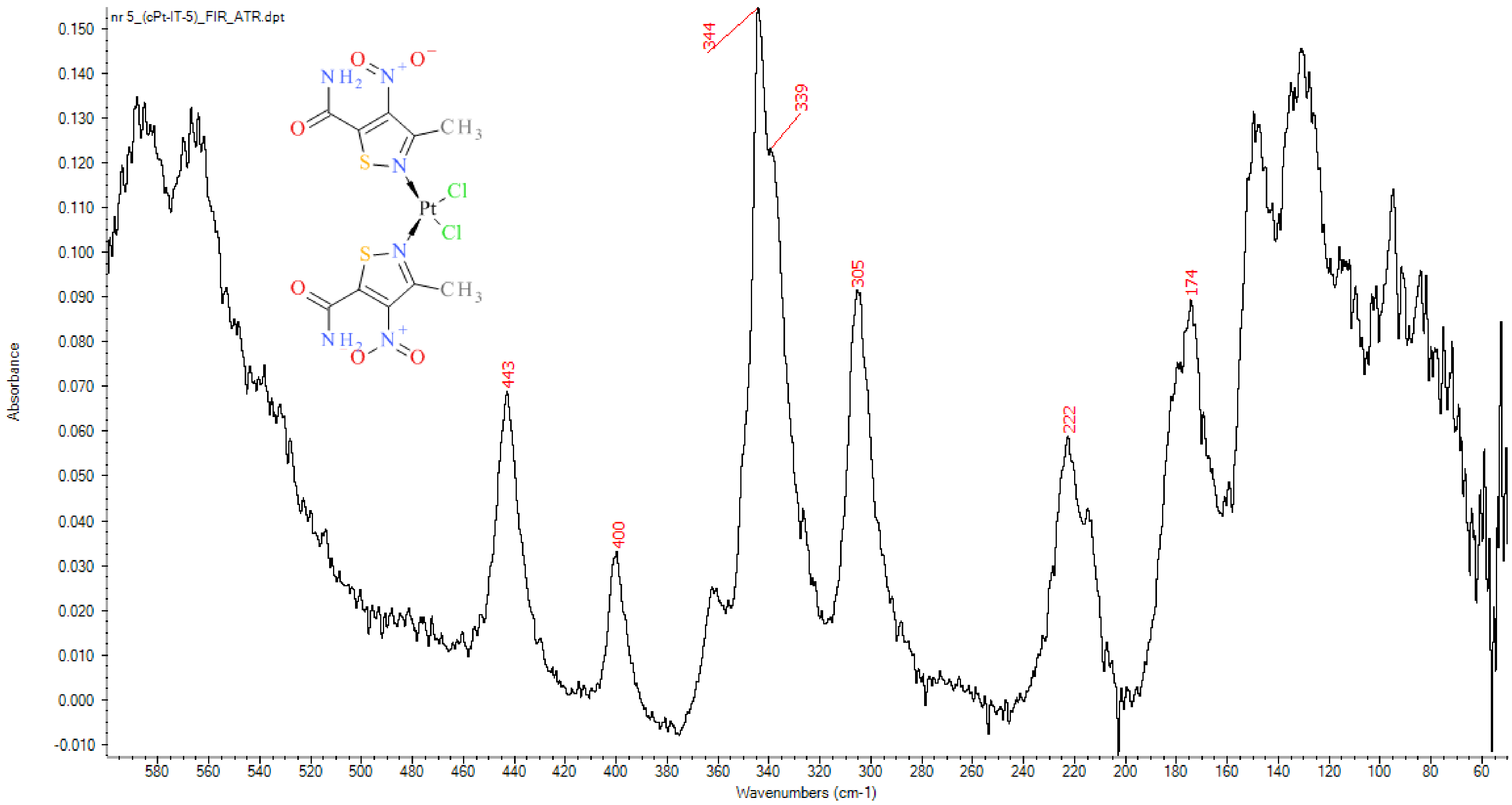

Figure S35. Far IR-ATR spectrum of dichlorobis(3-methyl-4-nitroisothiazole-5-carboxamide)platinum(II) (**C5**).

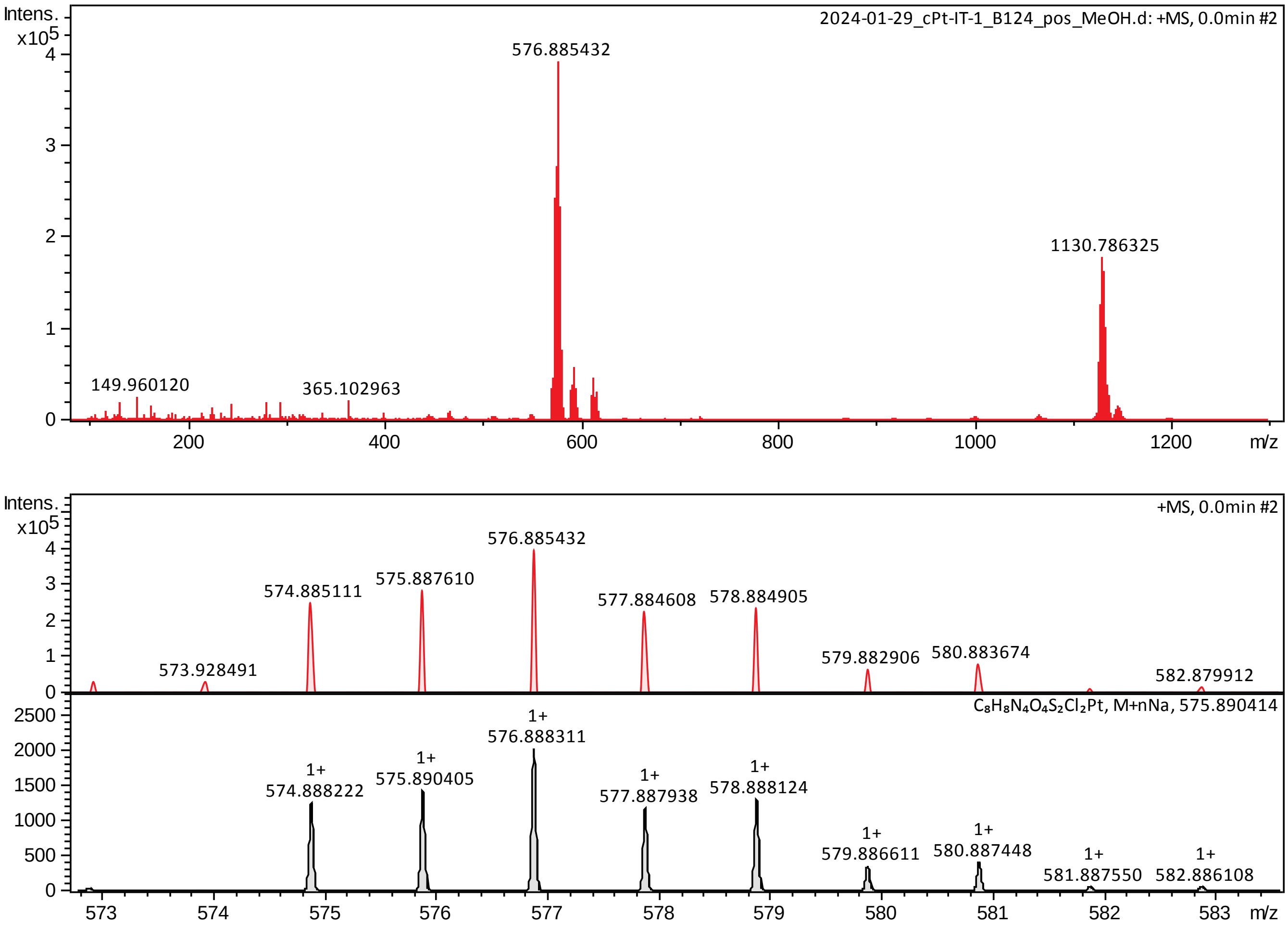

Figure S36. Experimental ESI-MS (positive ionization in MeOH) spectrum (top drawing) of the complex **C1** (*cis*-dichlorobis(3-methyl-4-nitroisothiazole)platinum(II) (**C1**)). Experimental (middle drawing) and simulated (bottom drawing) quasi molecular ion (m/z=**575.887610** u/e (70.9%)) and its isotope peaks for formula $[C_{10}H_{10}N_4O_6S_2Cl_2Pt + Na^+]^+$ (calculated mass of quasi molecular ion: **575.890405**) in ESI-MS spectrum (positive ionization).

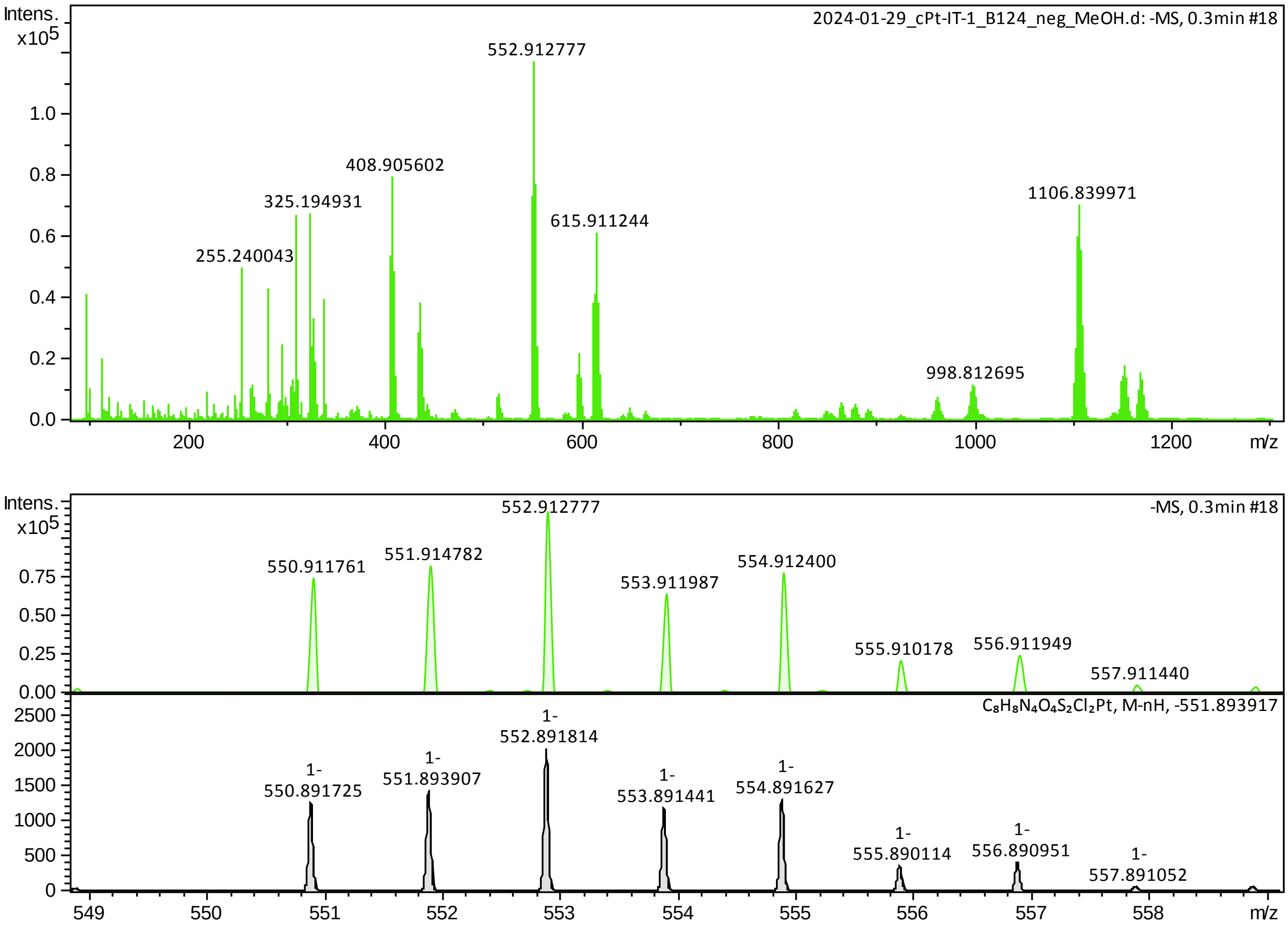

Figure S37. Experimental ESI-MS (negative ionization in MeOH) spectrum (top drawing) of the complex **C1** (*cis*-dichlorobis(3-methyl-4-nitroisothiazole)platinum(II) (**C1**)). Experimental (middle drawing) and simulated (bottom drawing) quasi molecular ion (m/z=**551.914782** u/e (69.3%)) and its isotope peaks for formula $[C_{10}H_{10}N_6O_6S_2Cl_2Pt\text{-}H^+]^-$ (calculated mass: **551.893907)** in ESI-MS spectrum (negative ionization).

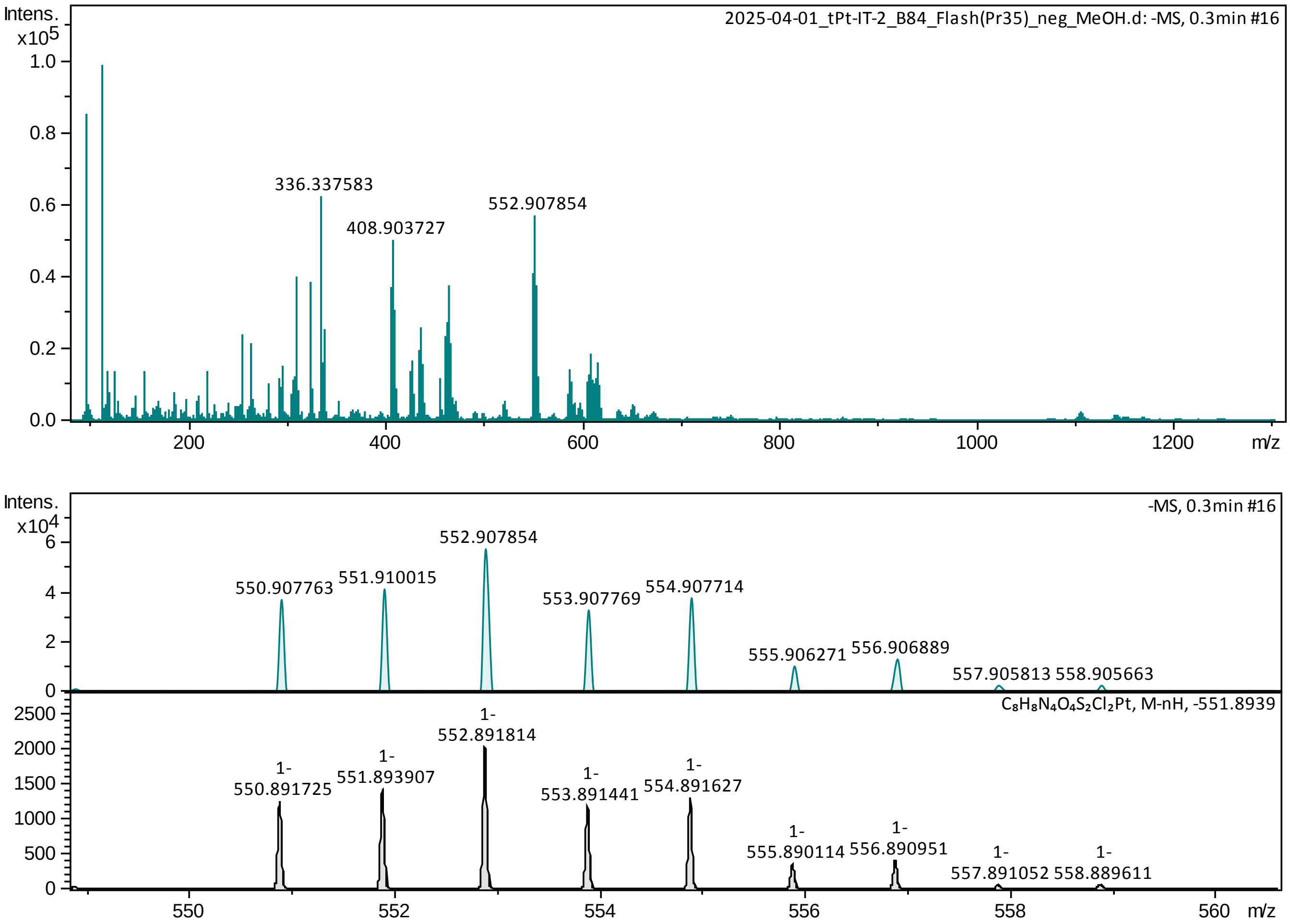

Figure S38. Experimental ESI-MS (negative ionization in MeOH) spectrum (top drawing) of the complex **C2** (*trans*-dichlorobis(3-methyl-4-nitroisothiazole)platinum(II) (**C2**)). Experimental (middle drawing) and simulated (bottom drawing) quasi molecular ion (m/z=**551.910015** u/e (41.2%)) and its isotope peaks for formula $[C_{10}H_{10}N_6O_6S_2Cl_2Pt\text{-}H^+]^-$ (calculated mass: **551.893907)** in ESI-MS spectrum (negative ionization).

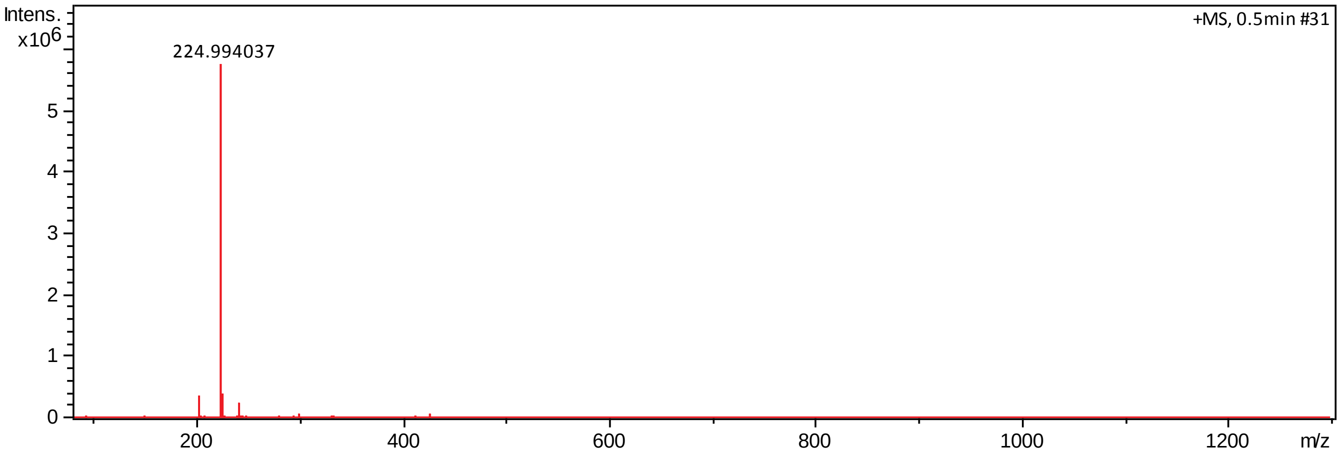

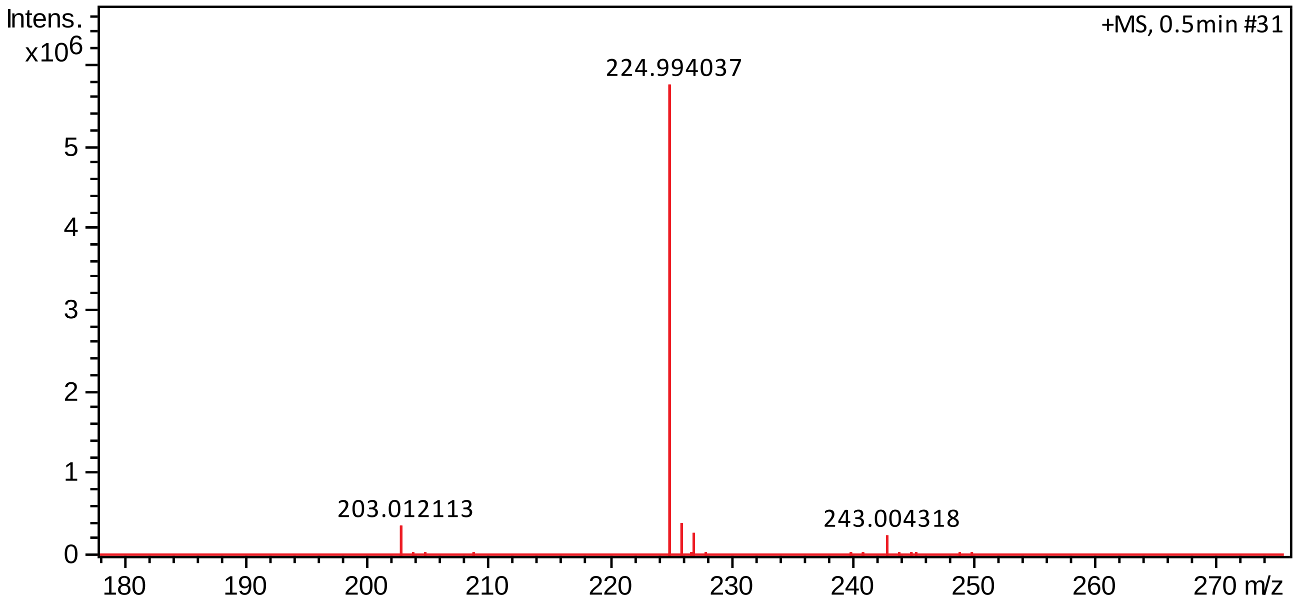

Figure S39. Experimental ESI-MS (positive ionization in MeOH) spectrum of methyl 3-methyl-4-nitro-5-isothiazolecarboxylate (**L2**). Quasi molecular ion peaks for formula $[C_6H_6N_2O_4S+H^+]^+$ (m/z=**203.012113** u/e (6.2%)) and for formula $[C_6H_6N_2O_4S+ Na^+]^+$ (m/z=**224.994037** u/e (100%)). Top-whole spectrum, bottom-molecular peak range.

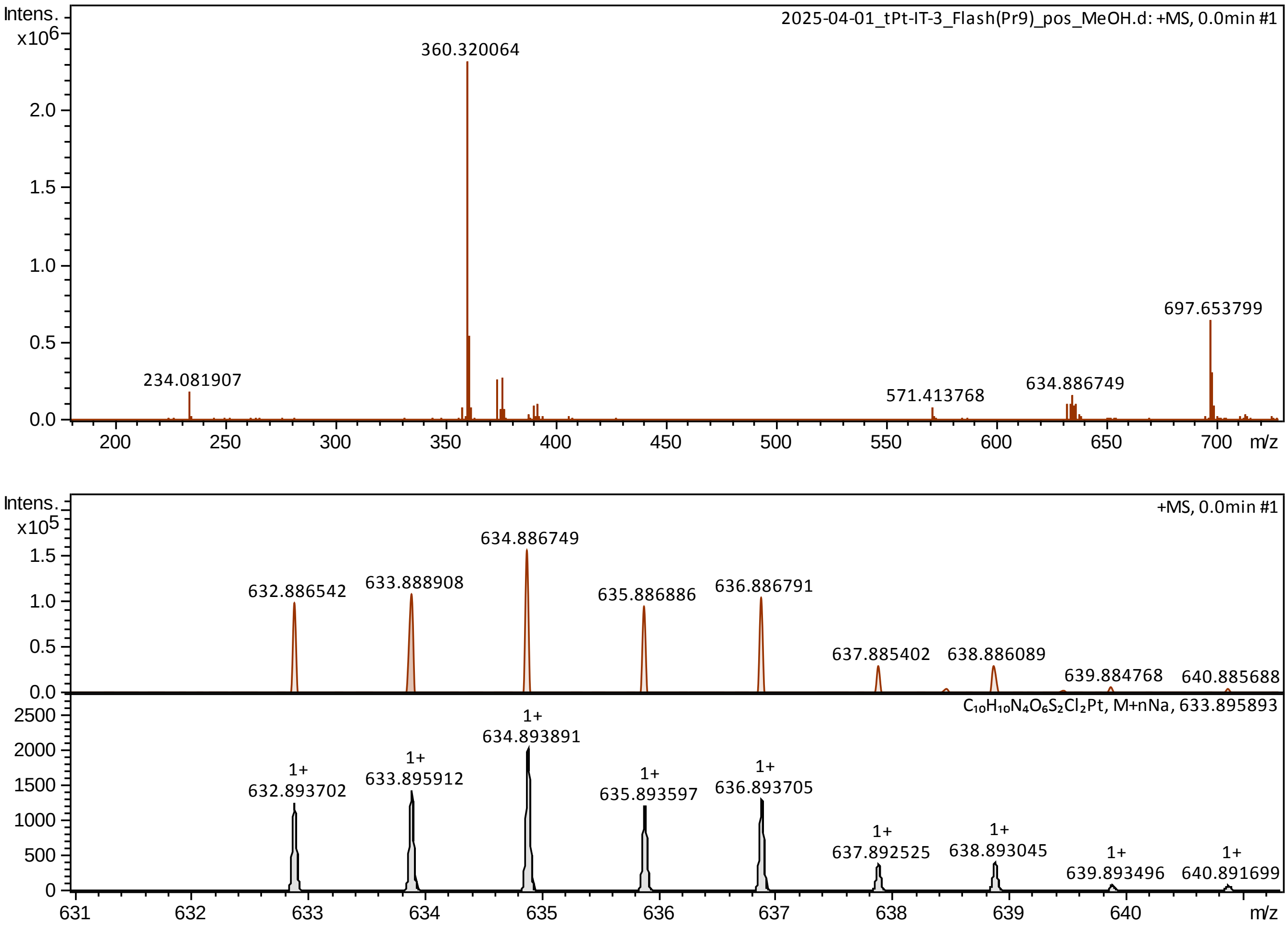

Figure S40. Experimental ESI-MS (positive ionization in MeOH) spectrum (top drawing) of the complex **C3 (***trans* dichloro-3-methyl-4-nitroisothiazole 3-methyl-4-nitro-5-(methoxycarbonyl)isothiazole platinum(II) (**C3**)). Experimental (middle drawing) and simulated (bottom drawing) quasi molecular ion (m/z=**633.888908** u/e (4.6%)) and its isotope peaks for formula $[C_{10}H_{10}N_4O_6S_2Cl_2Pt +Na^+]^+$ (calculated mass: **633.895912**) in ESI-MS spectrum (positive ionization).

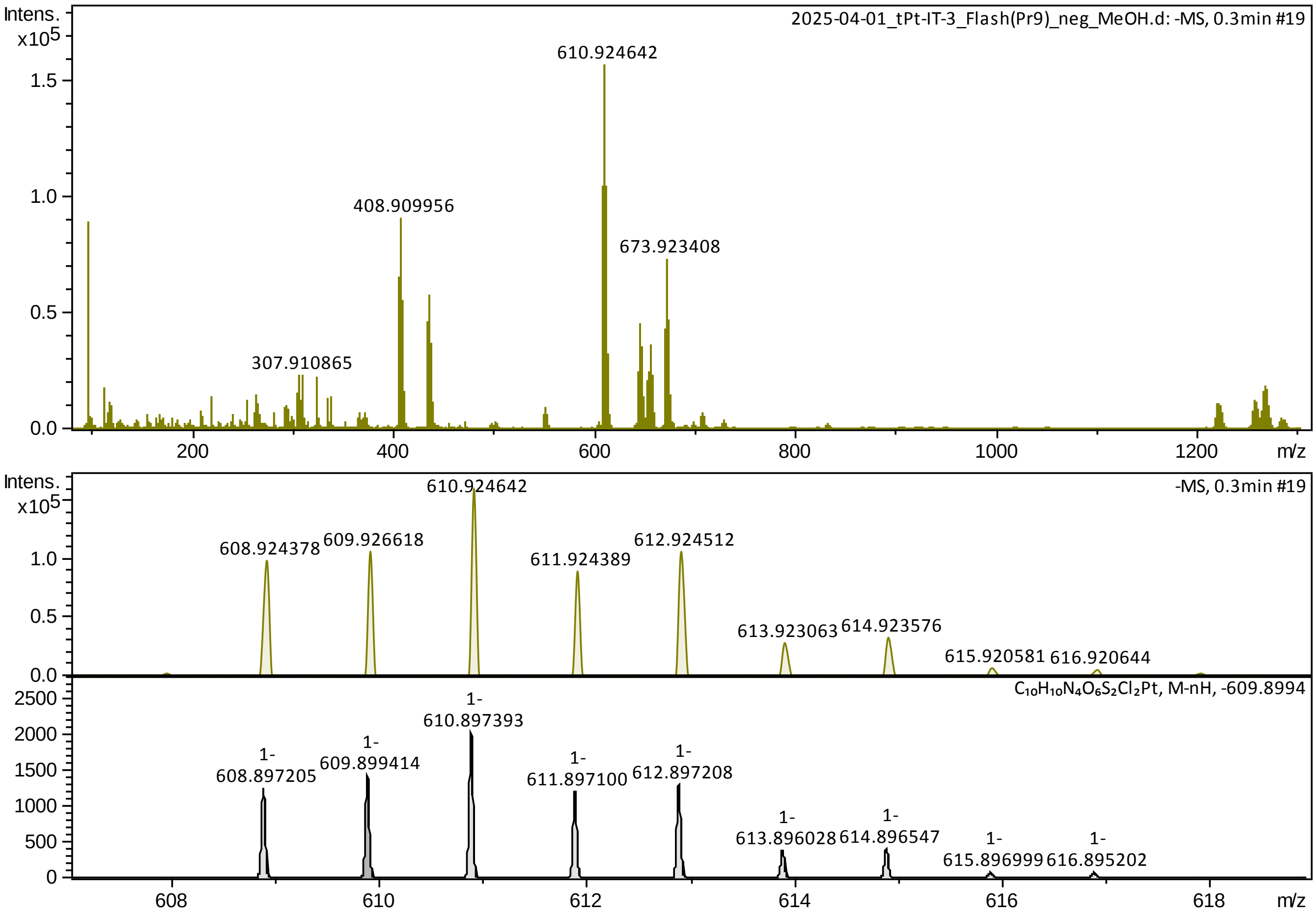

Figure S41. Experimental ESI-MS (negative ionization in MeOH) spectrum (top drawing) of the complex **C3** (*trans* dichloro-3-methyl-4-nitroisothiazole 3-methyl-4-nitro-5-(methoxycarbonyl)isothiazole platinum(II) (**C3**)). Experimental (middle drawing) and simulated (bottom drawing) quasi molecular ion (m/z=**609.926618** u/e (66.5%)) and its isotope peaks for formula $[C_{10}H_{10}N_6O_6S_2Cl_2Pt\text{-}H^+]^-$ (calculated mass: **609.899414)** in ESI-MS spectrum (negative ionization).

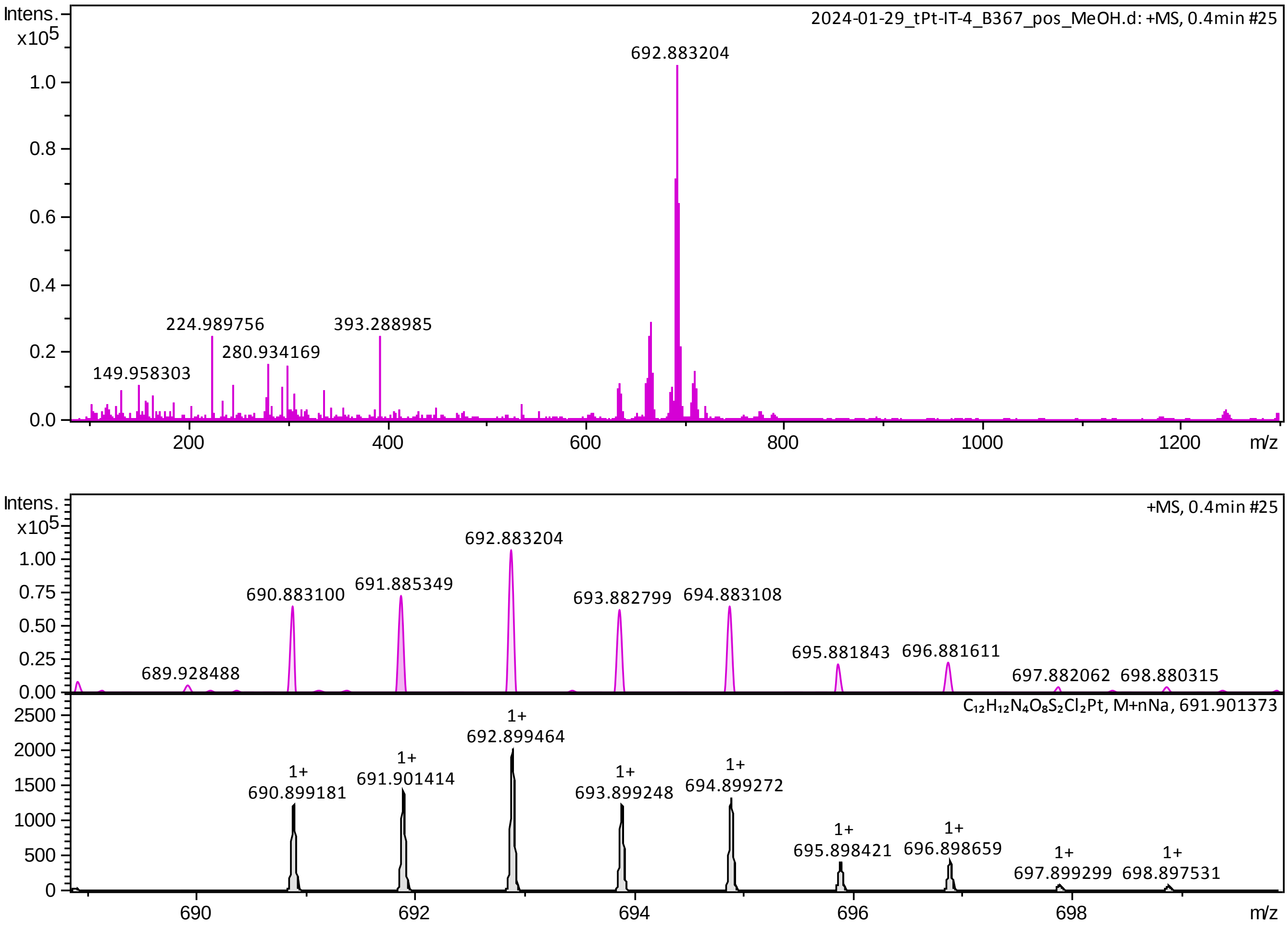

Figure S42. Experimental ESI-MS (positive ionization in MeOH) spectrum (top drawing) of the complex **C4 (***trans* dichlorobis(3-methyl-4-nitro-5-(methoxycarbonyl)isothiazole)platinum(II) (**C4**)). Experimental (middle drawing) and simulated (bottom drawing) quasi molecular ion (m/z=**691.885349** u/e (67.9%)) and its isotope peaks for formula $[C_{12}H_{12}N_4O_8S_2Cl_2Pt + Na^+]^+$ (calculated mass: **691.901414**) in ESI-MS spectrum (positive ionization).

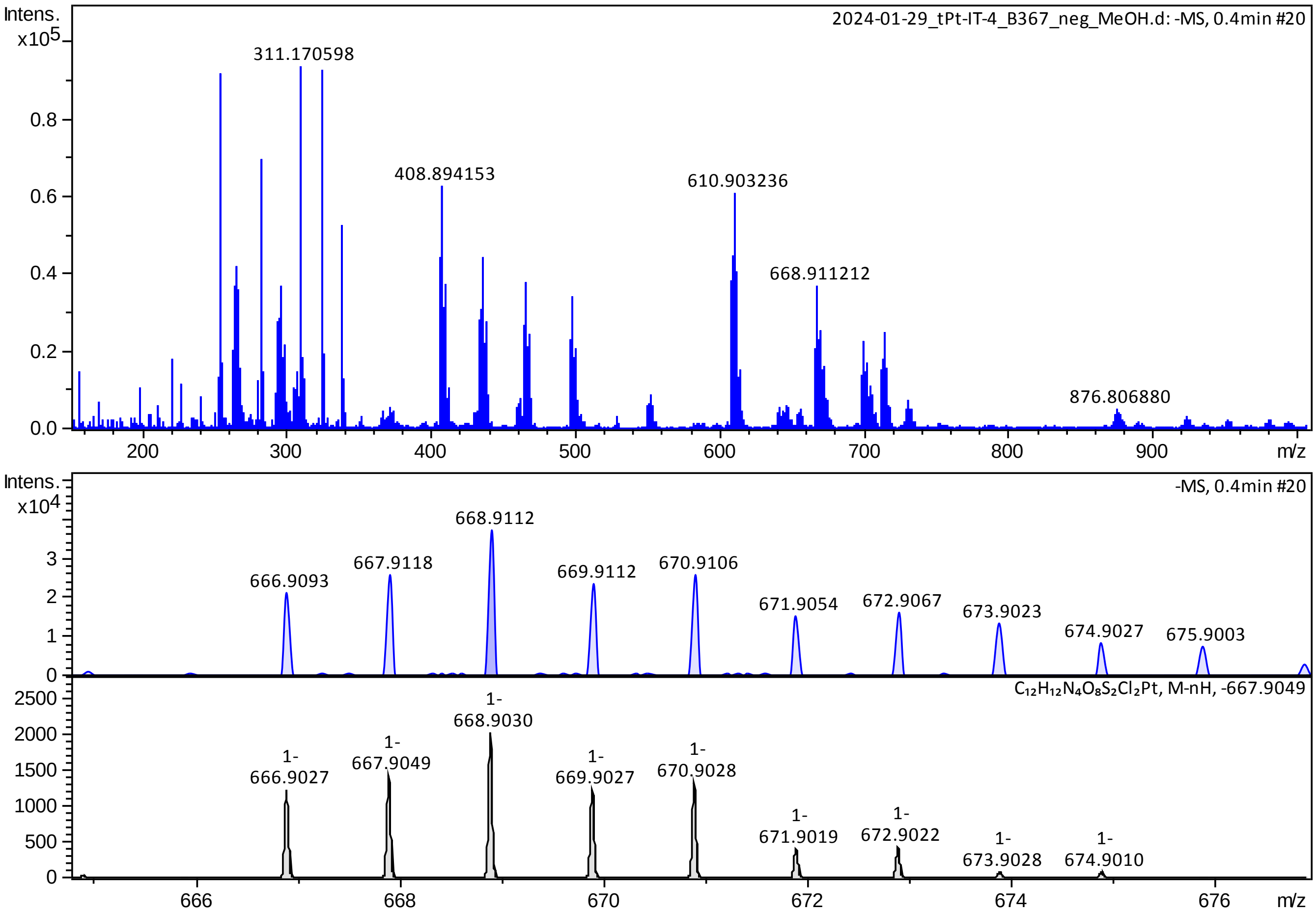

Figure S43. Experimental ESI-MS (negative ionization in MeOH) spectrum (top drawing) of the complex **C4 (***trans* dichlorobis(3-methyl-4-nitro-5-(methoxycarbonyl)isothiazole)platinum(II) (**C4**)). Experimental (middle drawing) and simulated (bottom drawing) quasi molecular ion (m/z=**667.911813** u/e (27.2%)) and its isotope peaks for formula $[C_{10}H_{10}N_6O_6S_2Cl_2Pt\text{-}H^+]^-$ (calculated mass: **667.904917)** in ESI-MS spectrum (negative ionization).

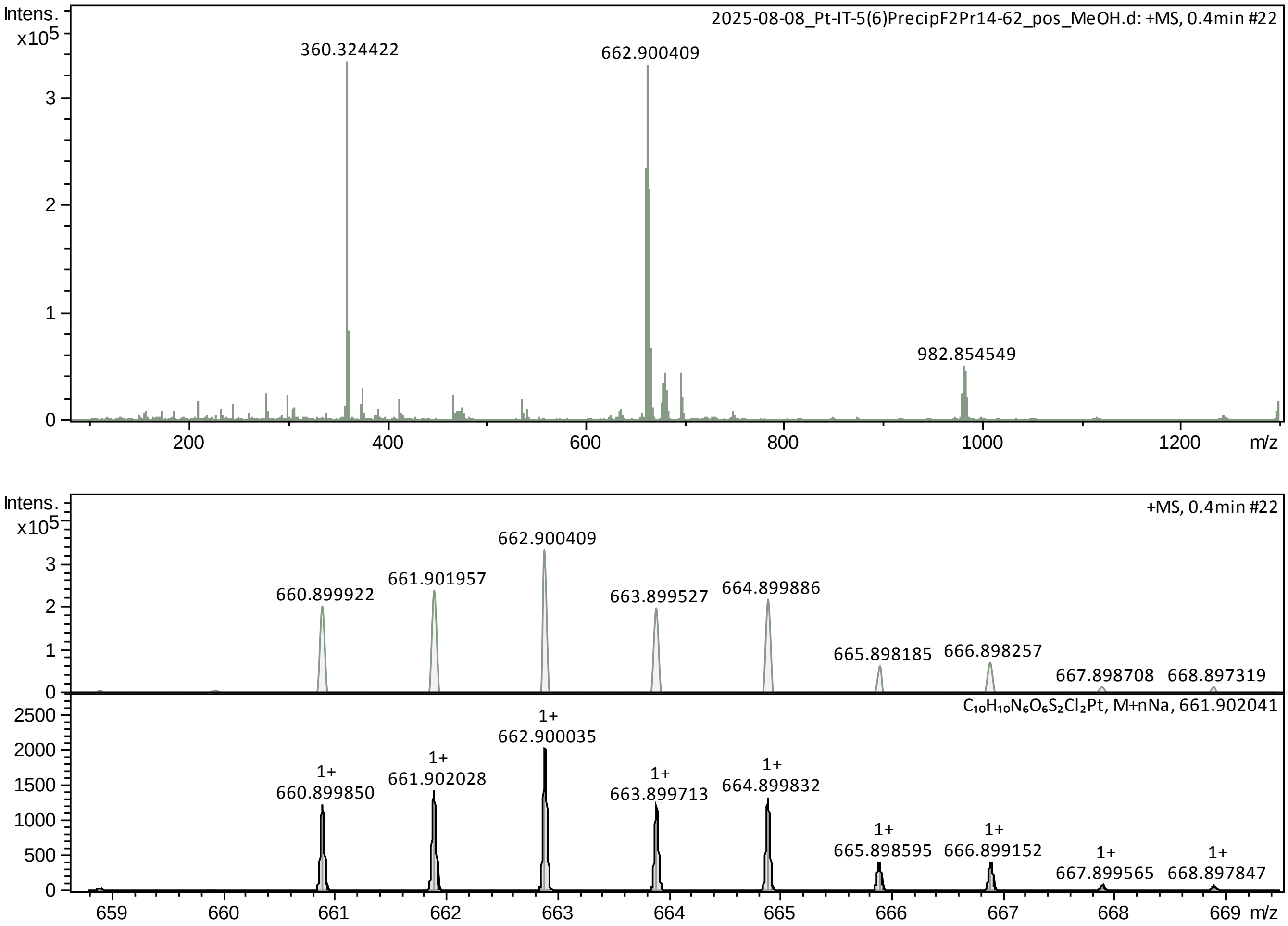

Figure S44. Experimental ESI-MS (positive ionization in MeOH) spectrum (top drawing) of the complex **C5** (*cis*-dichlorobis(3-methyl-4-nitroisothiazole-5-carboxamide)platinum(II) (**C5**)). Experimental (middle drawing) and simulated (bottom drawing) quasi molecular ion (m/z=**661.901957** u/e(70.3%)) and its isotope peaks for formula $[C_{10}H_{10}N_6O_6S_2Cl_2Pt+Na^+]^+$ (calculated mass: **661.902028**) in ESI-MS spectrum (positive ionization).

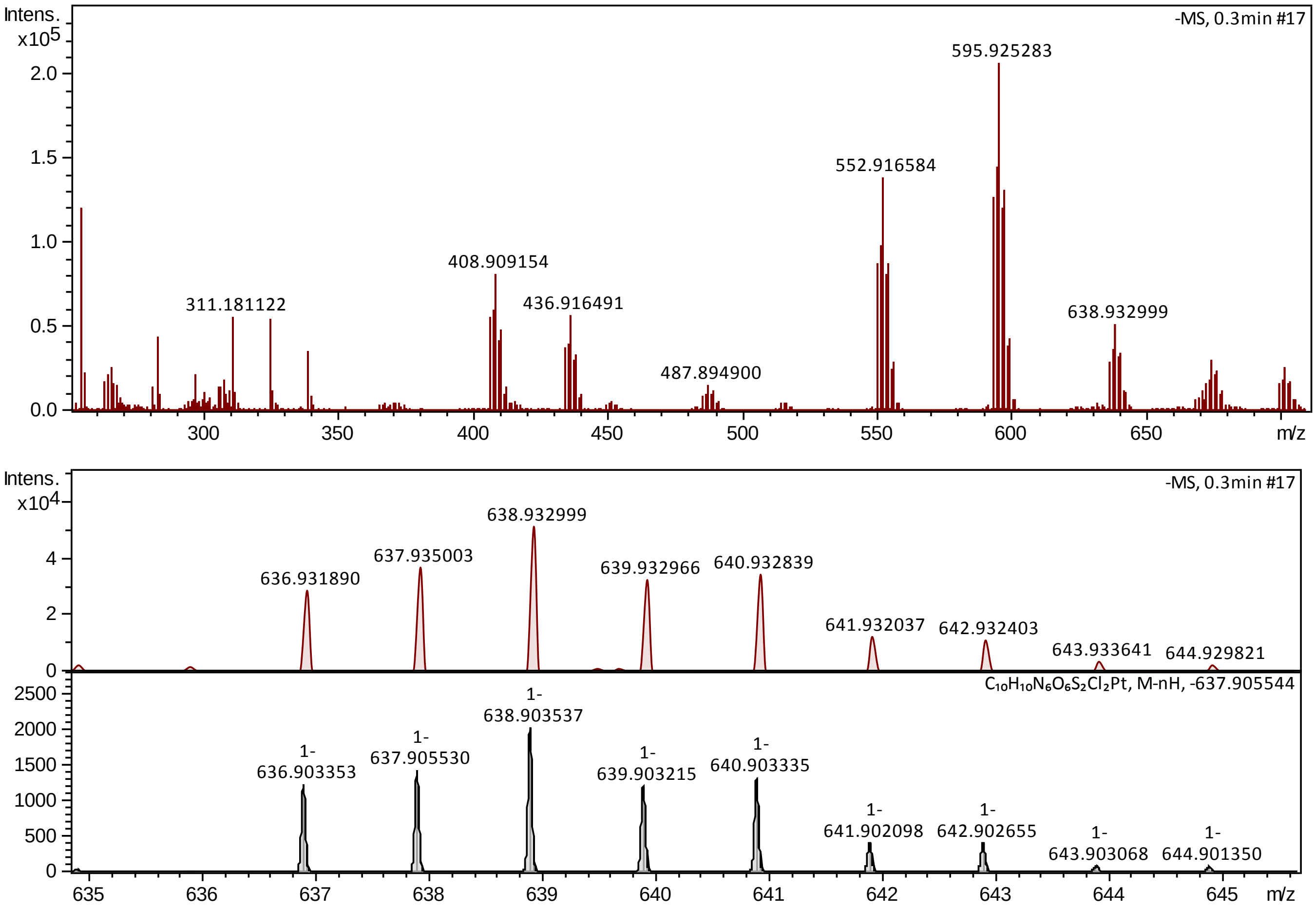

Figure S45. Experimental ESI-MS (negative ionization in MeOH) spectrum (top drawing) of the complex **C5** (*cis*-dichlorobis(3-methyl-4-nitroisothiazole-5-carboxamide)platinum(II) (**C5**)). Experimental (middle drawing) and simulated (bottom drawing) quasi molecular ion (m/z=**637.935003** u/e (17.55%)) and its isotope peaks for formula $[C_{10}H_{10}N_6O_6S_2Cl_2Pt\text{-}H^+]^-$ (calculated mass: **637.905530)** in ESI-MS spectrum (negative ionization).

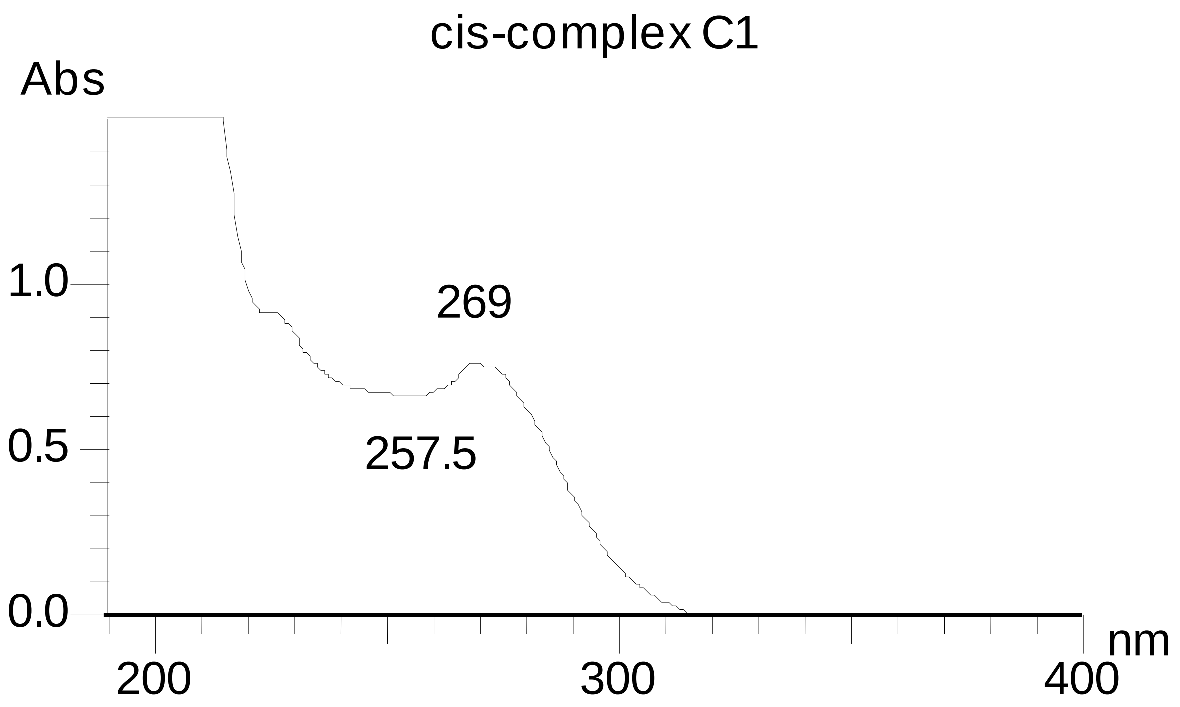

Figures S46. UV spectrum of *cis*-complex **C1**, c=2·10^{-5} M

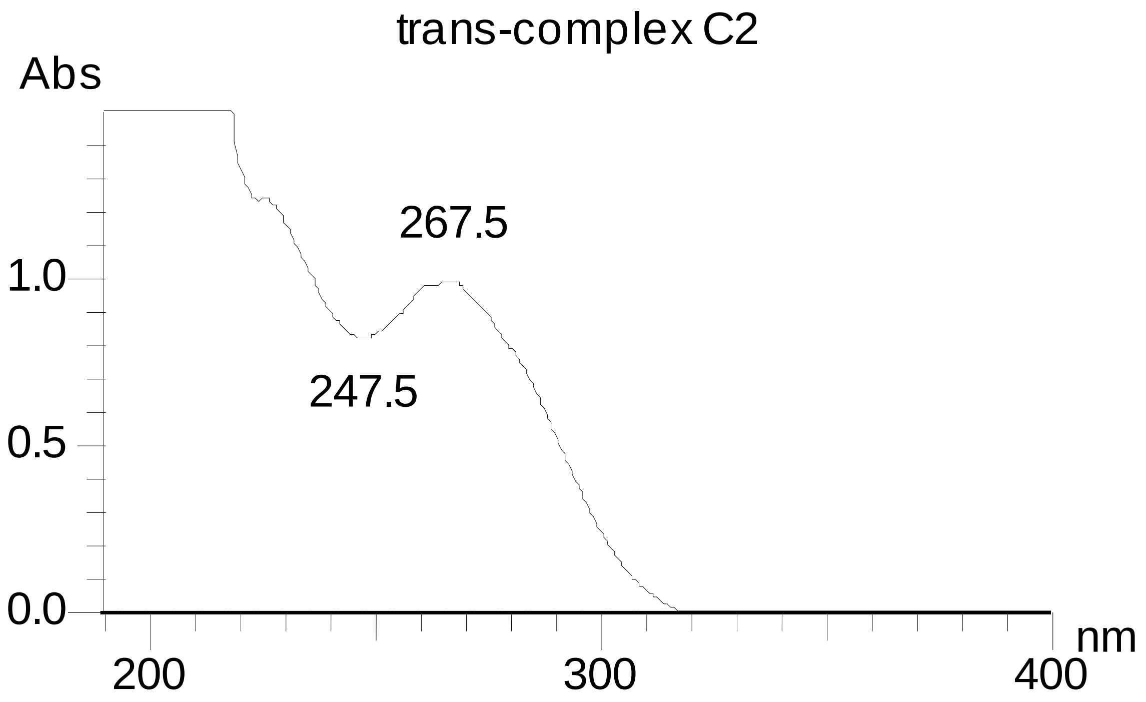

Figures S47. UV spectrum of *cis*-complex **C2**, c=3·10^{-5} M

Table S1. Crystal data and structure refinement for methyl 3-methyl-4-nitro-5-isothiazolecarboxylate (**L2**).

Identification code	ak987ak	
Empirical formula	$C_6 H_6 N_2 O_4 S$	
Formula weight	202.19	
Temperature	110(2) K	
Wavelength	0.71073 Å	
Crystal system	Monoclinic	
Space group	I2/a	
Unit cell dimensions	a = 13.791(2) Å	
	b = 8.414(2) Å	β= 104.36(3)°
	c = 14.994(2) Å	
Volume	1685.5(6) $Å^3$	
Z	8	
Density (calculated)	1.594 Mg/m^3	
Absorption coefficient	0.367 mm^{-1}	
F(000)	832	
Crystal size	0.150 x 0.150 x 0.100 mm^3	
Theta range for data collection	2.798 to 28.880°.	
Index ranges	-18<=h<=18, -11<=k<=11, -20<=l<=19	
Reflections collected	12407	
Independent reflections	2088 [R(int) = 0.0364]	
Completeness to theta = 25.000°	100.0 %	
Absorption correction	Analytical	
Max. and min. transmission	0.890 and 0.765	
Refinement method	Full-matrix least-squares on F^2	
Data / restraints / parameters	2088 / 0 / 120	
Goodness-of-fit on F^2	1.050	
Final R indices [I>2sigma(I)]	R_1 = 0.0401, wR_2 = 0.0881	
R indices (all data)	R_1 = 0.0561, wR_2 = 0.0946	
Largest diff. peak and hole	0.354 and -0.287 e.$Å^{-3}$	

Table S2. Atomic coordinates (x 10^4) and equivalent isotropic displacement parameters (Å^{2}x 10^3) for methyl 3-methyl-4-nitro-5-isothiazolecarboxylate (**L2**). U(eq) is defined as one third of the trace of the orthogonalized U^{ij} tensor.

	x	y	z	U(eq)
S(1)	-1613(1)	4953(1)	-3829(1)	21(1)
O(1)	-1501(1)	1549(1)	-4178(1)	19(1)
O(2)	-2795(1)	798(2)	-3585(1)	23(1)
O(3)	-4405(1)	3343(2)	-2853(1)	32(1)
O(4)	-4765(1)	2978(2)	-4319(1)	32(1)
N(1)	-2324(1)	6460(2)	-3685(1)	21(1)
N(2)	-4222(1)	3457(2)	-3603(1)	17(1)
C(3)	-3203(1)	5959(2)	-3593(1)	17(1)
C(4)	-3300(1)	4278(2)	-3651(1)	15(1)
C(5)	-2472(1)	3525(2)	-3781(1)	16(1)
C(6)	-2292(1)	1791(2)	-3832(1)	17(1)
C(7)	-3980(1)	7099(2)	-3453(1)	21(1)
C(8)	-1153(2)	-84(2)	-4189(2)	26(1)

Table S3. Selected bond lengths [Å] and angles [°] for methyl 3-methyl-4-nitro-5-isothiazolecarboxylate (**L2**).

S(1)-N(1)	1.6498(16)
S(1)-C(5)	1.7012(17)
O(1)-C(6)	1.333(2)
O(1)-C(8)	1.458(2)
O(2)-C(6)	1.201(2)
O(3)-N(2)	1.2157(19)
O(4)-N(2)	1.216(2)
N(1)-C(3)	1.323(2)
N(2)-C(4)	1.464(2)
C(3)-C(4)	1.421(2)
C(3)-C(7)	1.492(2)
C(4)-C(5)	1.362(2)
C(5)-C(6)	1.485(2)
N(1)-S(1)-C(5)	95.44(8)
C(6)-O(1)-C(8)	116.88(13)
C(3)-N(1)-S(1)	110.99(12)
O(4)-N(2)-O(3)	124.52(15)
O(4)-N(2)-C(4)	117.76(14)
O(3)-N(2)-C(4)	117.68(15)
N(1)-C(3)-C(4)	112.73(15)
N(1)-C(3)-C(7)	121.26(15)
C(4)-C(3)-C(7)	126.01(15)
C(5)-C(4)-C(3)	113.69(14)
C(5)-C(4)-N(2)	123.81(15)
C(3)-C(4)-N(2)	122.48(14)
C(4)-C(5)-C(6)	128.43(15)
C(4)-C(5)-S(1)	107.15(12)
C(6)-C(5)-S(1)	124.36(13)
O(2)-C(6)-O(1)	127.15(16)
O(2)-C(6)-C(5)	123.37(16)
O(1)-C(6)-C(5)	109.47(14)

Table S4. Anisotropic displacement parameters ($Å^2 x 10^3$) for methyl 3-methyl-4-nitro-5-isothiazolecarboxylate (**L2**). The anisotropic displacement factor exponent takes the form: $-2\pi^2[h^2 a^{*2}U^{11} + ... + 2 h k a^* b^* U^{12}]$

	U^{11}	U^{22}	U^{33}	U^{23}	U^{13}	U^{12}
S(1)	16(1)	17(1)	34(1)	-3(1)	11(1)	-3(1)
O(1)	20(1)	15(1)	25(1)	1(1)	10(1)	3(1)
O(2)	26(1)	18(1)	29(1)	2(1)	12(1)	-2(1)
O(3)	25(1)	50(1)	23(1)	4(1)	12(1)	-9(1)
O(4)	24(1)	44(1)	29(1)	-12(1)	5(1)	-16(1)
N(1)	19(1)	16(1)	30(1)	-3(1)	9(1)	-2(1)
N(2)	15(1)	15(1)	22(1)	0(1)	7(1)	-1(1)
C(3)	18(1)	17(1)	15(1)	-2(1)	3(1)	-2(1)
C(4)	14(1)	16(1)	15(1)	0(1)	5(1)	-3(1)
C(5)	17(1)	15(1)	15(1)	-1(1)	5(1)	-3(1)
C(6)	18(1)	17(1)	15(1)	-1(1)	5(1)	0(1)
C(7)	22(1)	16(1)	26(1)	-3(1)	8(1)	2(1)
C(8)	31(1)	17(1)	33(1)	3(1)	14(1)	9(1)

Table S5. Hydrogen coordinates (x 10^4) and isotropic displacement parameters ($Å^2 x 10^3$) for methyl 3-methyl-4-nitro-5-isothiazolecarboxylate (**L2**).

	x	y	z	U(eq)
H(7A)	-3731	8188	-3461	31
H(7B)	-4131	6889	-2859	31
H(7C)	-4590	6968	-3948	31
H(8A)	-689	-332	-3596	39
H(8B)	-808	-210	-4683	39
H(8C)	-1726	-809	-4295	39

Table S6. Torsion angles [°] for methyl 3-methyl-4-nitro-5-isothiazolecarboxylate (**L2**).

C(5)-S(1)-N(1)-C(3)	0.40(14)
S(1)-N(1)-C(3)-C(4)	-0.6(2)
S(1)-N(1)-C(3)-C(7)	179.98(14)
N(1)-C(3)-C(4)-C(5)	0.5(2)
C(7)-C(3)-C(4)-C(5)	179.93(17)
N(1)-C(3)-C(4)-N(2)	-177.66(15)
C(7)-C(3)-C(4)-N(2)	1.8(3)
O(4)-N(2)-C(4)-C(5)	-75.4(2)
O(3)-N(2)-C(4)-C(5)	106.7(2)
O(4)-N(2)-C(4)-C(3)	102.6(2)
O(3)-N(2)-C(4)-C(3)	-75.3(2)
C(3)-C(4)-C(5)-C(6)	177.09(17)
N(2)-C(4)-C(5)-C(6)	-4.8(3)
C(3)-C(4)-C(5)-S(1)	-0.19(19)
N(2)-C(4)-C(5)-S(1)	177.94(13)
N(1)-S(1)-C(5)-C(4)	-0.11(14)
N(1)-S(1)-C(5)-C(6)	-177.54(16)
C(8)-O(1)-C(6)-O(2)	-5.5(3)
C(8)-O(1)-C(6)-C(5)	173.84(15)
C(4)-C(5)-C(6)-O(2)	-15.1(3)
S(1)-C(5)-C(6)-O(2)	161.75(15)
C(4)-C(5)-C(6)-O(1)	165.49(17)
S(1)-C(5)-C(6)-O(1)	-17.7(2)

Table S7. Crystal data and structure refinement for *trans*-dichlorobis(3-methyl-4-nitro-5-(methoxycarbonyl)isothiazole)platinum(II) (**C4**).

Identification code	0302ak	
Empirical formula	$C_{12} H_{12} Cl_2 N_4 O_8 Pt S_2$	
Formula weight	670.37	
Temperature	100(2) K	
Wavelength	0.71073 Å	
Crystal system	Monoclinic	
Space group	$P2_1/c$	
Unit cell dimensions	a = 15.3384(5) Å	
	b = 7.7329(4) Å	β= 97.275(3)°
	c = 8.5865(3) Å	
Volume	1010.25(7) $Å^3$	
Z	2	
Density (calculated)	2.204 Mg/m^3	
Absorption coefficient	7.464 mm^{-1}	
F(000)	640	
Crystal size	0.10 x 0.10 x 0.05 mm^3	
Theta range for data collection	2.677 to 28.941°.	
Index ranges	-20<=h<=20, -10<=k<=10, -10<=l<=11	
Reflections collected	14722	
Independent reflections	2493 [R(int) = 0.0500]	
Completeness to theta = 25.000°	99.9 %	
Absorption correction	Analytical	
Max. and min. transmission	0.789 and 0.610	
Refinement method	Full-matrix least-squares on F^2	
Data / restraints / parameters	2493 / 0 / 133	
Goodness-of-fit on F^2	1.088	
Final R indices [I>2sigma(I)]	R_1 = 0.0272, wR_2 = 0.0597	
R indices (all data)	R_1 = 0.0375, wR_2 = 0.0632	
Largest diff. peak and hole	1.975 and -0.864 $e.Å^{-3}$	

Table S8. Atomic coordinates (x 10^4) and equivalent isotropic displacement parameters (Ĺ^{2}x 10^3) for *trans*-dichlorobis(3-methyl-4-nitro-5-(methoxycarbonyl)isothiazole)platinum(II) (**C4**). U(eq) is defined as one third of the trace of the orthogonalized U^{ij} tensor.

	x	y	z	U(eq)
Pt(1)	5000	5000	5000	11(1)
Cl(1)	5564(1)	3088(1)	6913(1)	18(1)
S(1)	6934(1)	4218(2)	4383(1)	14(1)
N(2)	7935(3)	8867(5)	4136(4)	20(1)
O(3)	8063(3)	9229(5)	2805(4)	36(1)
O(4)	8164(3)	9735(5)	5283(4)	42(1)
N(1)	6236(2)	5758(5)	4803(4)	14(1)
C(3)	6577(3)	7344(6)	4784(5)	14(1)
C(4)	7431(3)	7296(6)	4339(5)	14(1)
C(5)	7731(3)	5672(6)	4083(5)	16(1)
C(6)	8604(3)	5131(6)	3684(5)	17(1)
O(1)	8598(2)	3458(4)	3358(4)	22(1)
O(2)	9211(2)	6109(5)	3699(4)	28(1)
C(7)	6100(3)	8917(6)	5222(5)	17(1)
C(8)	9410(3)	2775(7)	2867(6)	26(1)

Table S9. Selected Bond lengths [Å] and angles [°] for *trans*-dichlorobis(3-methyl-4-nitro-5-(methoxycarbonyl)isothiazole)platinum(II) (**C4**).

Pt(1)-N(1)#1	2.012(4)
Pt(1)-N(1)	2.012(4)
Pt(1)-Cl(1)	2.2965(10)
Pt(1)-Cl(1)#1	2.2965(10)
S(1)-N(1)	1.671(4)
S(1)-C(5)	1.704(5)
N(2)-O(4)	1.207(5)
N(2)-O(3)	1.217(5)
N(2)-C(4)	1.462(6)
N(1)-C(3)	1.334(6)
C(3)-C(4)	1.410(6)
C(3)-C(7)	1.492(6)
C(4)-C(5)	1.365(6)
C(5)-C(6)	1.485(6)
C(6)-O(2)	1.198(6)
C(6)-O(1)	1.323(5)
O(1)-C(8)	1.463(5)
N(1)#1-Pt(1)-N(1)	180.0
N(1)#1-Pt(1)-Cl(1)	91.49(10)
N(1)-Pt(1)-Cl(1)	88.51(10)
N(1)#1-Pt(1)-Cl(1)#1	88.51(10)
N(1)-Pt(1)-Cl(1)#1	91.49(10)
Cl(1)-Pt(1)-Cl(1)#1	180.0
N(1)-S(1)-C(5)	93.1(2)
O(4)-N(2)-O(3)	125.0(4)
O(4)-N(2)-C(4)	117.9(4)
O(3)-N(2)-C(4)	117.0(4)
C(3)-N(1)-S(1)	112.9(3)
C(3)-N(1)-Pt(1)	130.1(3)
S(1)-N(1)-Pt(1)	116.5(2)
N(1)-C(3)-C(4)	111.0(4)
N(1)-C(3)-C(7)	122.8(4)
C(4)-C(3)-C(7)	126.3(4)
C(5)-C(4)-C(3)	114.3(4)

C(5)-C(4)-N(2)	123.5(4)
C(3)-C(4)-N(2)	122.2(4)
C(4)-C(5)-C(6)	129.0(4)
C(4)-C(5)-S(1)	108.7(3)
C(6)-C(5)-S(1)	122.3(4)
O(2)-C(6)-O(1)	127.2(4)
O(2)-C(6)-C(5)	122.8(4)
O(1)-C(6)-C(5)	110.0(4)
C(6)-O(1)-C(8)	115.5(4)

Symmetry transformations used to generate equivalent atoms:

#1 -x+1,-y+1,-z+1

Table S10. Anisotropic displacement parameters (Ĺ2x 103) for *trans*-dichlorobis(3-methyl-4-nitro-5-(methoxycarbonyl)isothiazole)platinum(II) (**C4**). The anisotropic displacement factor exponent takes the form: $-2\pi^2[h^2 a^{*2}U^{11} + ... + 2 h k a^* b^* U^{12}]$

	U^{11}	U^{22}	U^{33}	U^{23}	U^{13}	U^{12}
Pt(1)	11(1)	11(1)	11(1)	1(1)	3(1)	1(1)
Cl(1)	20(1)	17(1)	16(1)	5(1)	3(1)	3(1)
S(1)	12(1)	13(1)	18(1)	-1(1)	4(1)	0(1)
N(2)	22(2)	18(2)	21(2)	0(2)	5(2)	-5(2)
O(3)	52(3)	35(2)	21(2)	4(2)	10(2)	-18(2)
O(4)	66(3)	36(2)	25(2)	-9(2)	9(2)	-32(2)
N(1)	11(2)	17(2)	13(2)	2(2)	4(1)	2(2)
C(3)	13(2)	16(2)	12(2)	4(2)	0(2)	-1(2)
C(4)	16(2)	14(2)	12(2)	1(2)	1(2)	-4(2)
C(5)	18(2)	16(2)	14(2)	-2(2)	4(2)	-4(2)
C(6)	16(2)	20(2)	15(2)	0(2)	3(2)	0(2)
O(1)	20(2)	17(2)	31(2)	-2(1)	10(1)	1(1)
O(2)	18(2)	28(2)	39(2)	-5(2)	9(2)	-5(2)
C(7)	18(2)	12(2)	21(2)	-2(2)	4(2)	4(2)
C(8)	19(2)	23(3)	38(3)	-5(2)	10(2)	3(2)

Table S11. Hydrogen coordinates (x 10^4) and isotropic displacement parameters (Ĺ^{2}x 10^3) for *trans*-dichlorobis(3-methyl-4-nitro-5-(methoxycarbonyl)isothiazole)platinum(II) (**C4**).

	x	y	z	U(eq)
H(7A)	6467	9941	5128	25
H(7B)	5549	9036	4517	25
H(7C)	5972	8809	6307	25
H(8A)	9343	1534	2653	39
H(8B)	9535	3374	1913	39
H(8C)	9898	2960	3705	39

Hydrogen bonds data:

	D–H	H···A	D···A	D–H···A A···H···A
Intra C(7)–H(7A)···O(4)	0.98	2.59	3.222(7)	122
Intra C(7)–H(7B)···Cl(1)^{i}	0.98	2.57	3.331(5)	135
C(8)–H(8C)···O(2)^{ii}	0.98	2.57	3.512(6)	162

Symmetry code

[i] -x,1-y,1-z

[ii] 2-x,1-y,1-z

Table S12. Cartesian coordinates of TS_{cis}.

Pt	-0.147719000000	-1.072260000000	-0.074823000000
Cl	-1.180993000000	-2.194339000000	-1.866533000000
Cl	1.294112000000	-2.857991000000	0.269336000000
Cl	-0.457411000000	-0.463128000000	2.592304000000
N	-1.544220000000	0.332152000000	-0.279015000000
S	-1.109979000000	1.777291000000	-1.038569000000
C	-2.816702000000	0.328662000000	0.080442000000
C	-3.466737000000	1.558600000000	-0.258442000000
C	-2.648310000000	2.457448000000	-0.882859000000
C	-3.412372000000	-0.855425000000	0.755914000000
H	-4.278771000000	-1.209194000000	0.199297000000
H	-2.660483000000	-1.633630000000	0.825647000000
H	-3.740798000000	-0.583461000000	1.757941000000
N	-4.852296000000	1.887269000000	0.003840000000
O	-5.247299000000	2.993348000000	-0.358042000000
O	-5.551065000000	1.055692000000	0.565480000000
H	-2.910717000000	3.441227000000	-1.228858000000
S	2.090108000000	0.912031000000	1.940853000000
N	1.687344000000	0.344668000000	0.397181000000
C	2.650578000000	0.569197000000	-0.475485000000
C	3.770553000000	1.250462000000	0.096111000000
C	3.590819000000	1.505733000000	1.430663000000
C	2.492798000000	0.099383000000	-1.883143000000
H	1.535339000000	-0.412613000000	-1.968222000000
H	2.554880000000	0.933852000000	-2.581434000000
H	3.291411000000	-0.595239000000	-2.140219000000
N	4.970317000000	1.650472000000	-0.598644000000
O	5.050454000000	1.425258000000	-1.802096000000
O	5.850479000000	2.206711000000	0.055716000000
H	4.289799000000	1.994597000000	2.087453000000

Table S13. Cartesian coordinates of TS_{trans}

Pt	0.159907000000	-0.587001000000	0.281365000000
Cl	0.175166000000	0.016259000000	2.556620000000
Cl	-0.806674000000	-2.942062000000	0.734215000000
Cl	0.185018000000	-0.881484000000	-2.053070000000
S	1.992418000000	1.990489000000	0.617575000000
N	1.897812000000	0.370505000000	0.116596000000
C	3.066957000000	-0.059395000000	-0.344729000000
C	4.068237000000	0.959056000000	-0.306307000000
C	3.620167000000	2.148635000000	0.203826000000
C	3.229955000000	-1.481773000000	-0.753042000000
H	2.322128000000	-2.018331000000	-0.489556000000
H	3.382753000000	-1.559180000000	-1.828345000000
H	4.099497000000	-1.916471000000	-0.261927000000
N	5.440251000000	0.813325000000	-0.736340000000
O	5.782426000000	-0.246173000000	-1.246403000000
O	6.190821000000	1.773379000000	-0.571417000000
H	4.195406000000	3.046016000000	0.347610000000
S	-3.159588000000	-1.502669000000	-0.462991000000
N	-2.178204000000	-0.235196000000	0.055424000000
C	-2.811024000000	0.917889000000	0.030771000000
C	-4.159389000000	0.803132000000	-0.432506000000
C	-4.482331000000	-0.492094000000	-0.752129000000
C	-2.094221000000	2.148913000000	0.473587000000
H	-1.140767000000	1.841405000000	0.893176000000
H	-1.938813000000	2.822897000000	-0.369514000000
H	-2.675910000000	2.690856000000	1.216717000000
N	-5.105917000000	1.879484000000	-0.570481000000
O	-4.730634000000	3.022793000000	-0.324136000000
O	-6.246294000000	1.597245000000	-0.935766000000
H	-5.430247000000	-0.843319000000	-1.121444000000

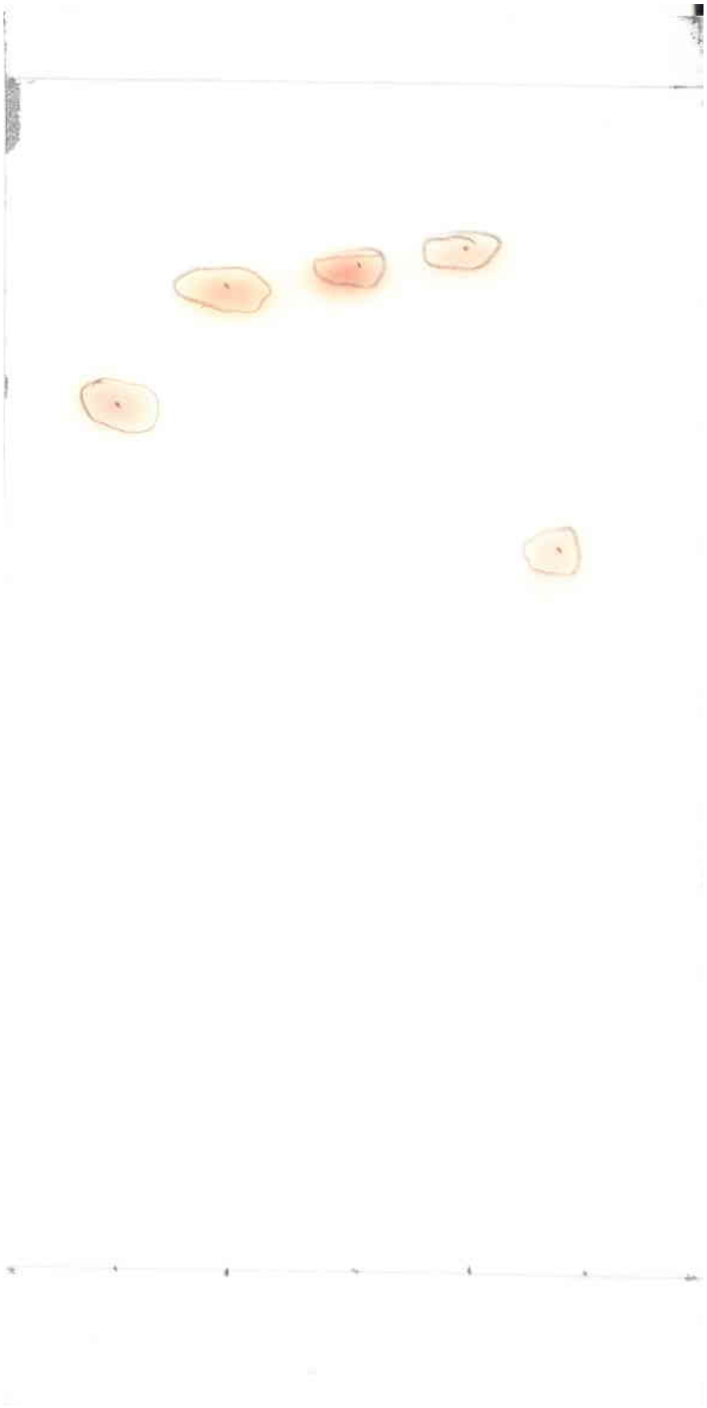

Figure S48. TLC chromatogram of complexes **C1**-**C5** (eluent chloroform:aceton 1:1 vol.). From the left *cis*-complex **C1**, *trans*-complex **C2**, *trans*-complex **C3**, *trans*-complex **C4** and *cis*-complex **C5**.

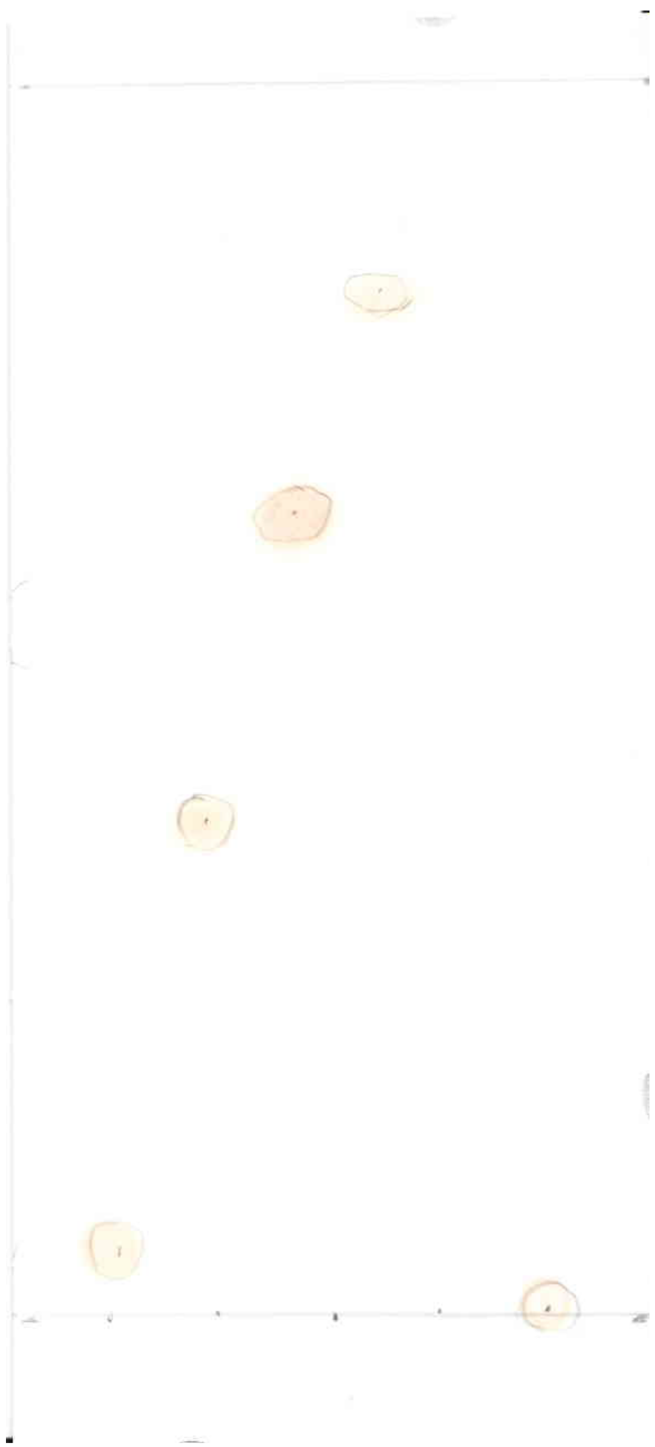

Figure S49. TLC chromatogram of complexes **C1-C5** (eluent chloroform:aceton 9:1 vol.). From the left *cis*-complex **C1**, *trans*-complex **C2**, *trans*-complex **C3**, *trans*-complex **C4** and *cis*-complex **C5**.
